# Genome-Wide Identification and Expression Analysis of the *REF* Genes in 17 Species

**DOI:** 10.3390/cimb46110701

**Published:** 2024-10-22

**Authors:** Jinkai Fang, Chi Ma, Yu Lin, Junjun Yin, Lijuan Zhu, Zhineng Yuan, Dan Zhang

**Affiliations:** 1 School of Tropical Agriculture and Forestry, Hainan University, Haikou 570228, China; fangjinkai1997@163.com (J.F.); mc1416144603@163.com (C.M.); 15008972818@163.com (Y.L.); 17680262946@163.com (J.Y.); z18314437507@163.com (L.Z.); 2 Guangdong Agribusiness Tropical Agriculture Institute, Guangzhou 511365, China; yuanzhin@foxmail.com

**Keywords:** *REF* genes, evolution, natural rubber production, rubber-producing plants

## Abstract

Natural rubber production currently relies heavily on a single species, *Hevea brasiliensis*, underscoring the urgent need to identify alternative sources to alleviate the strain on natural rubber production. The rubber elongation factor (REF) and small rubber particle protein (SRPP), both members of the *REF*/*SRPP* gene family, are crucial for natural rubber biosynthesis. However, research on the *REF* gene has predominantly focused on *H. brasiliensis* and *Taraxacum kok-saghyz*. We conducted a comprehensive genome-wide identification and characterization of the *REF* gene, identifying 87 REF protein sequences across 17 plants species. We observed a significant increase in the copy numbers and expression of *REF* genes in rubber-producing plants. Notably, in *H*. *brasiliensis*, *T*. *kok-saghyz*, *Eucommia ulmoides*, *Lactuca sativa*, and other rubber-yielding species, the number of *REF* genes has markedly increased. Furthermore, some *REF* genes in *H. brasiliensis* form a distinct clade in phylogenetic analyses and exhibit differences in conserved motif arrangements and tertiary protein structures compared to other *REF* genes. These findings suggest that *REF* genes in rubber-producing plants may have undergone independent evolution, leading to changes in copy number and structure. These alterations could contribute to the production of natural rubber in these species. The results of this study provide a scientific basis for further research into the mechanisms of rubber production in plants and for identifying potential rubber-producing species.

## 1. Introduction

*Heveabrasiliensis*, a prominent member of the Euphorbiaceae family, is renowned for its production of natural rubber, which plays a vital role in the global economy. Natural rubber (cis-1,4-polyisoprene) is an indispensable raw material for industrial production and a strategic commodity in China, known for its exceptional properties, including high elasticity, abrasion resistance, thermal conductivity, and impact resistance [1]. These characteristics make it essential in a wide range of industries, such as automotive, maritime, and aerospace, as well as in medical devices and industrial manufacturing. Despite the significance of natural rubber, research on rubber biosynthesis genes has largely been limited to major rubber-producing plants like *H. brasiliensis* and *T. kok-saghyz*, with *H. brasiliensis* currently being the only commercial source. However, other rubber-producing plants face challenges related to yield, quality, and production costs, making large-scale production difficult.

The primary reasons include the low level of automation in the industry, with the manual tapping of rubber trees remaining predominant. Additionally, the continuous rise in labor costs directly contributes to the ongoing increase in production costs for natural rubber. Furthermore, global climate changes contribute to the instability of natural rubber production, resulting in frequent fluctuations in rubber prices and significantly disrupting downstream industries [2,3,4]. According to data from the China Academy of Tropical Agricultural Sciences (CATAS) and the China Natural Rubber Association (CNRA), a significant portion of China’s natural rubber is heavily reliant on imports. China, being one of the largest consumers of rubber globally, cannot meet its domestic demand solely through domestic production, necessitating substantial importation of natural rubber to satisfy the domestic market. Given the limitations and shortages of traditional sources of natural rubber, as well as the narrow genetic base of *H. brasiliensis* production [5], there is a consensus that alternative plants should be explored to replace *H. brasiliensis* as a source of natural rubber for diversified production.

Rubber (C_5_H_8_)_n_ exists in two structural isomers: cis-polyisoprene (CPI) and trans-polyisoprene (TPI). CPI, commonly known as natural rubber [6], possesses numerous excellent physical properties, such as high elasticity, impact resistance, and good thermal conductivity. It maintains outstanding elasticity and ductility even at low temperatures. Compared to CPI, TPI exhibits slightly inferior comprehensive performance, crystallizes at room temperature, and has high hardness and tensile strength [7]. The performance of synthetic polyisoprene is inferior to natural rubber. Currently, apart from *H. brasiliensis*, only two species, *T. kok-saghyz* [8] and *Parthenium argentatum* [9], can produce a significant amount of high-molecular-weight cis-polyisoprene [10]. Few plants in nature are capable of producing trans-polyisoprene, with *E. ulmoides* being the most extensively researched species currently [11]. With the advancement of technology, there has been a considerable improvement in the production level of human society, leading to a significant increase in industrial capacity and, consequently, a continuous rise in demand for natural rubber. However, the natural rubber industry faces various challenges [12].

In nature, there are numerous rubber-producing plants, with nearly 12,500 species identified across 22 families. It is estimated that this number could reach close to 20,000 species, spanning 40 families [13]. Researchers are actively investigating the mechanisms of natural rubber biosynthesis in various plant species. *H. brasiliensis* remains the primary global source of natural rubber due to its high yield and favorable polymer properties. In addition to studying the mechanisms of natural rubber synthesis, current research efforts are also focused on improving disease resistance, particularly against fungal infections such as South American leaf blight (SALB) [14], as well as enhancing tolerance to climatic conditions [15]. Other species, such as *T. kok-saghyz* and *P. argentatum*, have garnered attention due to their ability to produce rubber in colder climates, shorter life cycles, and suitability for mechanical harvesting, making them promising alternatives for industrial-scale production [16,17]. Although *L. sativa* is not typically associated with large-scale rubber production, its ability to produce small amounts of latex has attracted interest [18]. *E. ulmoides*, which produces TPI and thrives in temperate climates, presents an alternative to traditional rubber sources [19]. *Ficus microcarpa* and *Ficus hispida* also produce natural rubber, although they are more distantly related to *H. brasiliensis* [20]. Studies on the rubber biosynthesis mechanisms of these species provide crucial insights into the evolutionary processes and biosynthetic pathways of natural rubber production in plants. However, aside from the *H. brasiliensis*, these rubber-producing plants still have shortcomings in practical applications. For instance, although the molecular weight of natural rubber in lettuce is similar to that in *H. brasiliensis*, the natural rubber content in lettuce latex is lower. Additionally, while *P. argentatum* and *T. kok-saghyz* exhibit higher natural rubber content in their latex, there are technical challenges associated with large-scale cultivation and cyclic harvesting, leading to higher production costs. Consequently, commercialization remains difficult, making them unsuitable substitutes for *H. brasiliensis* at present [21].

The natural latex produced by *H. brasiliensis*, with its molecular structure and high molecular weight (greater than 1 million daltons), exhibits excellent performance in various aspects. Even with the industrial synthesis of high-molecular-weight polymer rubber materials, there is still a performance gap compared to natural rubber. The length of these polymers determines the quality of the natural rubber, making molecular weight an important indicator for assessing usable rubber today [22].

The genetic mechanism of rubber synthesis is complex and regulated by multiple pathways and genes. This specific mechanism has not yet been thoroughly investigated. Currently, it is believed that the process of rubber synthesis involves roughly three stages: The synthesis of isopentenyl diphosphate (IPP) occurs through two pathways in *H. brasiliensis*: the cytoplasmic mevalonate (MVA) pathway and the plastidic 2-methyl-D-erythritol-4-phosphate (MEP) pathway [23]. It is widely accepted in the academic community that IPP’s main source in rubber is the MVA pathway. In the MVA pathway, it is controlled by three gene families: *ACAT*, *HMGS*, and *HMGR*. In the MEP pathway, sugar hydrolysis generates pyruvic acid, which undergoes transamination with glyceraldehyde-3-phosphate. Ultimately, through the sequential actions of six gene families—*DXS*, *DXR*, *CMS*, *MCS*, *HDS*, and *HDR*—and six enzymatic transformations, it is converted into IPP and dimethylallyl pyrophosphate (DMAPP).

The cis-polymerization of IPP and DMAPP, regulated by members of the *GPS*, *FPS*, and *GGPS* gene families, forms various 1,4-polyisoprene dimers. Later, under the action of cis-prenyltransferase (CPT) from the *CPT* gene family, IPP is continuously transferred onto the polyisoprene pyrophosphate chain [24].

Finally, under the influence of REF and SRPP, polyisoprene is polymerized into natural rubber. This is also one of the most critical links in whether plants can synthesize sudden rubber. It has been confirmed that REF and SRPP proteins play essential roles in the biosynthesis of high-molecular-weight natural rubber in plants and exhibit highly homologous amino acid sequences [25]. Additionally, they share a common structural domain within the *REF/SRPP* gene family, indicating that both belong to the same gene family [26]. Furthermore, in *H. brasiliensis*, there is a significant expansion of this gene family’s members. Immunogold electron microscopy shows that in *H. brasiliensis*, REF proteins are located in both large rubber particles (typically larger than 0.4 μM) and small rubber particles (smaller than 0.4 μM), as well as in all laticifer layers, while SRPP is mainly located in small rubber particles and the laticifer layers of the conducting bark [27]. Studies have found that REF protein has a higher affinity for the lipid membrane of rubber particles than SRPP protein. In *H. brasiliensis*, the SRPP protein is located on the surface of the lipid membrane, while the REF protein is embedded inside the membrane [28,29,30,31]. REF can aggregate into a starch-like protein that is rich in β-folds, forming large aggregates rapidly. In contrast, the SRPP protein assembles into stable nanoaggregates. Previous research suggests that SRPP may contribute to the stability of rubber particles, while REF may contribute to the aggregation of rubber particles. Experiments have shown that the REF protein can cause red blood cells to hemagglutinate and yeast cells to aggregate, while the SRPP protein can inhibit hemagglutination and prevent yeast from settling naturally [28]. This, to some extent, indicates the roles of REF and SRPP proteins in rubber particle function. Due to the critical importance of the *REF/SRPP* gene family in the synthesis of natural rubber and its status as the only expanded gene family within the biosynthetic pathway of natural rubber [32], many researchers have conducted studies on this gene family.

But as the mechanism of rubber production in plants remains incompletely understood, and *REF* is crucial in controlling rubber production in plants, their expression patterns, evolutionary relationships, and gene structures in different plants are not clear. Therefore, we conducted an exploration and comparative analysis of the REF genes in 17 plants (eight species from the Euphorbiaceae family, five non-Euphorbiaceae rubber-producing species, and four model plants). The aim of this study is to elucidate the variation patterns of the *REF* genes in rubber-producing and non-rubber-producing plants, as well as their evolutionary histories. This research will provide a scientific basis for the future production of natural rubber, and the development and utilization of germplasm resources in different rubber-producing plants.

## 2. Materials and Methods

### 2.1. Public Data Acquisition and Organization

To investigate the evolutionary relationships of the *REF* gene in plants and explore potential rubber-producing species, this study selected eight Euphorbiaceae plants: *H. brasiliensis*, *M. esculenta*, *Ricinus communis*, *Jatropha curcas*, *Vernicia fordii*, *Speranskia yunnanensis*, *Euphorbia peplus*, and *Euphorbia lathyris*. These plants include *M. esculenta*, which is closely related to *H. brasiliensis*, and other Euphorbiaceae species with high-quality reference genomes. Additionally, five rubber-producing plants were included: *T. kok-saghyz*, *E. ulmoides*, *L. sativa*, *F. microcarpa*, and *F. hispida*. These species encompass the high-quality natural rubber-producing *L. sativa* and *T. kok-saghyz*, as well as *F. microcarpa* and *F. hispida*, which are more distantly related to *H. brasiliensis* and capable of producing trans-polyisoprene (TPI). Four model plants were also incorporated: *Arabidopsis thaliana*, *Zea mays*, *Oryza sativa*, and *Solanum lycopersicum*. By using these non-rubber-synthesizing model plants as references, we aimed to study the evolutionary processes of the *REF* gene.

The genomic files, protein sequences, and GFF annotation files for the 17 plant species analyzed in this study (See Table S1), as well as the transcriptome data from the leaf tissues of each plant (See Table S2), were obtained from the NCBI (https://www.ncbi.nlm.nih.gov/; Bethesda, MD, USA; accessed on 20 August 2023) and CNCB (https://www.cncb.ac.cn/; Beijing, China; accessed on 20 August 2023) databases, as well as figshare (https://figshare.com/; London, UK; accessed on 20 August 2023). Subsequently, the Hidden Markov Model (HMM) files for the REF domain (PF05755) were downloaded from the PFAM database (http://pfam.xfam.org/; accessed on 20 August 2023). Additionally, conserved protein sequences of REF in *H. brasiliensis* and *A. thaliana* were downloaded from the NCBI website.

### 2.2. Identification of REF Genes and Modification of Genome Annotation Files

First, the conserved protein sequences of REF were downloaded from the NCBI database. We used Blastp [33] (v2.14, https://blast.ncbi.nlm.nih.gov/Blast.cgi; Bethesda, MD, USA; accessed on 21 August 2023; E ≤ 1 × 10^−100,000^) to align these sequences against the protein sequence files of the aforementioned 17 plant species. Additionally, Hmmer [34] (v3.3.2, http://hmmer.org; Cambridge, MA, USA; accessed on 22 August 2023; E ≤ 1 × 10^−100,000^) was used to search for protein sequences containing the conserved domain of the *REF* within the protein sequence files of the 17 plant species.

After obtaining the preliminary selection of sequences from both software tools, we intersected the results and submitted them to the CD-Search (the Conserved Domain Search) tool of the NCBI database to ensure that each protein sequence contains the REF domain. A total of 92 sets of REF proteins were identified from the protein files of the 17 plant species. Subsequently, manual verification of all gene annotation files was required to correct incomplete gene models or redundant annotations (See Table S3).

The IGV-GSAman software (v0.8, https://gitee.com/CJchen/IGV-sRNA; Guangzhou, China; accessed on 4 January 2024) was used to extract the corresponding gene sequences and submit them to Softberry (http://www.softberry.com; New York, NY, USA; accessed on 25 August 2023) to verify the completeness of the annotation information at these positions. We ensured the presence of complete coding sequences (CDSs), independent transcription start sites (TSS), and PolyA termination sites to confirm the correct expression of *REF* at the annotated positions. If PolyA and TSS sites were missing, we extended the existing annotation start site. Additionally, we marked incorrectly annotated information and removed duplicate annotation sites.

### 2.3. Analysis of REF Protein Domains, Motifs, Gene Structure, Physicochemical Properties, Pathways, and Subcellular Localization

The protein sequences identified as members of the *REF* genes from the 17 plant species were extracted, and their domains were validated in the NCBI database. Subsequently, the “Visualize NCBI CDD Domain Pattern” function of the TBtools [35] software (v2.0, https://github.com/CJ-Chen/TBtools; Guangzhou, China; accessed on 10 January 2024) was utilized for visualization. Simultaneously, the information of the 87 protein sequences was uploaded to the MEME website (https://meme-suite.org/; Baltimore, MD, USA; accessed on 26 August 2023) for motif analysis. The motif search frequency was set to 10, with the remaining parameters set to default. After analysis, the meme.xml and meme.htm files were downloaded, with the meme.htm file displaying motif information for subsequent analysis.

The analysis results were visualized using the “Visualize MEME/MAST Motif Pattern” function of TBtools, ensuring that each identified *REF* gene contained complete *REF*/*SRPP* family domains and motifs. Finally, the “Gene Structure View” function of the TBtools software was utilized to analyze the positions of the UTR and CDSs in each protein sequences (Figure S1). The identified 87 REF protein sequences were submitted to the ProtParam website (https://web.expasy.org/protparam/; Geneva, Switzerland; accessed on 28 August 2023) for physicochemical estimation (See Table S4). Metabolic pathways related to REF proteins were searched for in the KEGG database (https://www.kegg.jp/; Kyoto, Japan; accessed on 29 August 2023) (Figure S2) to determine the subcellular localization of the identified REF proteins using the WoLF PSORT website (https://wolfpsort.hgc.jp/; Yokohama, Japan; accessed on 29 August 2023) (See Table S5).

### 2.4. Conserved Motif Analysis of the REF Protein Sequences

To identify the motif structures of the REF protein sequences, we submitted the amino acid sequences predicted for the 87 REF proteins to the MEME website. The conserved amino acid sequences extracted from the MEME website analysis yielded three relatively conserved sequences (E ≤ 1 × 10^−1000^), labeled as Motif5, Motif1, and Motif2. A multiple sequence alignment was performed by MUSCLE [36] (v5.1, https://www.drive5.com/muscle/; San Francisco, CA, USA; accessed on 13 September 2023) and the alignment results were visualized using Jalview [37] (v2.11, https://www.jalview.org/; Cambridge, UK; accessed on 15 September 2023).

### 2.5. Construction of REF Gene Phylogenetic Tree

The alignment results were trimmed by TrimAl [38] (v1.4.1, http://trimal.cgenomics.org/; Amsterdam, Netherlands; accessed on 20 September 2023) with parameters set to automated1, gappyout, gt 0.8, st 0.001, and cons 60. The trimmed protein sequences were then used to construct an evolutionary tree via the Neighbor-Joining method in TreeBest [39] (v1.92, https://github.com/Ensembl/treebest; Cambridge, UK; accessed on 28 September 2023) (treebest nj -W -t jtt -b 1000). The resulting tree was submitted to the iTOL website (https://itol.embl.de/; Heidelberg, Germany; accessed on 15 January 2024) for visualization, depicting the topology and phylogenetic relationships.

### 2.6. Prediction of REF Proteins’ Tertiary Structure

The identified 87 protein sequences were extracted, and their tertiary structures were predicted by the AlphaFold2 [40] program. The spatial configurations with the highest confidence level (the scores ranked 001) among the predicted results were opened by the PyMOL [41] software (v3.0, https://pymol.org/2/; Cambridge, MA, USA; accessed on 25 January 2024).

### 2.7. Chromosomal Localization of the REF Genes

First, we imported the annotation files of each species into the “Gene Density Profile” tool in TBtools to calculate the gene density of the entire genome, and exported the gene density statistics in an XLS format. Then, we extracted the identified *REF* gene IDs and their corresponding GTF annotation files from each species. We imported these files, along with the gene density statistics, into the “Gene Location Visualization from GTF/GFF” tool in TBtools.

### 2.8. Collinearity Analysis of the REF Genes

We conducted an interspecific collinearity analysis at the chromosomal level across 15 species (the genome sequences of *V. fordii* and *J. curcas* are contigs that do not reach the chromosomal level). Firstly, the genome files and GFF files containing the gene position information from the 17 plant species were submitted to the “one-step MCScanX” program in TBtools. After the program completed its run, the collinearity and CTL result files were generated. The collinearity file primarily displays information about collinear blocks, while the CTL file contains the necessary gene information, correspondence between genomes, and analysis parameters. Subsequently, the collinearity and CTL files, along with the GFF annotation files and identified REF gene IDs from each species, were submitted to the “Dual Synteny Plot” program in TBtools. Finally, the MCScanX software (v10.0, https://github.com/wyp1125/MCScanX; San Francisco, CA, USA; accessed on 26 January 2024) was used to visualize the results of the collinearity analysis.

### 2.9. Analysis of REF Gene Expression Patterns Based on Transcriptome Data

Transcriptome data for Reyan73397, RRIM929, and PB260, as well as published transcriptome data from the flowers, leaves, bark, and latex of common rubber cultivars such as GT1, were downloaded from the NCBI database. Additionally, transcriptome data from leaf tissues of various plants were included for analysis (See Table S6). The plant reference genome index was constructed using Hisat2 [42] (v2.2.1, https://daehwankimlab.github.io/hisat2/; Madison, WI, USA; accessed on 27 January 2024). The content of the SAM files was sorted by chromosome and position using Samtools [43] (v1.19.2, https://samtools.sourceforge.net/; Cambridge, UK; accessed on 27 January 2024) software, and the sorted results were saved in BAM format. Indexes were constructed for the BAM files. The expression levels were calculated using the featureCounts function in the Subread [44] software (v2.06, https://subread.sourceforge.net/; Melbourne, Australia; accessed on 5 February 2024). Expression data from each sample were merged into an expression matrix. Expression data for *REF*/*SRPP* family members were extracted for differential analysis, and heatmaps were generated using the “pheatmap” package in the RStudio [45] (v1.1.4, https://www.rstudio.com/tags/rstudio-ide/; Boston, MA, USA; accessed on 6 February 2024).

## 3. Results

### 3.1. Identification of REF Genes in 17 Plant Species

To explore the origin and evolutionary history of *REF* genes in plants, we employed a comprehensive approach using Hidden Markov Models (HMM) and BLAST (E ≤ 1 × 10^−100,000^) identification methods to search for REF protein sequences in the genomes and protein databases of 17 species. Ultimately, we identified 87 REF proteins across these 17 species (Table S1). Among these, sixteen sequences (18.4%) were from *H. brasiliensis*, twelve sequences (13.8%) were from *L. sativa*, nine sequences (10.3%) were from *T. kok-saghyz*, and nine sequences (10.3%) were from *E. ulmoides*. These four rubber-producing plants contributed 52.9% of the total *REF* sequences identified. Compared with non-rubber-producing plants, *REF* genes exhibit significantly higher copy numbers in rubber-producing plants, particularly in *H. brasiliensis*, which has the highest number of copies. This suggests that the number of *REF* genes may influence the rubber synthesis capability of these plants. Subsequently, we analyzed the conserved domains and motifs of these proteins.

**Figure 1:**
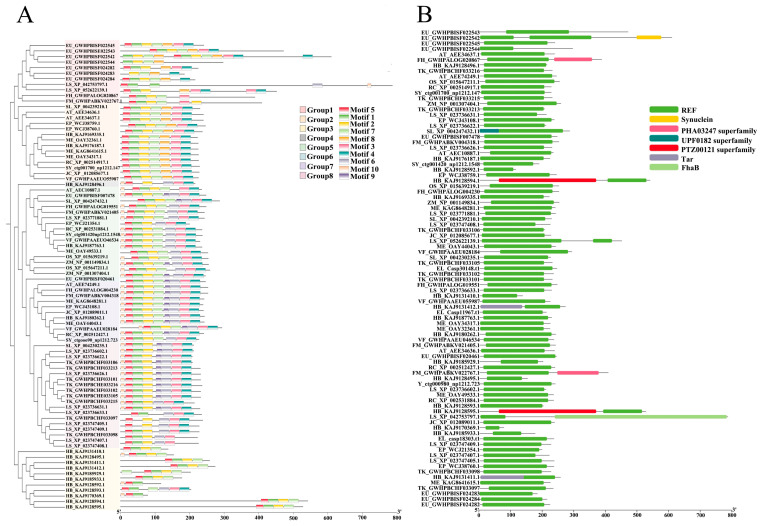
Visualization of domain, motif, and gene structure analysis of 87 *REF* genes. (**A**) Motif identification plot for *REF* family. (**B**) Conservation domain identification plot for *REF* family. Green represents the structural domain of the *REF* family.

We identified a total of 10 conserved motifs in the REF proteins, with Motif5 (97.8%, 85/87), Motif1 (95.4%, 83/87), and Motif2 (94.3%, 82/87) being the most conserved. We considered the presence of these conserved motifs in the protein sequence to be one of the criteria for identifying REF proteins. Based on the arrangement of the 10 motifs, we classified the 87 REF protein sequences into eight groups (labeled as Group 1 to Group 8) (Figure 1A). These REF protein sequences generally share a variable 5′ end and a relatively conserved 3′ end, with 81.6% of the proteins containing Motif4 at the 3′ end.

Seven conserved domains were identified through analysis of the *REF* family’s conserved structural domains. As observed in Figure 1B, all REF protein sequences contain the conserved structural domains of the *REF* family.

### 3.2. Conservation Analysis of REF Protein Conserved Amino Acid Sequences

**Figure 2:**
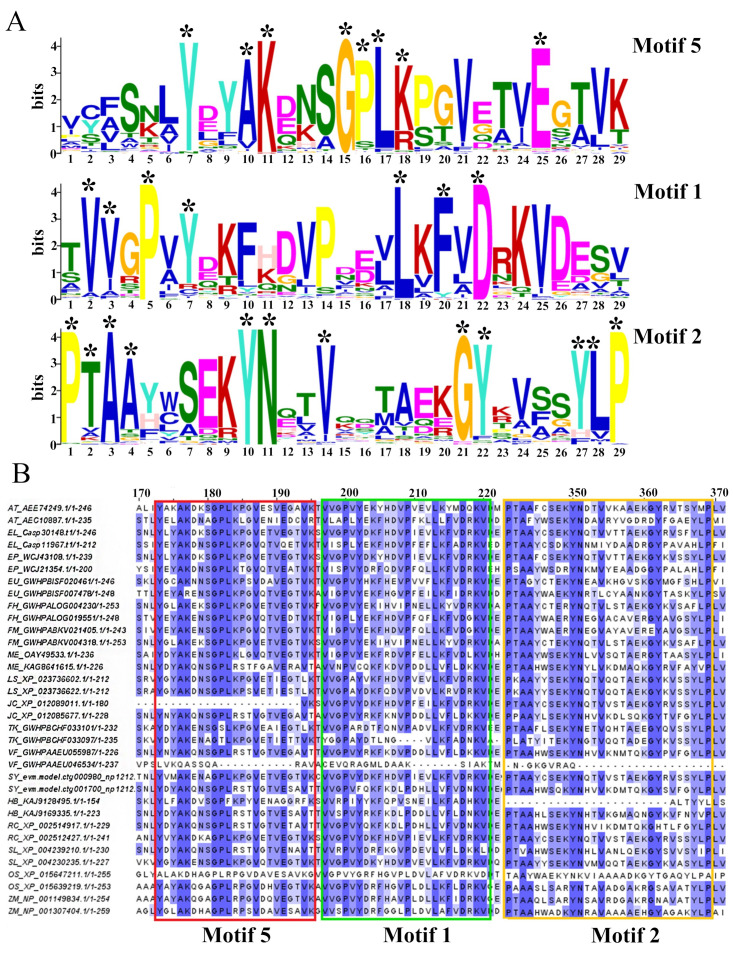
Conservation analysis of REF protein conserved amino acid sequences. (**A**) Amino acid sequences surrounding conserved residues in 17 species. The overall height of the letters represents the sequence conservation at each position, and the height of the letters reflects the relative frequency of the corresponding amino acid at that position. “*” indicates highly conserved amino acid residues. (**B**) Multiple sequence alignment of identified REF protein members, with highly conserved amino acid residues highlighted by red, green, and yellow boxes for emphasis.

Three relatively conserved amino acid sequences (E ≤ 1 × 10^−1000^) were extracted (Figure 2A). The protein sequences of these 87 members were aligned using the Muscle software and visualized using the Jalview software (Figure 2B and Figure S3). It was observed that in Motif5, REF protein sequences contain four highly conserved amino acids (Y7, K11, G15, and E25) and four relatively conserved amino acids (A10, P16, L17, and K18). In Motif1, there are three highly conserved amino acids (P5, L18, and D22) and four relatively conserved amino acids (V2, V3, Y7, and F20). Motif2 contains five highly conserved amino acids (P1, A3, Y10, N11, and P29) and seven relatively conserved amino acids (T2, A4, V14, G21, Y22, Y27, and L28). Based on the similarity of these sequences across different species, we can be infer that the *REF* gene has undergone some divergence during evolution, but the biological functions of the homologous genes may still be similar.

### 3.3. Physicochemical Properties Analysis of REF Proteins

We conducted an analysis of the physicochemical properties of these 87 proteins and found that the lengths of the amino acid sequences ranged from 79 to 787 residues (with an average of 255 amino acids), resulting in considerable variation in molecular weight, ranging from 8.92 kDa to 82.14 kDa (average 27.85 kDa). Regarding isoelectric point (pI) analysis, the pI values of the 87 REF proteins ranged from 4.26 to 9.41 (with an average of 6.75), with 51 proteins having pI values below 7 and 36 proteins having pI values above 7. In terms of instability coefficient, among these proteins, 23 had instability indices greater than 40, indicating instability, while the other proteins were relatively stable. Regarding hydrophilicity, most proteins had hydrophilicity indices below 0, indicating hydrophobicity, with only 17 of the proteins being hydrophilic. Among these 17 hydrophilic proteins, seven were distributed in *E. ulmoides* and seven were distributed in *H. brasiliensis* (Table S4). These hydrophilic proteins are predominantly found in rubber-producing plants. Subcellular localization analysis showed that REF proteins are primarily distributed in cytoplasmic vesicles and the cytoskeleton (Table S6). We speculate that this hydrophilic property allows REF proteins to more effectively perform their function of extending small rubber particles.

### 3.4. Phylogenetic Relationship of REF Proteins in Different Species

**Figure 3:**
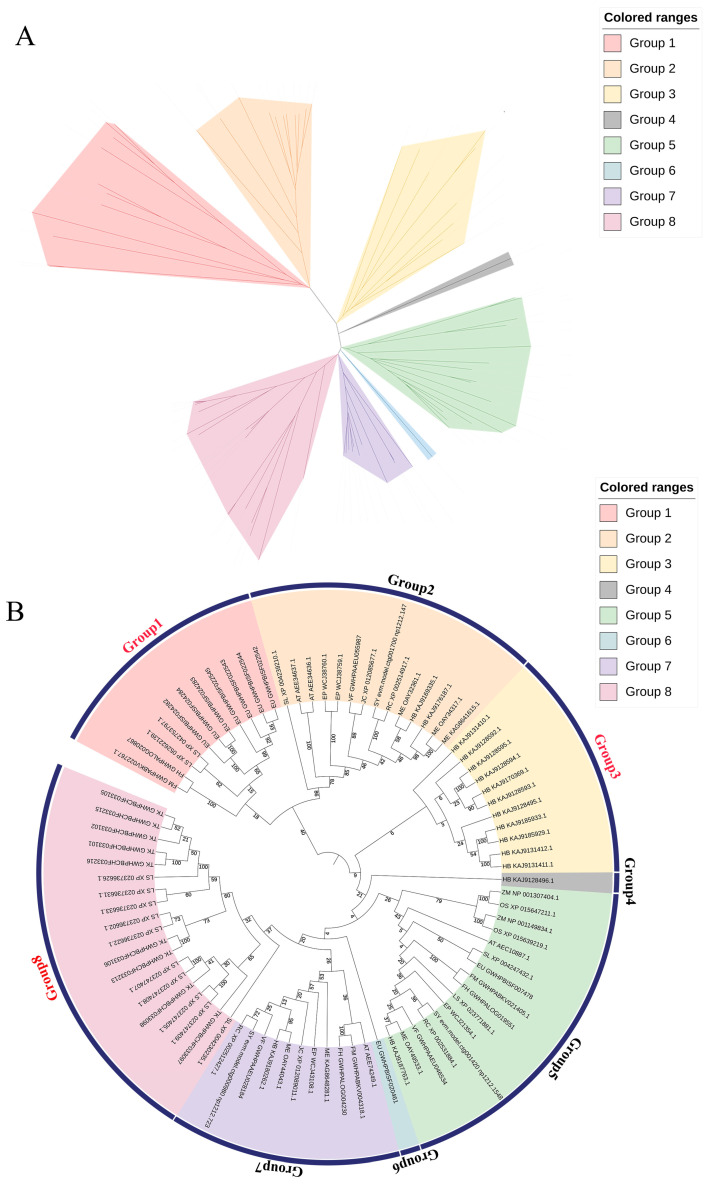
Phylogenetic trees of REF protein sequences in 17 Species. (**A**) Neighbor-Joining phylogenetic tree of the REF protein. (**B**) Phylogenetic relationships of the REF protein in 17 species.

We constructed a Neighbor-Joining phylogenetic tree for the identified sequences of the 87 REF proteins. The simplified phylogenetic tree, shown in Figure 3A, mainly illustrates the relationships between different clusters, while the complete phylogenetic tree is presented in Figure 3B. In Figure 3B, REF proteins identified from rubber-producing plants tend to cluster together on an evolutionary branch. Group 1, Group 3, and Group 8 are primarily composed of REF proteins identified from rubber-producing plants.

In Group 1, the members mainly consist of REF protein sequences from *E. ulmoides* of the Eucommiaceae family, *T. kok-saghyz* of the Asteraceae family, and *F. microcarpa* and *F. hispida* of the Moraceae family. It is noteworthy that all 11 sequences in Group 3 originate from *H. brasiliensis*, and according to the motif analysis mentioned earlier, the motif arrangement of REF protein sequences in this group differs from those found in other plants, suggesting that this species may have undergone a unique evolutionary process. In Group 8, except for one member from Solanaceae *S. lycopersicum*, all members come from Asteraceae *T. kok-saghyz* and *L. sativa*.

### 3.5. Prediction of REF Protein Tertiary Structures

**Figure 4:**
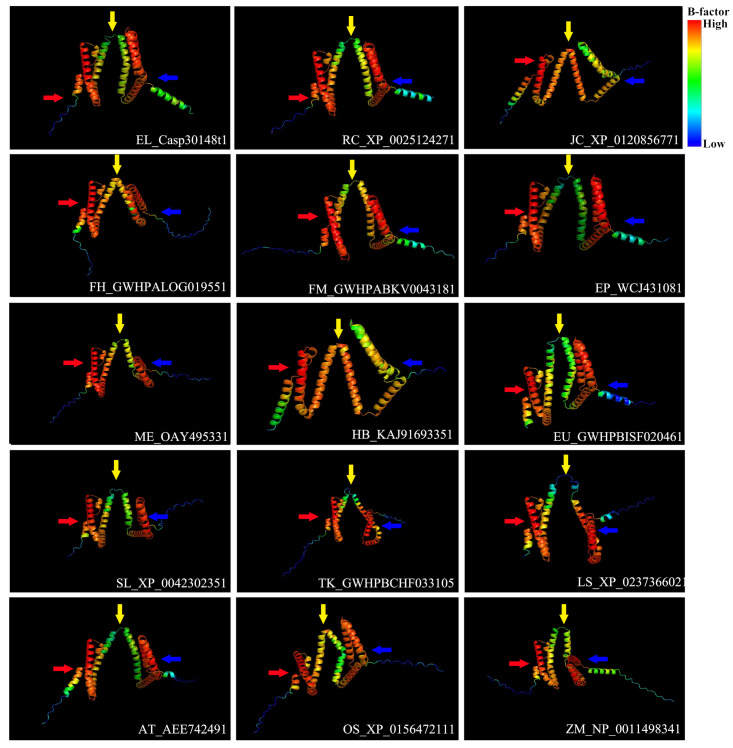
Tertiary structure of *REF* proteins predicted by AlphaFold2. In the protein structure, the regions in red have higher B-factors, while the regions in blue have lower B-factors. Red, yellow, and blue arrows represent three typical folding patterns. Protein IDs are located in the bottom right corner, and Latin abbreviations are used to distinguish species.

We used the artificial intelligence program AlphaFold2 to predict the protein structures (File S1). Analysis of the predicted tertiary structures revealed that most REF proteins exhibit similar spatial folding patterns, with three typical folding conditions (Figure 4). Additionally, it was observed that species with closer phylogenetic relationships, such as *M. esculenta* and *H. brasiliensis*, *T. kok-saghyz* and *L. sativa*, and *O. sativa* and *Z. mays*, show a high degree of structural similarity in their REF proteins, particularly in three common folding patterns observed near the N-terminus, C-terminus, and central region of the proteins, as highlighted in red, blue, and yellow in Figure 4. It is hypothesized that this structural similarity across different plant species may lead to the *REF* gene performing similar functions.

### 3.6. Chromosomal Localization

**Figure 5:**
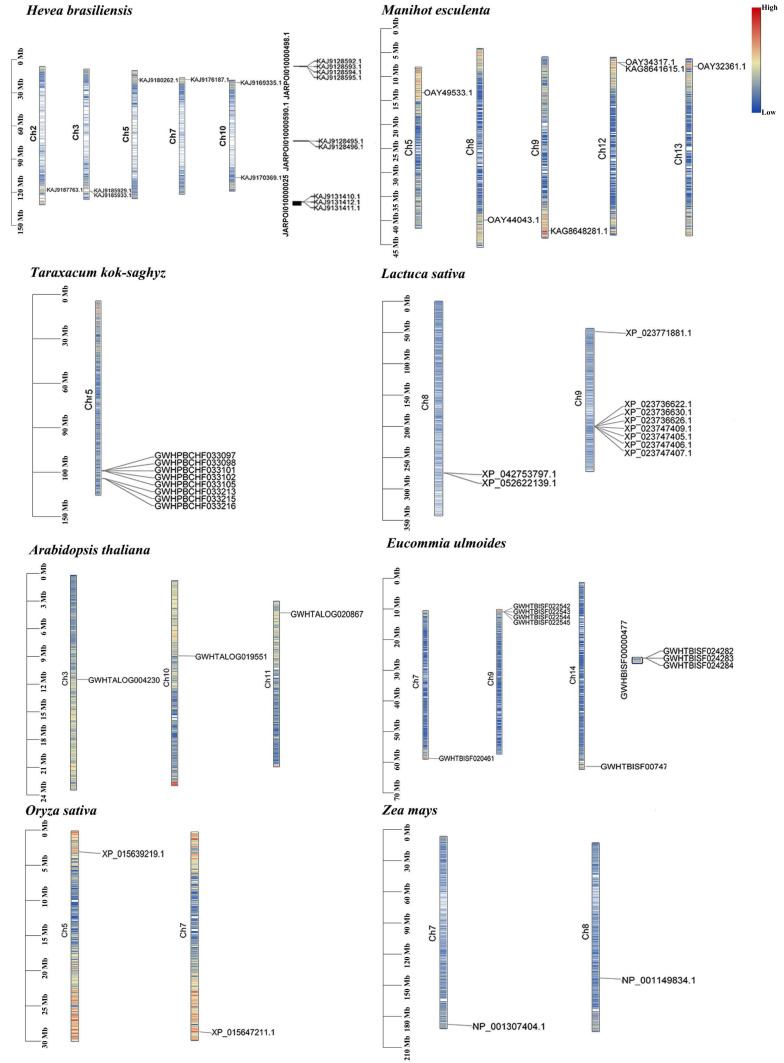
Genomic localization of *REF* genes on chromosomes. The figure illustrates the genomic positions of *REF* genes on chromosomes, with filled colors indicating the heat map of gene density. Blue fill indicates regions with low gene density, while red fill indicates regions with high gene density.

Chromosomal localization analysis (Figure 5, File S2) reveals a significant amplification of *REF* genes in latex-producing plants compared to other species. In Euphorbiaceae species, such as *H. brasiliensis*, *REF* genes are evenly distributed at the beginning and end of multiple chromosomes. In *H. brasiliensis*, these genes are located on chromosomes 2, 3, 5, 7, 9, and 10, with six located on these chromosomes and nine unanchored. Notably, the hydrophilic proteins identified in the physicochemical characterization of *H. brasiliensis* are not localized on chromosomes. Further research is needed to determine the exact reasons behind this observation. In *M. esculenta*, *REF* genes are distributed on chromosomes 5, 8, 9, 12, and 13. In *E. ulmoides*, except for three sequences unanchored to any chromosome (for the same reason mentioned above; in the latest version of the *E. ulmoides* reference genome selected for this study, these genes were not included during assembly), two others are distributed on chromosomes 7 and 14, respectively, with the rest on chromosome 9, encoding hydrophilic protein sequences. In latex-producing Asteraceae species, including *A. thaliana* and *L. sativa*, *REF* genes tend to cluster on a single chromosome, while in *T. kok-saghyz*, they tend to concentrate on chromosome 5, in *L. sativa* on chromosome 8, and two additional genes on chromosome 9. In other plants, *REF* genes are distributed on three chromosomes. In plants of the Poaceae, such as *O. sativa* and *Z. mays*, identified *REF* genes are distributed on two different chromosomes, with *O. sativa* on chromosomes 5 and 7, and *Z. mays* on chromosomes 7 and 8. We found that in latex-producing plants such as *H. brasiliensis*, *E. ulmoides*, *L. sativa*, and *T. kok-saghyz*, multiple *REF* genes tend to be located on a single chromosome, a pattern not observed in other non-latex-producing plants. Based on the preceding results, it is hypothesized that the total copy numbers of *REF* genes and their frequencies on individual chromosomes are positively correlated with the capacity of plants to produce natural rubber.

### 3.7. Cross-Species Collinearity Analysis of REF Genes

**Figure 6:**
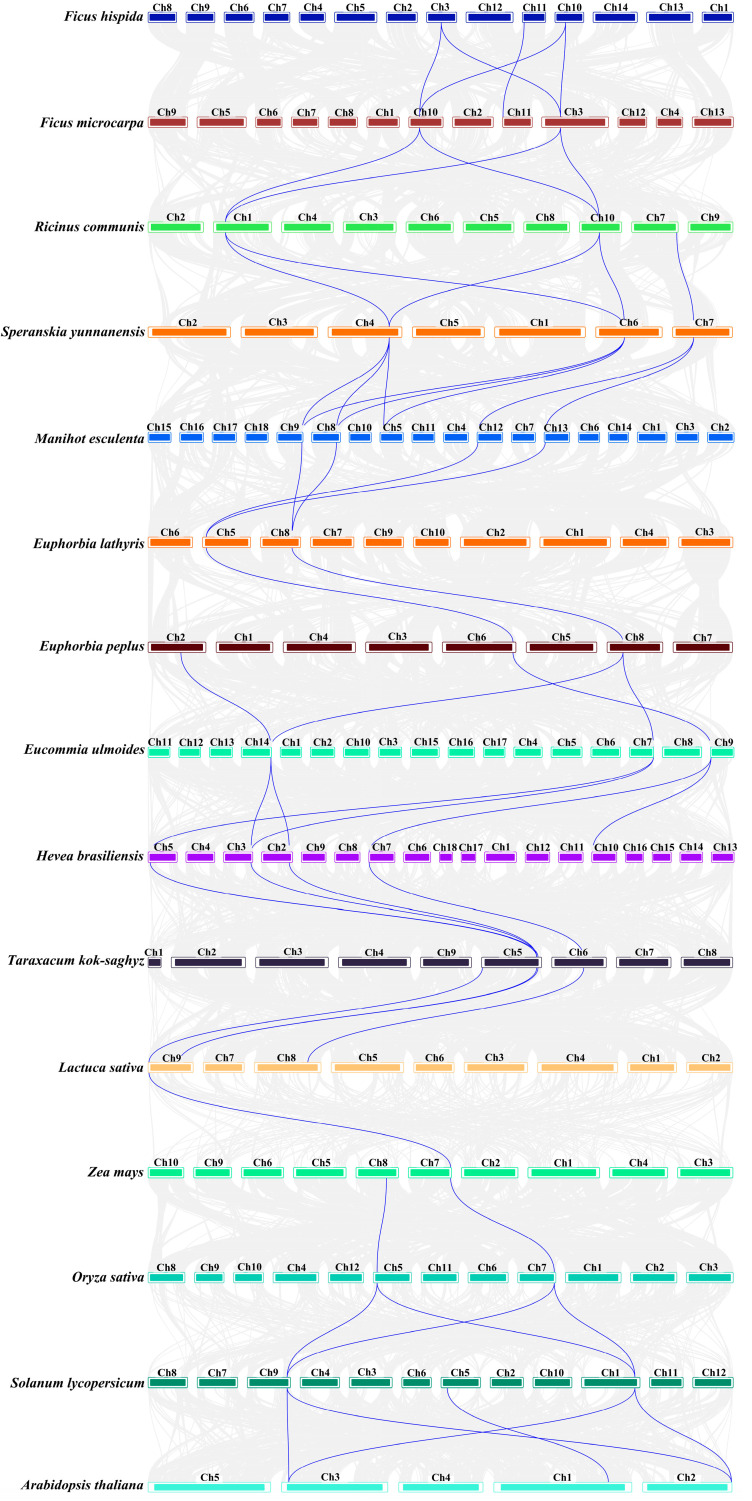
Homology analysis of *REF* genes in 17 species. The homology analysis plot shows the homologous relationships among *REF* genes in 17 species, differentiated by the initials of the Latin names.

We conducted an interspecific collinearity analysis at the chromosomal level across 15 species. The results of the collinearity analysis (Figure 6) revealed that in the extensively studied rubber-producing plants *H. brasiliensis* and *T. kok-saghyz*, four pairs of homologous *REF* sequences were located on chromosomes 5, 2, 3, and 7 in *H. brasiliensis*. In *H. brasiliensis* and *M. esculenta*, six pairs of homologous *REF* sequences were identified, located on chromosomes 5, 3, 2, 7, and 10 in *H. brasiliensis*, and chromosomes 7, 9, and 14 in *M. esculenta*. In the rubber-producing plants *L. sativa* and *T. kok-saghyz*, both belonging to the Asteraceae family, four pairs of homologous *REF* sequences were identified, located on chromosomes 8 and 9 in *L. sativa*, and chromosomes 5 and 6 in *T. kok-saghyz*. Significant collinearity was also found among other Euphorbiaceae members, including *M. esculenta*, *V. fordii*, *R. communis*, and *E. lathyrism*.

Furthermore, in the collinearity analysis between *H. brasiliensis* and 14 other plants (File S3), it was found that the *REF* genes in *H. brasiliensis* and other plants also exhibit significant synteny; for example, homologous *REF* genes on chromosomes 5 and 7 in *O. sativa* showed collinearity with those on chromosomes 5 and 2 in *H. brasiliensis*. This finding seems to confirm the widespread presence of *REF* genes in plants and their relatively close phylogenetic relationships. Despite chromosomal rearrangements and gene recombinations occurring throughout plant evolution, this study identifies a strong collinearity among *REF* genes across various plants, indicating a high degree of homology among *REF* genes. Furthermore, *REF* genes exhibit greater collinearity in rubber-producing plants or those closely related, suggesting that these genes may share similar functions and origins.

### 3.8. Expression Analysis of REF Genes

**Figure 7:**
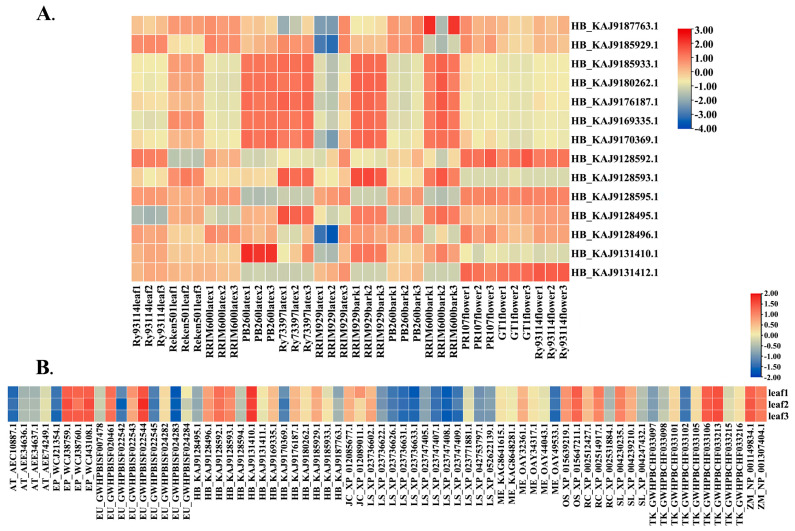
Expression analysis of *REF* genes in different species. Red represents relatively high expression levels of corresponding genes, while blue represents relatively low expression. (**A**) Expression levels of *REF* genes in leaves, latex, bark, and flower tissues of different cultivated varieties of *H. brasiliensis*. The x-axis represents different varieties of *H. brasiliensis* and different parts, while the y-axis represents different *REF* genes in *H. brasiliensis*. (**B**) Expression levels of *REF* genes in leaf tissues of various species. The x-axis represents three sets of transcriptome samples of leaves, and the y-axis represents *REF* genes, differentiated by abbreviations of Latin names to represent species.

We conducted a quantitative expression analysis of the identified *REF* genes in different plants (Figure 7). Figure 7A shows the expression levels of *REF* genes in bark, flower, leaf, and latex tissues of various high-yielding *H. brasiliensis* varieties. The results indicate that the expression levels of *REF* genes are relatively high in the latex tissues of high-yielding *H. brasiliensis* varieties Reyan73397 and RRIM929, with HB_KAJ9185933.1 showing the highest expression in Reyan73397 and HB-KAJ9170369.1 showing the highest expression in RRIM929. Additionally, the expression levels of HB_KAJ9128495.1, HB_KAJ9128593.1, HB_KAJ9128495.1, HB_KAJ9131410.1, and HB_KAJ9131412.1 in the latex tissues of Reyan73397 and RRIM929 are higher than those in the latex tissues of PB260 and RRIM600, leading us to speculate that these genes may be associated with the high-yield characteristics of Reyan73397 and RRIM929. Moreover, when comparing the expression levels of HB_KAJ9128592.1, HB_KAJ9128593.1, HB_KAJ9128594.1, HB_KAJ9131410.1, and HB_KAJ9131411.1 in bark tissues of RRIM929 and PB260, it was found that the expression levels in RRIM929 were higher than those in the low-yielding rubber PB260, suggesting their potential relevance to rubber production. Figure 7B shows the expression profiles of *REF* genes in the leaf tissues of different plants. It was observed that plants with high rubber yields, such as *H. brasiliensis*, *T. kok-saghyz*, and *E. ulmoides*, exhibit high levels of expression of *REF* genes in their leaf tissues. Conversely, in the leaf tissues of *L. sativa*, most *REF* genes are expressed at lower levels. This might contribute to *L. sativa’s* lower production of high-molecular-weight latex, though further research is needed to explore the specific causes.

## 4. Discussion

In this study, we identified and characterized *REF* genes across 17 plant species. Our findings indicate that the copy numbers of *REF* genes are significantly higher in rubber-producing plants compared to non-rubber-producing plants. Additionally, in regard to protein structure, while some REF proteins across different species exhibit similar folding patterns, certain REF proteins in high rubber-yielding plants, such as *E. ulmoides* and *H. brasiliensis*, demonstrate distinct differences in folding and motif arrangements compared to those in other plants. We hypothesize that the *REF* gene may have undergone an independent evolutionary process in rubber-producing plants, resulting in increased copy numbers and structural variations in certain genes. These changes may have enabled specific plants to acquire the ability to synthesize natural rubber.

### 4.1. Distribution of REF Genes in Plants

Based on the latest genome sequences and annotation files, we identified 87 *REF* genes from 17 plant species. Most of the genes are located on chromosomes; however, the nine *REF* genes in *H. brasiliensis* and the three *REF* genes in *E. ulmoides* are not situated on any chromosome. An examination of the genome files revealed that, in the selected versions of the reference genomes for *H. brasiliensis* and *E. ulmoides* used in this study, these genes were not incorporated during the assembly process. Although the *REF* gene is present in all 17 plant species involved in this study, analysis revealed that the copy numbers of *REF* genes are significantly higher in rubber-producing plants compared to non-rubber-producing plants. Notably, *H. brasiliensis* exhibits the highest copy number among these species. Gene duplication events are significant mechanisms in plant evolution, leading to the expansion of gene family members [46,47]. Previous studies have indicated that most plant species have experienced gene duplication or polyploidization events [42,43]. Tang et al. utilized Illumina GA2 and HiSeq 2000, as well as mate-pair sequencing, to sequence the cultivated rubber variety reyan73397 and identified 18 *REF/SRPP* family genes from it [44]. We utilized the *H. brasiliensis* reference genome, which was obtained through the deep sequencing of wild *H. brasiliensis* (MT/VB/25A 57/8) by Cheng et al. [45]. This genome was generated using Illumina sequencing (Illumina, Inc., San Diego, CA, USA), SMRT sequencing (Pacific Biosciences (PacBio), Menlo Park, CA, USA), Bio-Nano (Bionano Genomics, San Diego, CA, USA) data, and Hi-C data, from which we identified 16 members of the *REF* genes. Ding et al. [48], hypothesized that the *REF* genes initially shared a common origin in plants, followed by varying degrees of duplication events in different plants, leading to evolutionary divergence. This divergence resulted in distinct numbers of *REF* genes in *H. brasiliensis* and other plants due to specific tandem repeats within the former. Based on our identification of *REF* genes in various plants, two *REF* gene members have been identified in *O. sativa* and *Z. mays*, while three-to-five *REF* gene members have been identified in Euphorbiaceae plants such as *M. esculenta*, *R. communis*, and *S. lycopersicum*. This conclusion aligns with Ding’s findings of one-to-two *REF/SRPP* family members in *O. sativa* and *Z. mays*, and three-to-five members in Euphorbiaceae plants. Overall, the *REF* gene exhibits significant amplification in high rubber-producing plants such as *H. brasiliensis*, *T. kok-saghyz*, *L. sativa*, and *E. ulmoides*, which synthesize high-molecular-weight natural rubber. However, in *M. esculenta*, we identified six *REF* genes distributed across five chromosomes, representing the highest number of *REF* genes found in non-rubber-producing plants. Despite *M. esculenta* being the species currently known to be most closely related to *H. brasiliensis*, and although its *REF* genes have undergone a certain degree of amplification, it does not possess strong capabilities for natural rubber synthesis. The specific reasons for this limitation require further investigation. We hypothesize that in non-rubber-producing plants, the initial number of *REF* gene members is relatively low, and the probability of duplication events is also lower. In some plants, during evolution, *REF* genes underwent duplication events, resulting in increased gene copy numbers. It is likely this unique evolutionary process that enabled rubber-producing plants to acquire the ability to biosynthesize natural rubber.

### 4.2. Structure and Evolutionary Relationships of REF Protein in Plants

Genes with similar structures and conserved motifs generally exhibit similar functions [49,50]. The similar arrangement of motifs forms the basis for gene family classification and functional differentiation [51,52]. In this study, 87 REF protein sequences identified from 17 plant species were classified into eight groups, with genes within the same group exhibiting similar motif arrangements. Among the 10 identified motifs, motif5, motif2, and motif1 are highly conserved. However, in Group 5, the motif arrangement of REF proteins identified in *H. brasiliensis* is markedly different from that of other plants, with these proteins showing higher variability at the 3′ end. This unique arrangement is also observed in some *L. sativa* and *E. ulmoides* sequences in Group 1, as well as in some *L. sativa* and *T. kok-saghyz* sequences in Group 7. However, in non-rubber-producing plants, such structural variations do not appear. We hypothesize that the *REF* genes in rubber-producing plants have undergone independent evolution, resulting in structural changes in some REF proteins that may influence the plants’ ability to produce rubber.

### 4.3. Phylogenetic Relationships of REF Genes in Different Plants

We constructed a phylogenetic tree using the Neighbor-Joining method and classified these genes into eight groups based on their topological structures. We observed distinct clustering patterns of *REF* genes in rubber-producing plants compared to non-rubber-producing plants, particularly evident in Group 1, Group 3, and Group 8, where the main members were from rubber-producing plants such as *E. ulmoides*, *T. kok-saghyz*, and *L. sativa*. Notably, all members in Group 3 were from *H. brasiliensis*. In Groups 2, 5, and 6, the *REF/SRPP* family members in rubber exhibited closer phylogenetic relationships with *M. esculenta*, while *J. curcas*, *V. fordii*, and *S. peplus*, which are non-rubber-producing Euphorbiaceae plants, showed closer phylogenetic relationships. Tang et al. [44] discovered a distinct group of *REF* members and two *SRPP* members in *H. brasiliensis* that clustered separately from other plants on the phylogenetic tree and were unique to *H. brasiliensis*. Key events in the evolution of the *REF*/*SRPP* gene family have not been widely confirmed [48], with recent speculation suggesting an independent evolutionary process within *H. brasiliensis* [53]. Xia et al. [54] conducted a comparative genomic analysis of three major beverage plants, tea (*Camellia sinensis*), cacao, and coffee, and identified a recent rapid and independent evolution in the caffeine biosynthetic pathway of the tea plant. However, the evolution of the natural rubber biosynthetic pathway in latex-producing plants remains largely unexplored. Combining phylogenetic tree analysis and gene motif analysis, we hypothesize that latex-producing plants such as *H. brasiliensis*, *E. ulmoides*, *T. kok-saghyz*, and *L. sativa* in groups Groups 1, 5, and 8 exhibit a unique relationship between motifs and REF protein sequences compared to other plants. We hypothesize that *REF* genes in plants may have originated from a common ancestor and subsequently underwent different evolutionary processes within their respective taxa, such as changes in gene copy numbers and protein structures. These evolutionary changes may have endowed rubber-producing plants with the unique ability to synthesize natural rubber.

### 4.4. Tertiary Structure of REF Proteins

Differences in protein sequences and structures reflect differences in protein functions [55]. In recent years, artificial intelligence technology has rapidly advanced and been widely applied in various fields. For example, the collaboration between the DeepMind team and the European Molecular Biology Laboratory has led to the development of AlphaFold 2, an AI program capable of predicting the tertiary structures of proteins accurately based on their amino acid sequences. Its accuracy rivals that of experimental techniques, such as cryo-electron microscopy, nuclear magnetic resonance, and X-ray crystallography [56]. We applied this cutting-edge technology to predict the structures of REF proteins. In our study, we identified three relatively conserved spatial conformations among REF proteins across the 17 species analyzed. However, REF proteins in some high-rubber-yielding plants, such as *H. brasiliensis* and *E. ulmoides*, exhibit unique spatial conformations. Additionally, REF protein sequences from closely related genera exhibit greater structural similarity. According to the collinearity analysis, these REF proteins demonstrate more pronounced collinearity within closely related genera. We hypothesize that *REF* genes have a common origin in plants and have undergone independent evolution in different genera, leading to specific structural variations in rubber-producing plants that enable these plants to synthesize natural rubber.

### 4.5. Transcriptomic Analysis of REF Gene Expression Profiles in Different Plants

As a key factor in natural rubber synthesis, *REF* gene expression is positively correlated with rubber yield [57,58]. Rubber-producing plants exhibit significantly higher levels of *REF* gene expression compared to non-rubber-producing plants. Notably, the expression levels of *REF* genes in *T. kok-saghyz* and *H. brasiliensis* are significantly higher than in other plants. Furthermore, although 12 *REF* genes were identified in lettuce, their expression levels in leaf tissues are comparatively low, which may also contribute to the production of only small amounts of high-molecular-weight natural rubber [59].

## 5. Conclusions

In conclusion, our study presents a comprehensive genome-wide identification and characterization of *REF* genes across 17 plant species, revealing significant insights into their evolutionary dynamics and functional implications. We identified 87 REF protein sequences, classifying them into eight distinct groups based on their topological structures. Our findings indicate a marked expansion of *REF* genes in rubber-producing plants compared to their non-rubber-producing counterparts, with a positive correlation observed between *REF* gene copy numbers, the quantity of natural rubber synthesized, and the molecular weights of natural rubber particles. Phylogenetic analysis further reveals that REF proteins from rubber-producing plants form distinct, independent clusters, differentiating them from non-rubber-producing species; one branch is composed exclusively of proteins from *H. brasiliensis*. While REF proteins generally exhibit three typical folding patterns, unique spatial conformations were identified in high-rubber-yielding species such as *H. brasiliensis* and *E. ulmoides*. Based on these observations, we speculate that *REF* genes have undergone independent evolution in rubber-producing plants, leading to the amplification of *REF* gene numbers and structural variations. These variations may enable certain plants to acquire the capacity for natural rubber synthesis. Despite these contributions, our study is limited by its focus on only 17 species, which may not fully capture the diversity of *REF* gene evolution across all plant taxa. Future research should expand the dataset to encompass a broader range of species with varying rubber production capabilities and actively explore additional functional roles of *REF* genes in plants, ultimately aiming to diversify potential sources of natural rubber. Overall, this work enhances the understanding of *REF* gene evolution and function in plants, providing a theoretical foundation for identifying species with the potential for rubber production and for breeding high-yielding varieties.

## Data Availability

The data presented in this study are available in this article and the Supplementary Materials.

## Supplementary Materials

The following supporting information can be downloaded at https://www.mdpi.com/article/10.3390/cimb46110701/s1: Figure S1: Structural analysis of 87 *REF* genes. Figure S2: Metabolic pathways of REF proteins. Figure S3: Multiple sequence alignments of identified REF proteins. Table S1: Incorrect *REF* site information from genome annotation files of 17 plant species, along with amino acid sequence information of 87 REF proteins. Table S2: The 17 plant species involved in this study. Table S3: Information on downloaded reference genomes and transcriptome data of 17 plant species. Table S4: Analysis results of physicochemical properties of REF proteins. Table S5: Expression levels of *REF* genes measured in FPKM values. Table S6: Subcellular localization of the *REF* gene. File S1: Predicted tertiary structures of 87 REF proteins. File S2: Chromosomal localization of *REF* genes in 17 plant species. File S3: Collinearity analysis of *H. brasiliensis* and other plants.

## References

[1] Venkatachalam P., Geetha N., Sangeetha P., Thulaseedharan A. (2013). Natural rubber producing plants: An overview. Afr. J. Biotechnol..

[2] Pareed A.O., Kumaran M.P. (2017). Price volatility and its impact on rubber cultivation in India–An analysis of recent trends. J. Acad. Res. Econ..

[3] Nicod T., Bathfield B., Bosc P.-M., Promkhambut A., Duangta K., Chambon B. (2020). Households’ livelihood strategies facing market uncertainties: How did Thai farmers adapt to a rubber price drop?. Agric. Syst..

[4] Neadkhun P., Borisutdhi Y., Simarak S., Panpakdee C. (2023). Coping strategies of rubber farmers in Bueng Kan, Thailand during a period of price fluctuations. J. Arts Soc. Sci. Stud..

[5] Cornish K. (2001). Similarities and differences in rubber biochemistry among plant species. Phytochemistry.

[6] Baboo M., Dixit M., Sharma K., Saxena N. (2010). Activation energy and thermo-mechanical properties of trans-polyisoprene and liquid cis-polyisoprene blends. Thermochim. Acta.

[7] Boochathum P., Prajudtake W. (2001). Vulcanization of cis-and trans-polyisoprene and their blends: Cure characteristics and crosslink distribution. Eur. Polym. J..

[8] Bushman B.S., Scholte A.A., Cornish K., Scott D.J., Brichta J.L., Vederas J.C., Ochoa O., Michelmore R.W., Shintani D.K., Knapp S.J. (2006). Identification and comparison of natural rubber from two Lactuca species. Phytochemistry.

[9] Lloyd F.E. (1911). Guayule (Parthenium Argentatum Gray): A Rubber-Plant of the Chihuahuan Desert.

[10] Azahar N., Hassan N., Jaya R.P., Kadir M., Yunus N., Mahmud M.Z.H. (2016). An overview on natural rubber application for asphalt modification. Int. J. Agric..

[11] Nakazawa Y., Bamba T., Takeda T., Uefuji H., Harada Y., Li X., Chen R., Inoue S., Tutumi M., Shimizu T. (2009). Production of Eucommia-rubber from *Eucommia ulmoides* Oliv. (hardy rubber tree). J. Plant Biol..

[12] Cornish K. (2017). Alternative natural rubber crops: Why should we care?. Technol. Innov..

[13] Hagel J.M., Yeung E.C., Facchini P.J. (2008). Got milk? The secret life of laticifers. Trends Plant Sci..

[14] Rivano F., Vera J., Cevallos V., Lacote R., Gohet E. (2024). Productivity evaluation of 10 *Hevea brasiliensis* clones in Ecuador under escape conditions for South American leaf blight. Sci. Rep..

[15] Tian M., Li W., Luo P., He J., Zhang H., Yan Q., Ye Y.J.S.R. (2024). Genetic diversity analysis and core germplasm bank construction in cold resistant germplasm of rubber trees (*Hevea brasiliensis*). Sci. Rep..

[16] Jara F.M., García-Martínez M.d.l.M., López-Córcoles H., Carrión M.E., Zalacain A., Carmona M.J.P. (2024). Evaluating Guayule (*Parthenium argentatum* A. Gray) Germplasm Grown in Spain: Rubber and Resin along Three Production Cycles. J. Plant Res..

[17] Liu S., Chen Y., Han D., Tian X., Ma D., Jie X., Zhang J. (2024). Extraction process and characterization of *Taraxacum kok-saghyz* (TKS) latex. Heliyon.

[18] Ning Y., Yang D.-D., Yu X.-C., Cao X. (2023). Multi-omics-driven development of alternative crops for natural rubber production. J. Integr. Agric..

[19] Chen Z., Dong Q., Wang X., Hu S., Yin D., Liu L., Zhang J., Zhao X. (2024). Bio-based *Eucommia ulmoides* gum composites for high-performance engineering tire applications. Ind. Crops Prod..

[20] Dai L., Yang H., Zhao X., Wang L. (2021). Identification of cis conformation natural rubber and proteins in *Ficus altissima* Blume latex. Plant Physiol. Biochem..

[21] Van Beilen J.B., Poirier Y. (2007). Guayule and Russian dandelion as alternative sources of natural rubber. Crit. Rev. Biotechnol..

[22] Mooibroek H., Cornish K. (2000). Alternative sources of natural rubber. Appl. Microbiol. Biotechnol..

[23] Lau N.-S., Makita Y., Kawashima M., Taylor T.D., Kondo S., Othman A.S., Shu-Chien A.C., Matsui M. (2016). The rubber tree genome shows expansion of gene family associated with rubber biosynthesis. Sci. Rep..

[24] Uthup T.K., Rajamani A., Ravindran M., Saha T. (2019). Distinguishing *CPT* gene family members and vetting the sequence structure of a putative rubber synthesizing variant in *Hevea brasiliensis*. Gene.

[25] Asawatreratanakul K., Zhang Y.W., Wititsuwannakul D., Wititsuwannakul R., Takahashi S., Rattanapittayaporn A., Koyama T. (2003). Molecular cloning, expression and characterization of cDNA encoding cis-prenyltransferases from Hevea brasiliensis: A key factor participating in natural rubber biosynthesis. Eur. J. Biochem..

[26] Yokota S., Suzuki Y., Saitoh K., Kitajima S., Ohya N., Gotoh T. (2018). Cloning and Aggregation Characterization of Rubber Elongation Factor and Small Rubber Particle Protein from *Ficus carica*. Mol. Biotechnol..

[27] Sando T., Hayashi T., Takeda T., Akiyama Y., Nakazawa Y., Fukusaki E., Kobayashi A. (2009). Histochemical study of detailed laticifer structure and rubber biosynthesis-related protein localization in *Hevea brasiliensis* using spectral confocal laser scanning microscopy. Planta.

[28] Berthelot K., Lecomte S., Estevez Y., Coulary-Salin B., Peruch F. (2014). Homologous *Hevea brasiliensis* REF (Hevb1) and SRPP (Hevb3) present different auto-assembling. Biochim. Biophys. Acta (BBA)-Proteins Proteom..

[29] Yamashita S., Yamaguchi H., Waki T., Aoki Y., Mizuno M., Yanbe F., Ishii T., Funaki A., Tozawa Y., Miyagi-Inoue Y. (2016). Identification and reconstitution of the rubber biosynthetic machinery on rubber particles from Hevea brasiliensis. eLife.

[30] Brown D., Feeney M., Ahmadi M., Lonoce C., Sajari R., Di Cola A., Frigerio L. (2017). Subcellular localization and interactions among rubber particle proteins from Hevea brasiliensis. J. Exp. Bot..

[31] Wadeesirisak K., Castano S., Berthelot K., Vaysse L., Bonfils F., Peruch F., Rattanaporn K., Liengprayoon S., Lecomte S., Bottier C. (2017). Rubber particle proteins REF1 and SRPP1 interact differently with native lipids extracted from Hevea brasiliensis latex. Biochim. Biophys. Acta-Biomembr..

[32] Fang Y., Xiao X., Lin J., Lin Q., Wang J., Liu K., Li Z., Xing J., Liu Z., Wang B.J.N.C. (2024). Pan-genome and phylogenomic analyses highlight *Hevea* species delineation and rubber trait evolution. Nat. Commun..

[33] Altschul S.F., Madden T.L., Schäffer A.A., Zhang J., Zhang Z., Miller W., Lipman D.J. (1997). Gapped BLAST and PSI-BLAST: A new generation of protein database search programs. Nucleic Acids Res..

[34] Prakash A., Jeffryes M., Bateman A., Finn R.D. (2017). The HMMER web server for protein sequence similarity search. Curr. Protoc. Bioinform..

[35] Chen C., Wu Y., Li J., Wang X., Zeng Z., Xu J., Liu Y., Feng J., Chen H., He Y. (2023). TBtools-II: A “one for all, all for one” bioinformatics platform for biological big-data mining. Mol. Plant.

[36] Edgar R.C. (2004). MUSCLE: Multiple sequence alignment with high accuracy and high throughput. Nucleic Acids Res..

[37] Waterhouse A.M., Procter J.B., Martin D.M., Clamp M., Barton G.J. (2009). Jalview Version 2—A multiple sequence alignment editor and analysis workbench. Bioinformatics.

[38] Capella-Gutiérrez S., Silla-Martínez J.M., Gabaldón T. (2009). trimAl: A tool for automated alignment trimming in large-scale phylogenetic analyses. Bioinformatics.

[39] Ponting C. (2007). TreeBeST: Tree Building Guided by Species Tree. https://github.com/Ensembl/treebest.

[40] Cramer P. (2021). AlphaFold2 and the future of structural biology. Nat. Struct. Mol. Biol..

[41] Yuan S., Chan H.S., Hu Z. (2017). Using PyMOL as a platform for computational drug design. Wiley Interdiscip. Rev..

[42] Kim D., Langmead B., Salzberg S.L. (2015). HISAT: A fast spliced aligner with low memory requirements. Nat. Methods.

[43] Li H., Handsaker B., Wysoker A., Fennell T., Ruan J., Homer N., Marth G., Abecasis G., Durbin R., 1000 Genome Project Data Processing Subgroup (2009). The sequence alignment/map format and SAMtools. Bioinformatics.

[44] Liao Y., Smyth G.K., Shi W. (2019). The R package Rsubread is easier, faster, cheaper and better for alignment and quantification of RNA sequencing reads. Nucleic Acids Res..

[45] Cheng H., Song X., Hu Y., Wu T., Yang Q., An Z., Feng S., Deng Z. (2023). Chromosome-level wild *Hevea brasiliensis* genome provides new tools for genomic-assisted breeding and valuable loci to elevate rubber yield. Plant Biotechnol. J..

[46] Tsitsekian D., Daras G., Alatzas A., Templalexis D., Hatzopoulos P., Rigas S. (2019). Comprehensive analysis of Lon proteases in plants highlights independent gene duplication events. J. Exp. Bot..

[47] Soltis D.E., Ma H., Frohlich M.W., Soltis P.S., Albert V.A., Oppenheimer D.G., Altman N.S., Depamphilis C., Leebens-Mack J. (2007). The floral genome: An evolutionary history of gene duplication and shifting patterns of gene expression. Trends Plant Sci..

[48] Ding Z., Fu L., Tan D., Sun X., Zhang J. (2020). An integrative transcriptomic and genomic analysis reveals novel insights into the hub genes and regulatory networks associated with rubber synthesis in *H. brasiliensis*. Ind. Crops Prod..

[49] Janies D., DeSalle R. (1999). Development, evolution, and corroboration. Technol. Innov..

[50] Takahashi H., Buchner P., Yoshimoto N., Hawkesford M.J., Shiu S.-H. (2012). Evolutionary relationships and functional diversity of plant sulfate transporters. Front. Plant Sci..

[51] Bandyopadhyay D., Huan J., Liu J., Prins J., Snoeyink J., Wang W., Tropsha A. (2010). Functional neighbors: Inferring relationships between nonhomologous protein families using family-specific packing motifs. IEEE Trans..

[52] Xu L., Feng G., Yang Z., Xu X., Huang L., Yang Q., Zhang X. (2020). Genome-wide *AP2/ERF* gene family analysis reveals the classification, structure, expression profiles and potential function in orchardgrass (*Dactylis glomerata*). Mol. Biol. Rep..

[53] Horn P.J., James C.N., Gidda S.K., Kilaru A., Dyer J.M., Mullen R.T., Ohlrogge J.B., Chapman K.D. (2013). Identification of a new class of lipid droplet-associated proteins in plants. Plant Physiol..

[54] Xia E.-H., Zhang H.-B., Sheng J., Li K., Zhang Q.-J., Kim C., Zhang Y., Liu Y., Zhu T., Li W. (2017). The tea tree genome provides insights into tea flavor and independent evolution of caffeine biosynthesis. Mol. Plant.

[55] Harms M.J., Thornton J.W. (2010). Analyzing protein structure and function using ancestral gene reconstruction. Curr. Opin. Struct. Biol..

[56] Senior A.W., Evans R., Jumper J., Kirkpatrick J., Sifre L., Green T., Qin C., Žídek A., Nelson A.W. (2020). Improved protein structure prediction using potentials from deep learning. Nat. Rev. Genet..

[57] Priya P., Venkatachalam P., Thulaseedharan A. (2007). Differential expression pattern of rubber elongation factor (REF) mRNA transcripts from high and low yielding clones of rubber tree (*Hevea brasiliensis* Muell. Arg.). Plant Cell Rep..

[58] Dennis M.S., Light D. (1989). Rubber elongation factor from *Hevea brasiliensis*: Identification, characterization, and role in rubber biosynthesis. Biol. Chem..

[59] Cherian S., Ryu S.B., Cornish K. (2019). Natural rubber biosynthesis in plants, the rubber transferase complex, and metabolic engineering progress and prospects. Plant Biotechnol. J..

