# Supplementary figures and images for: Genome-Wide Identification and Expression Analysis of the REF Genes in 17 Species (part 2 of 2)
Source: Curr Issues Mol Biol. 2024 Oct 22;46(11):11797–816. doi: 10.3390/cimb46110701 (PMC11592748; doi:10.3390/cimb46110701)

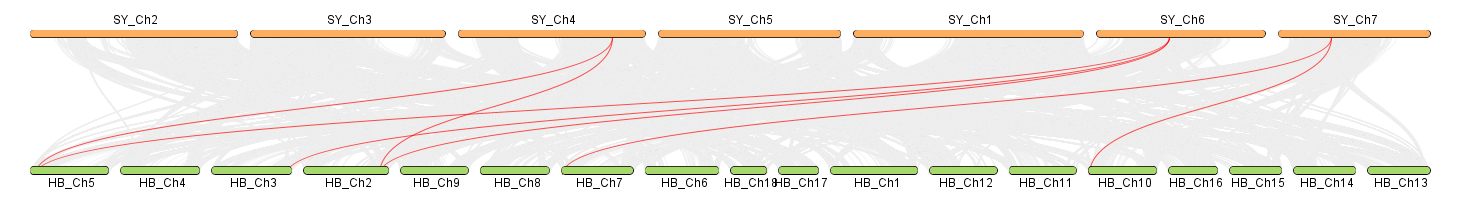

Supplement: Supplementary file 1 [file cimb-46-00701-s001.zip › Supplementary Files/File S3/Speranskia yunnanensis and Hevea brasiliensis.png]
