# Supplementary figures and images for: Genome-Wide Identification and Expression Analysis of the REF Genes in 17 Species (part 1 of 2)
Source: Curr Issues Mol Biol. 2024 Oct 22;46(11):11797–816. doi: 10.3390/cimb46110701 (PMC11592748; doi:10.3390/cimb46110701)

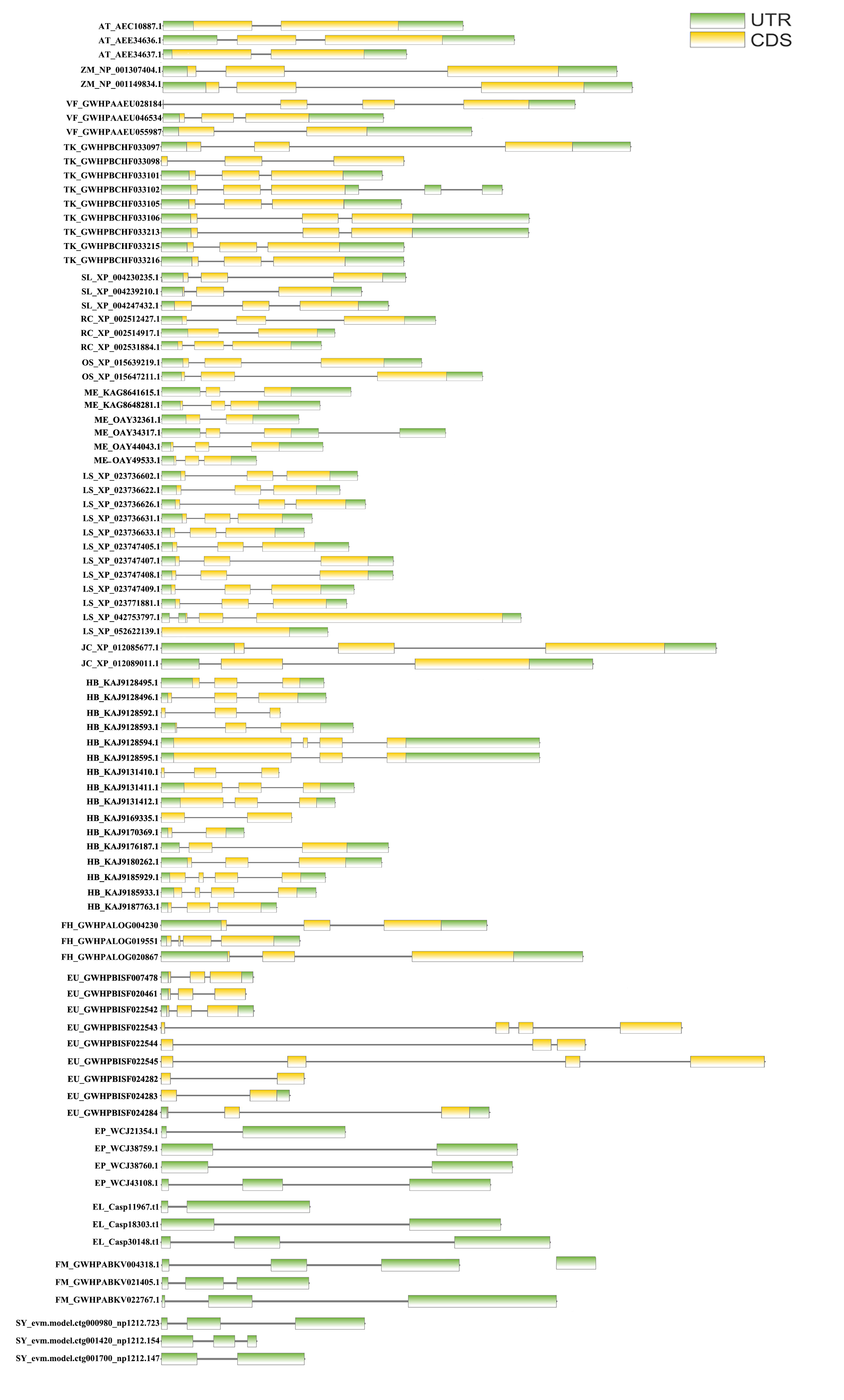

Supplement: Supplementary file 1 [file cimb-46-00701-s001.zip › Supplementary Files/Figure S1.tif]

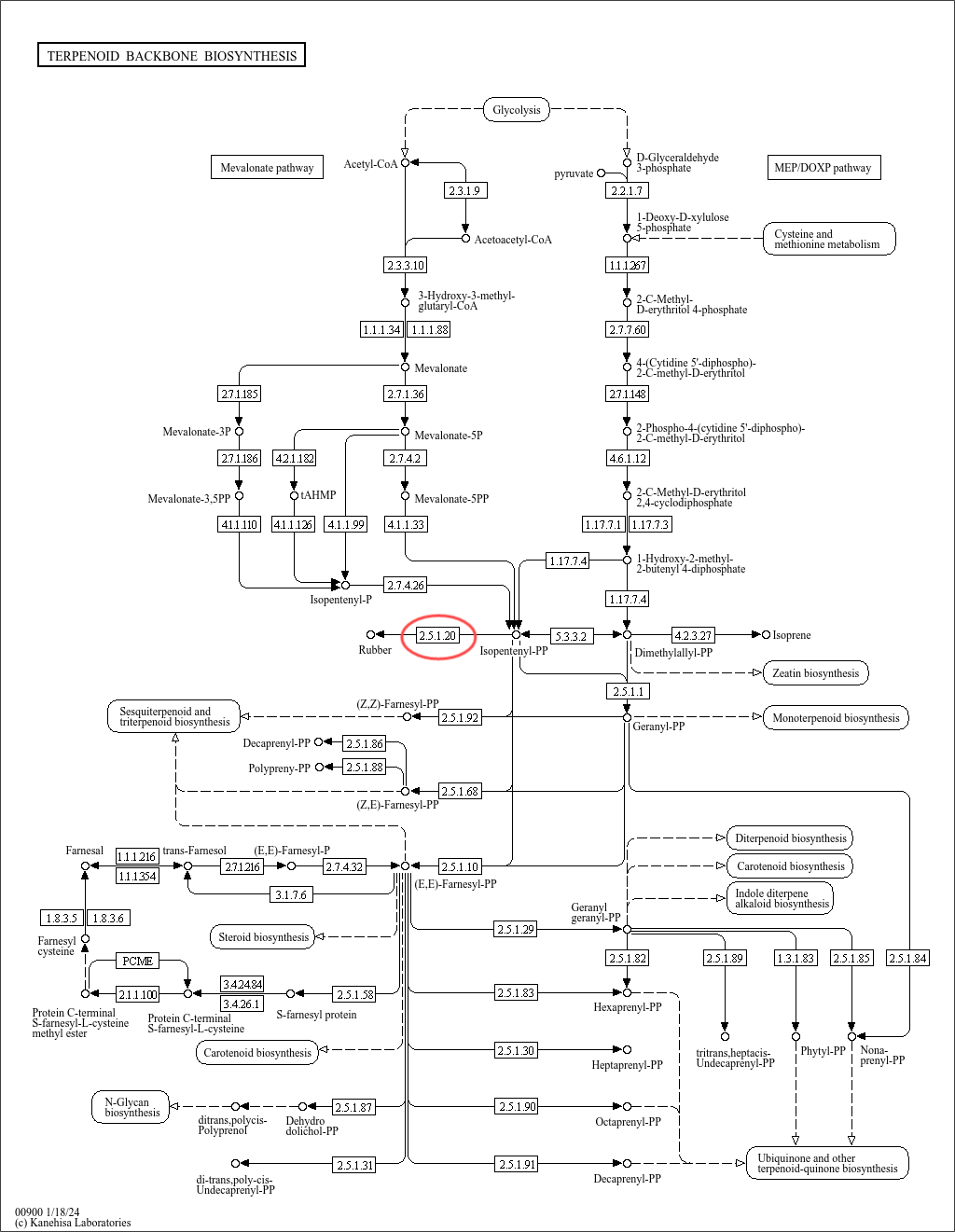

Supplement: Supplementary file 1 [file cimb-46-00701-s001.zip › Supplementary Files/Figure S2.png]

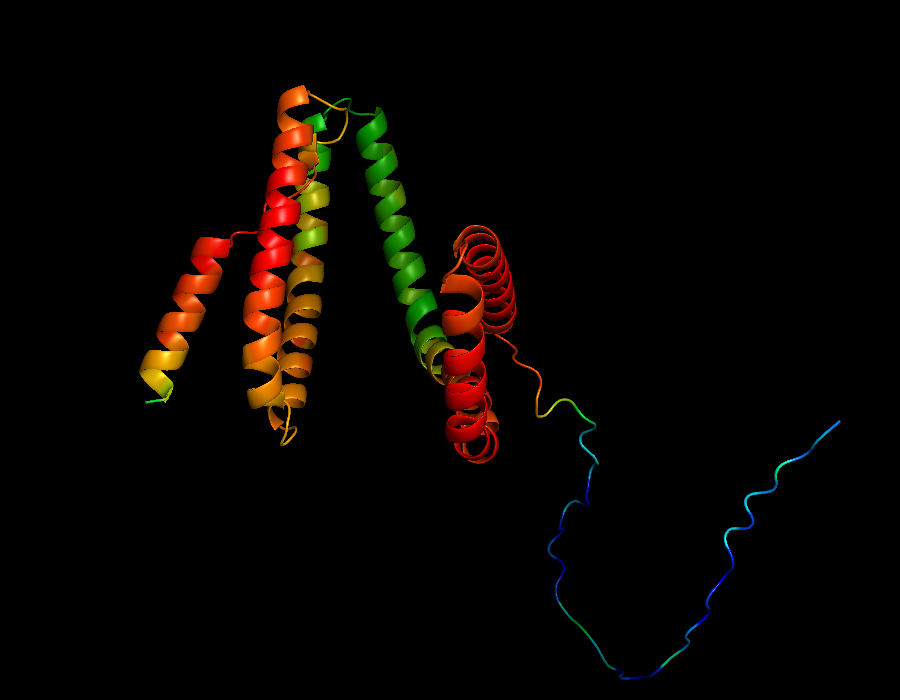

Supplement: Supplementary file 1 [file cimb-46-00701-s001.zip › Supplementary Files/File S1/AT_AEC108871.png]

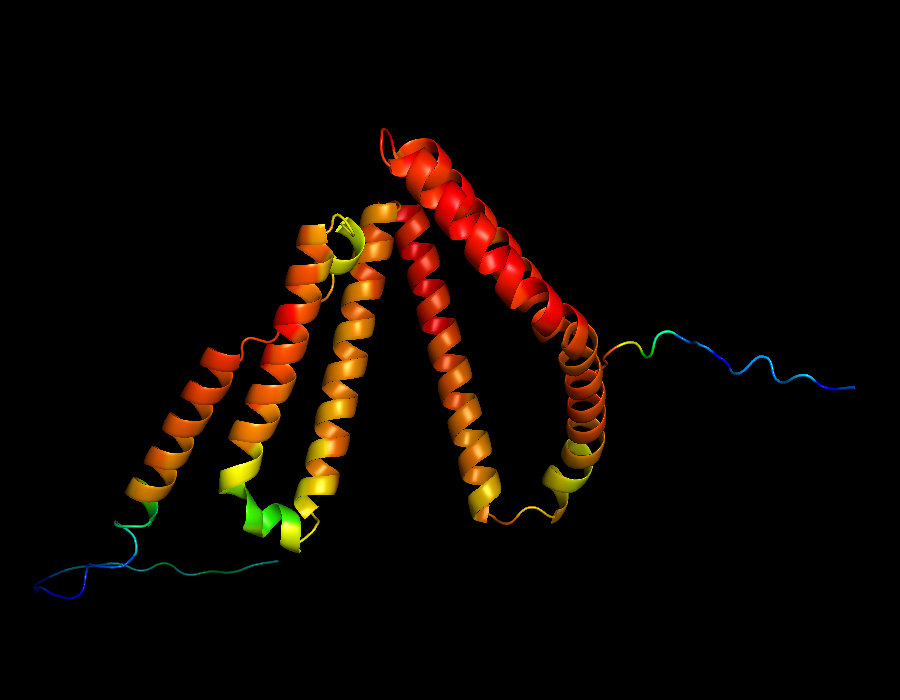

Supplement: Supplementary file 1 [file cimb-46-00701-s001.zip › Supplementary Files/File S1/AT_AEE346361.png]

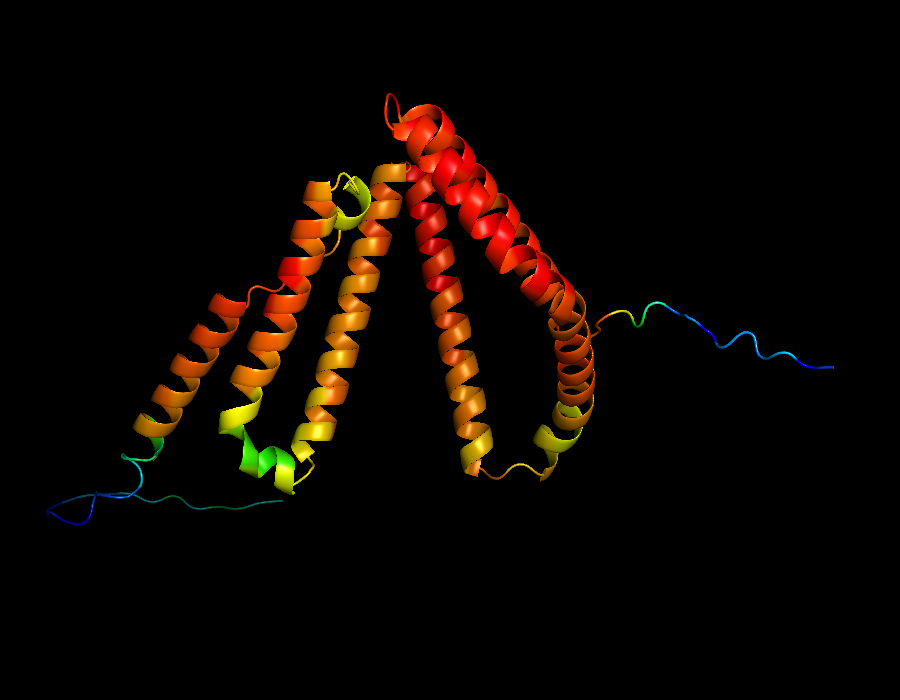

Supplement: Supplementary file 1 [file cimb-46-00701-s001.zip › Supplementary Files/File S1/AT_AEE346371.png]

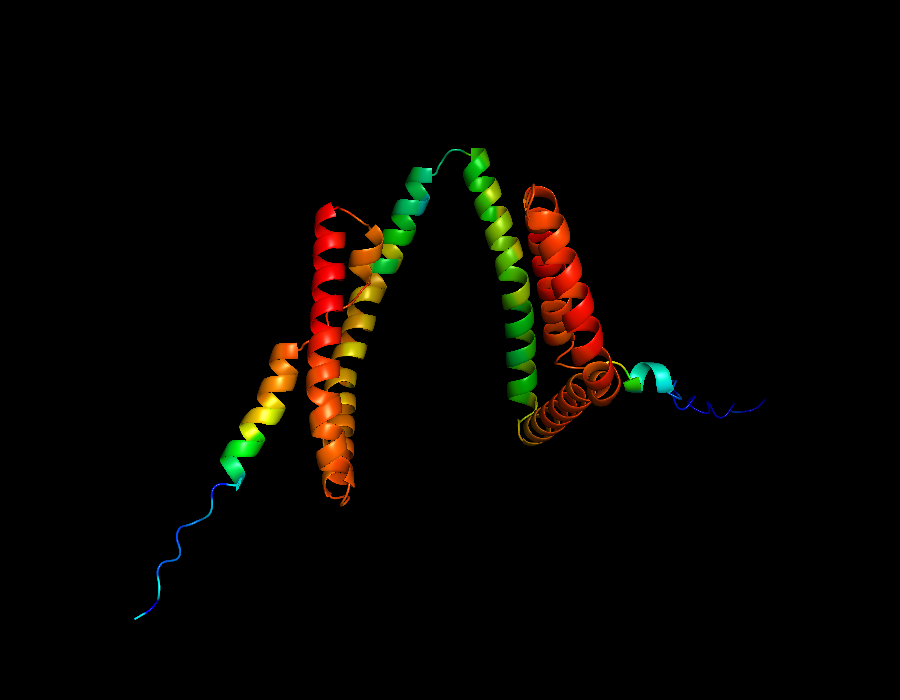

Supplement: Supplementary file 1 [file cimb-46-00701-s001.zip › Supplementary Files/File S1/AT_AEE742491.png]

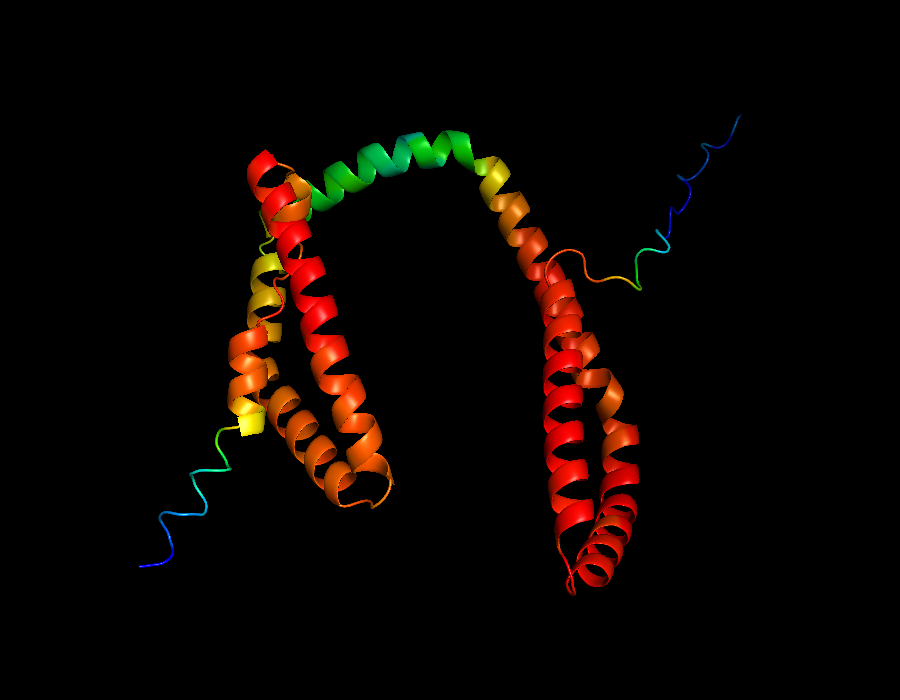

Supplement: Supplementary file 1 [file cimb-46-00701-s001.zip › Supplementary Files/File S1/EL_Casp11967t1.png]

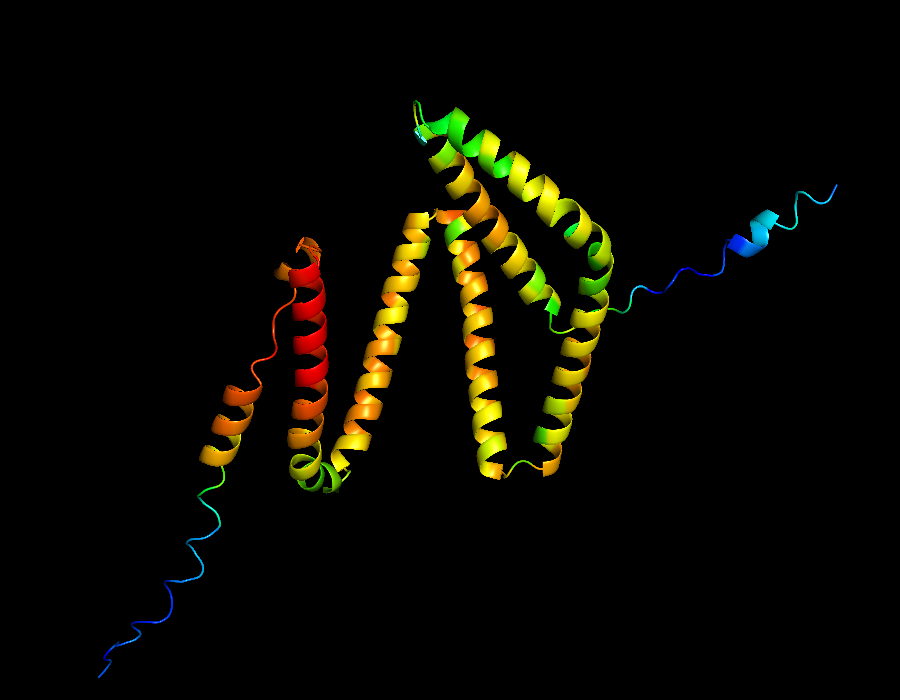

Supplement: Supplementary file 1 [file cimb-46-00701-s001.zip › Supplementary Files/File S1/EL_Casp18303t1.png]

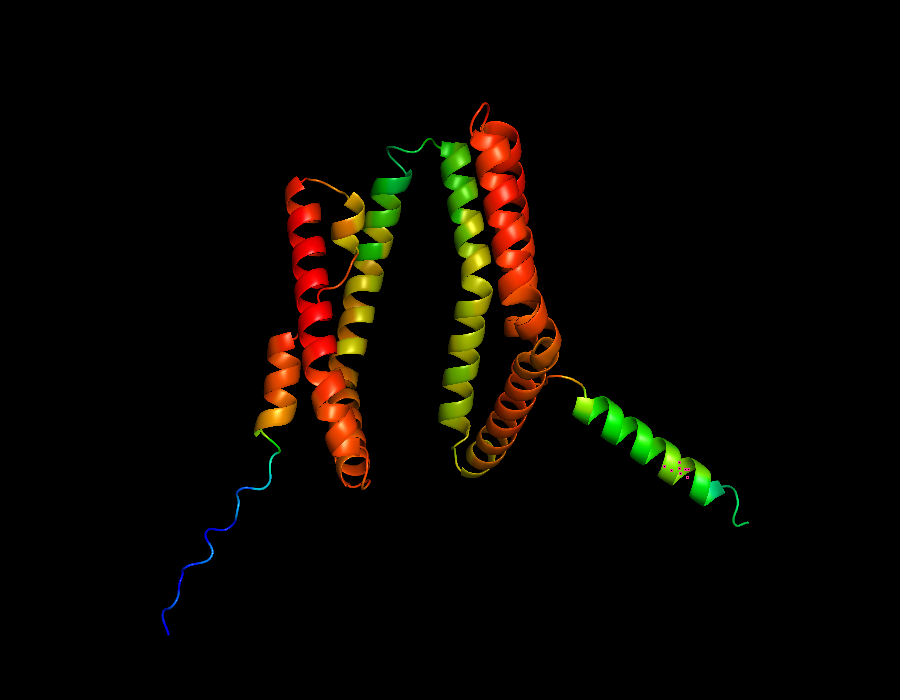

Supplement: Supplementary file 1 [file cimb-46-00701-s001.zip › Supplementary Files/File S1/EL_Casp30148t1.png]

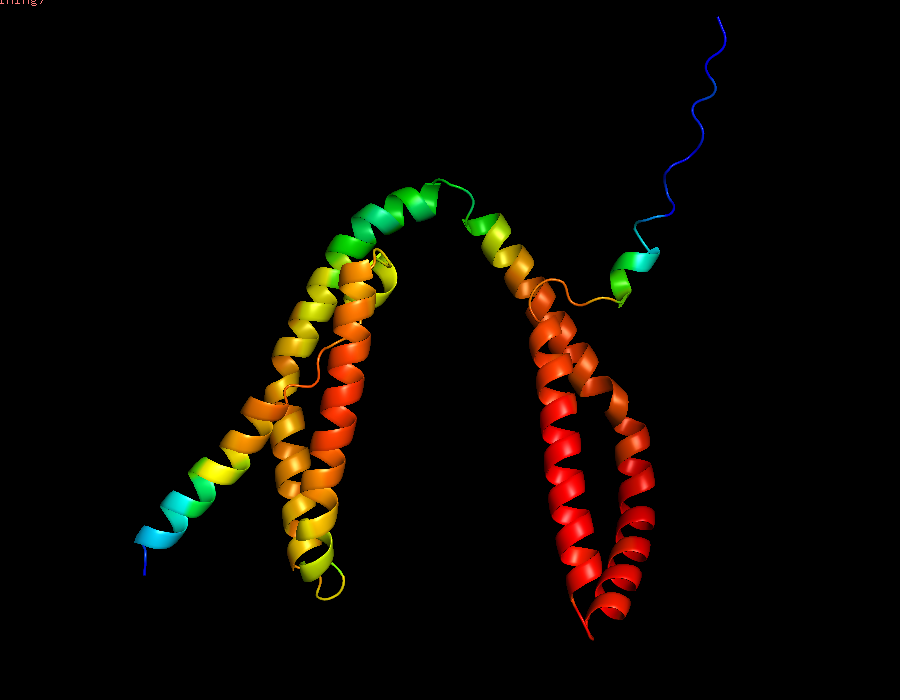

Supplement: Supplementary file 1 [file cimb-46-00701-s001.zip › Supplementary Files/File S1/EP_WCJ213541.png]

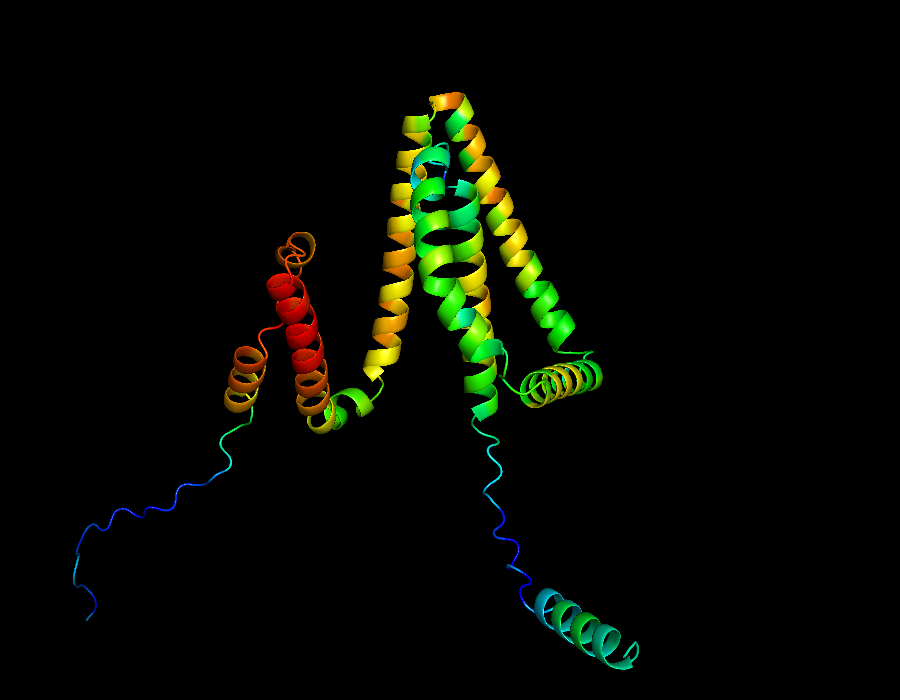

Supplement: Supplementary file 1 [file cimb-46-00701-s001.zip › Supplementary Files/File S1/EP_WCJ387591.png]

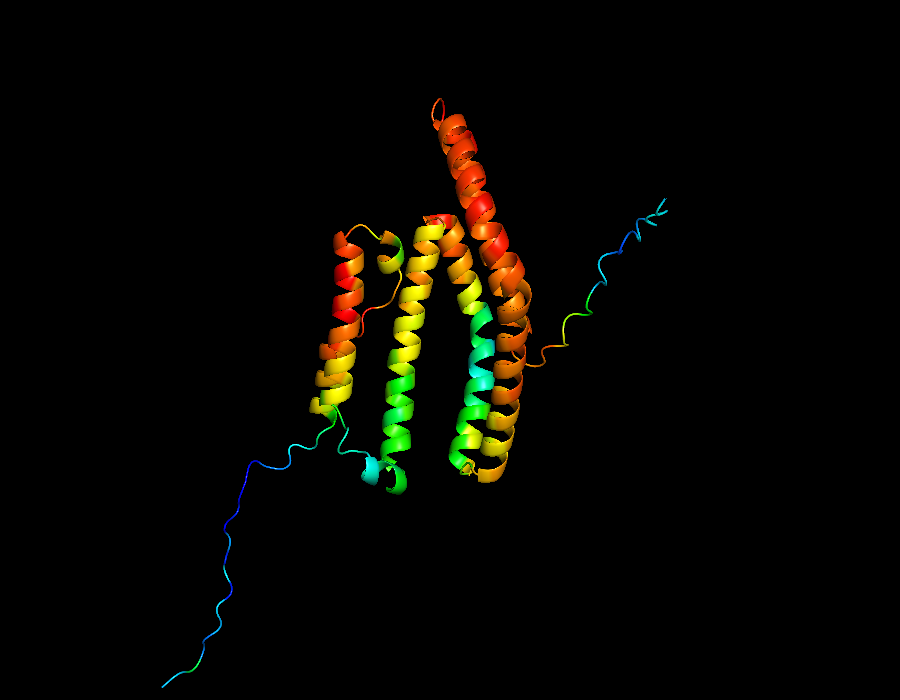

Supplement: Supplementary file 1 [file cimb-46-00701-s001.zip › Supplementary Files/File S1/EP_WCJ387601.png]

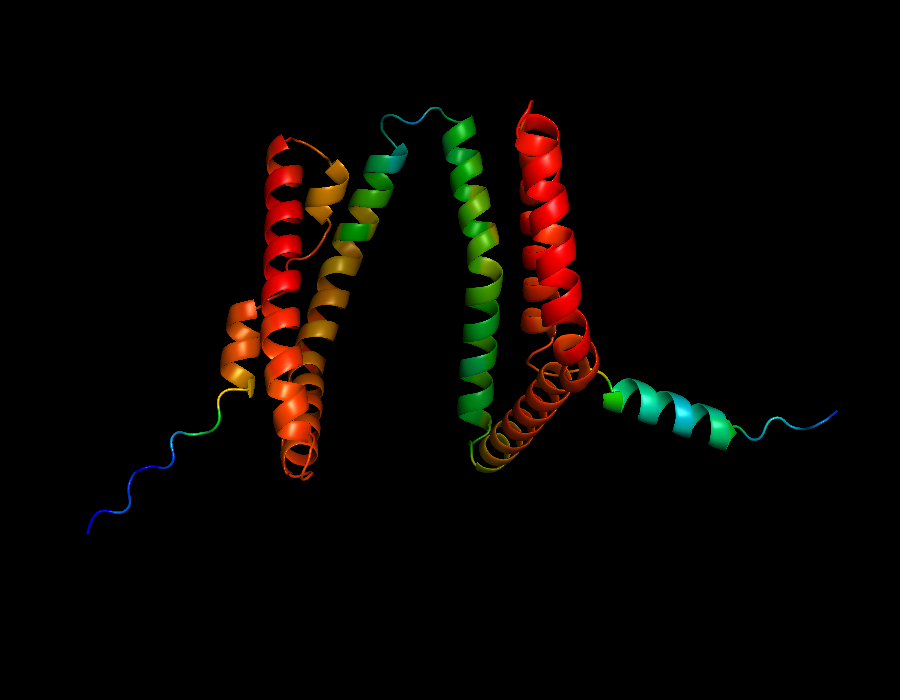

Supplement: Supplementary file 1 [file cimb-46-00701-s001.zip › Supplementary Files/File S1/EP_WCJ431081.png]

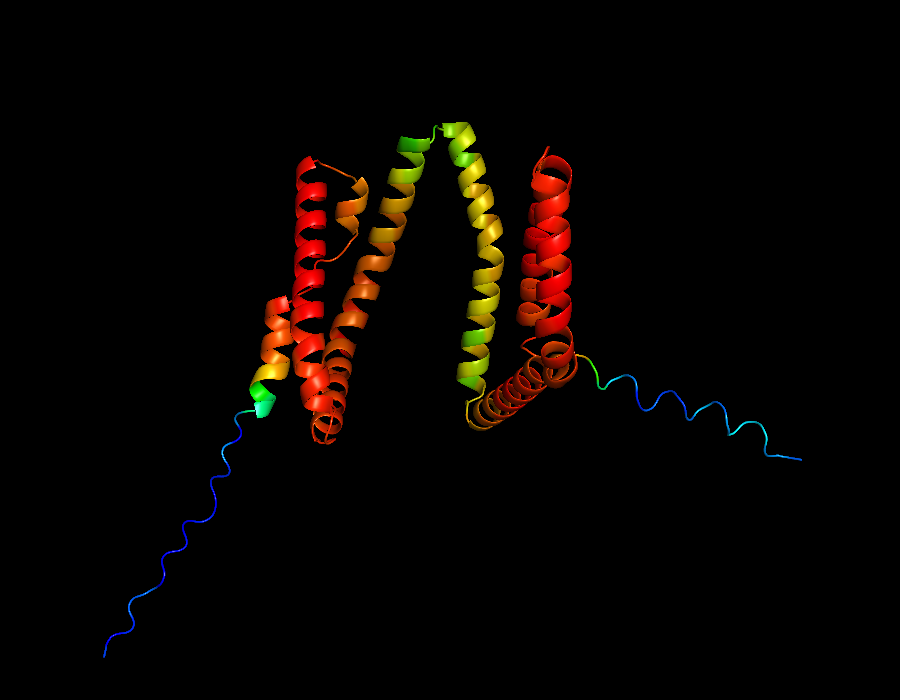

Supplement: Supplementary file 1 [file cimb-46-00701-s001.zip › Supplementary Files/File S1/EU_GWHPBISF007478.png]

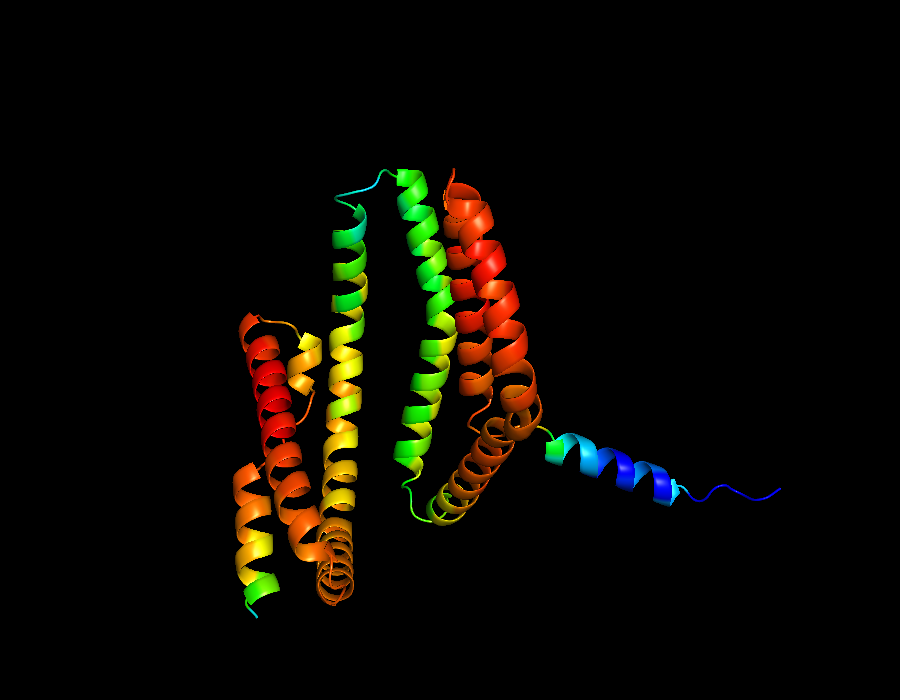

Supplement: Supplementary file 1 [file cimb-46-00701-s001.zip › Supplementary Files/File S1/EU_GWHPBISF020461.png]

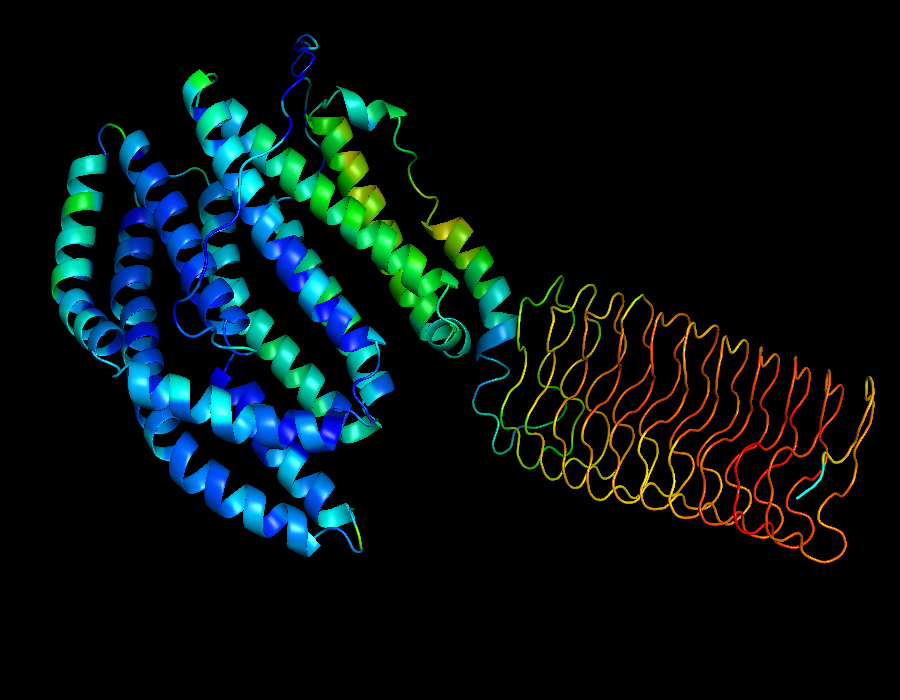

Supplement: Supplementary file 1 [file cimb-46-00701-s001.zip › Supplementary Files/File S1/EU_GWHPBISF022542.png]

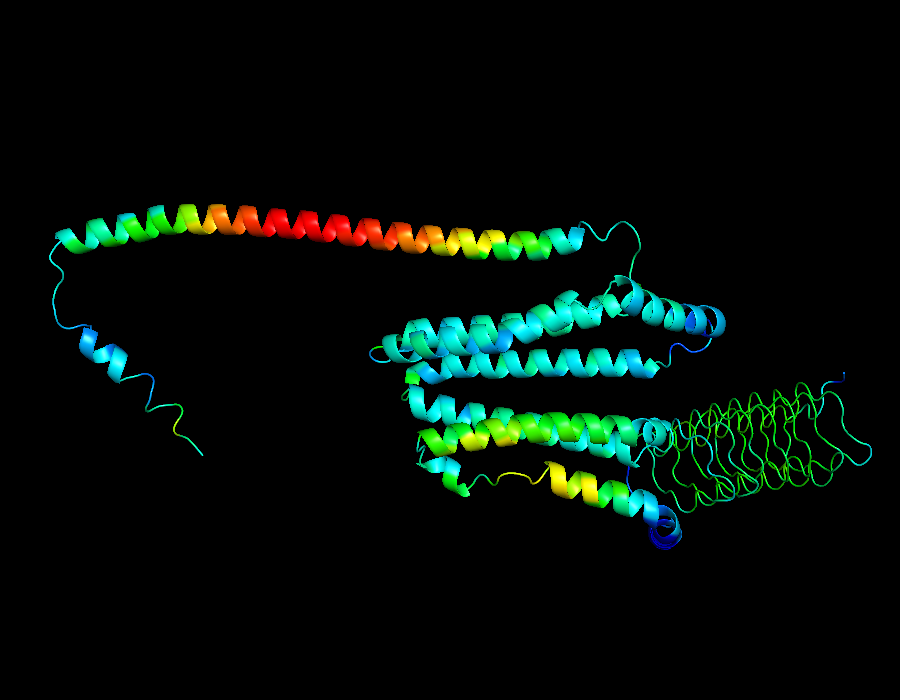

Supplement: Supplementary file 1 [file cimb-46-00701-s001.zip › Supplementary Files/File S1/EU_GWHPBISF022543.png]

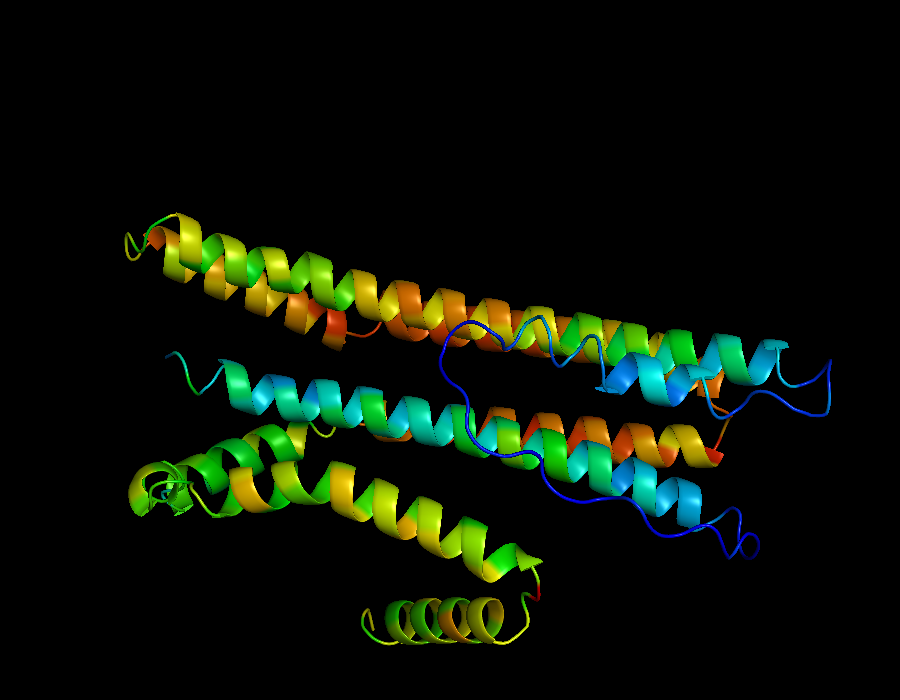

Supplement: Supplementary file 1 [file cimb-46-00701-s001.zip › Supplementary Files/File S1/EU_GWHPBISF022544.png]

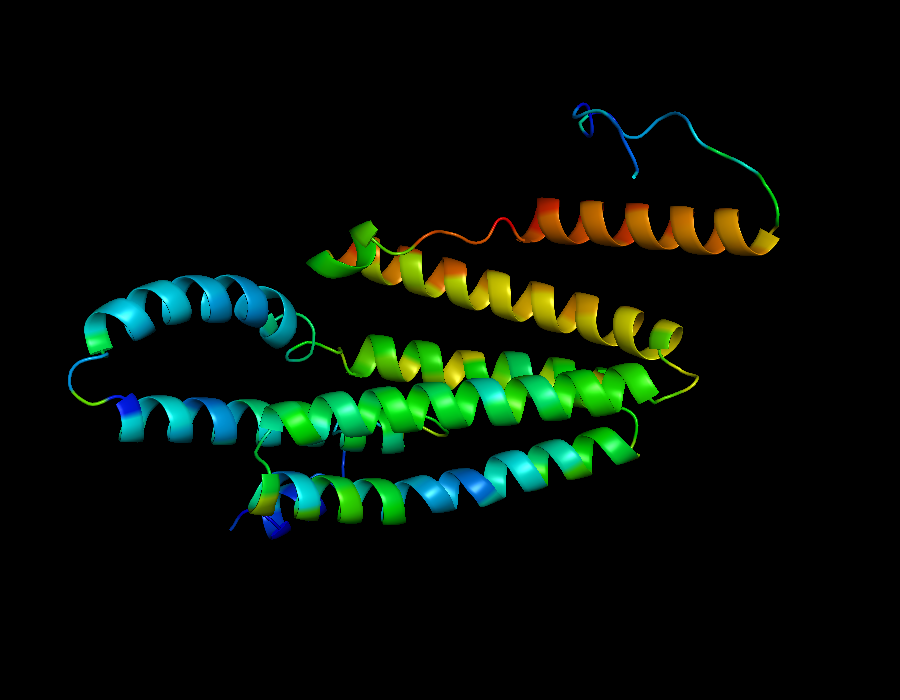

Supplement: Supplementary file 1 [file cimb-46-00701-s001.zip › Supplementary Files/File S1/EU_GWHPBISF022545.png]

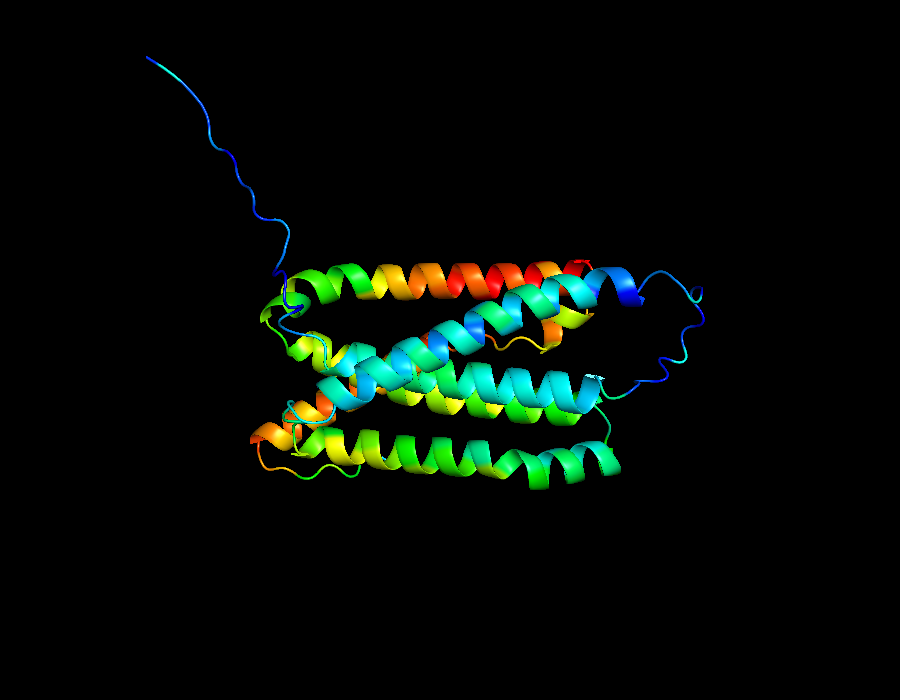

Supplement: Supplementary file 1 [file cimb-46-00701-s001.zip › Supplementary Files/File S1/EU_GWHPBISF024282.png]

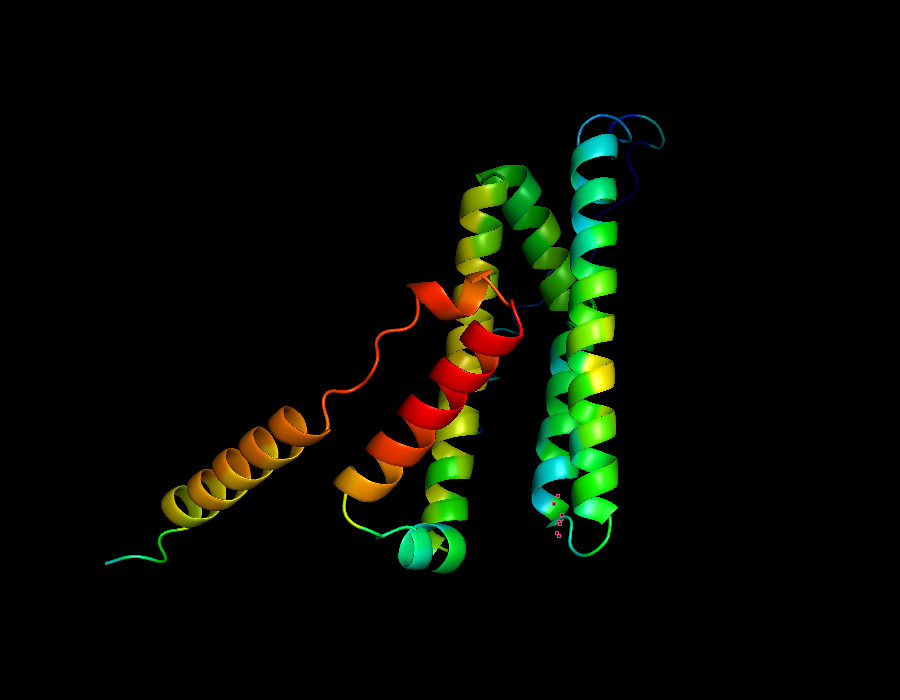

Supplement: Supplementary file 1 [file cimb-46-00701-s001.zip › Supplementary Files/File S1/EU_GWHPBISF024283.png]

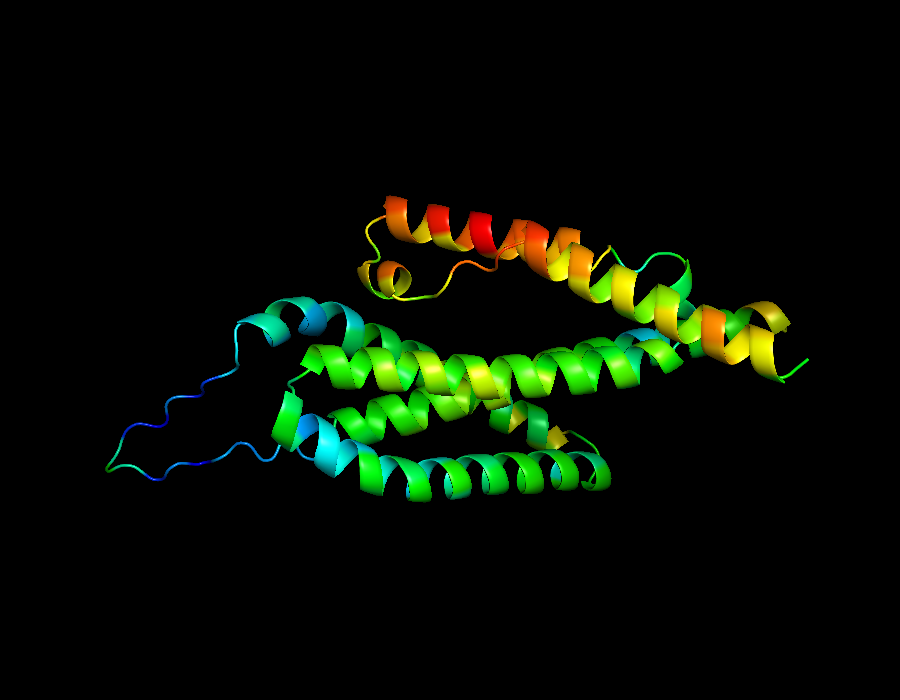

Supplement: Supplementary file 1 [file cimb-46-00701-s001.zip › Supplementary Files/File S1/EU_GWHPBISF024284.png]

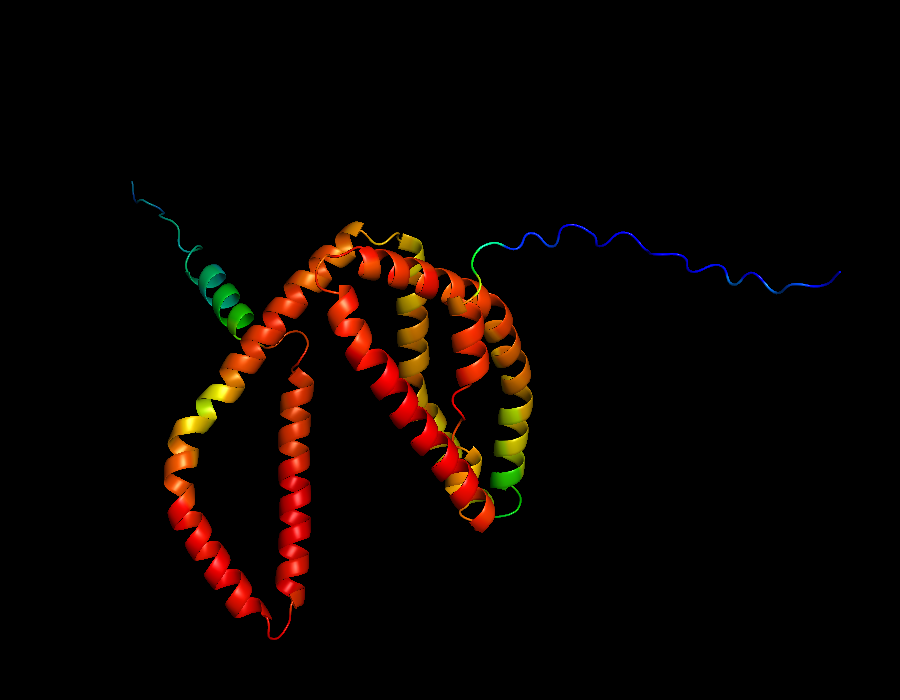

Supplement: Supplementary file 1 [file cimb-46-00701-s001.zip › Supplementary Files/File S1/FH_GWHPALOG004230.png]

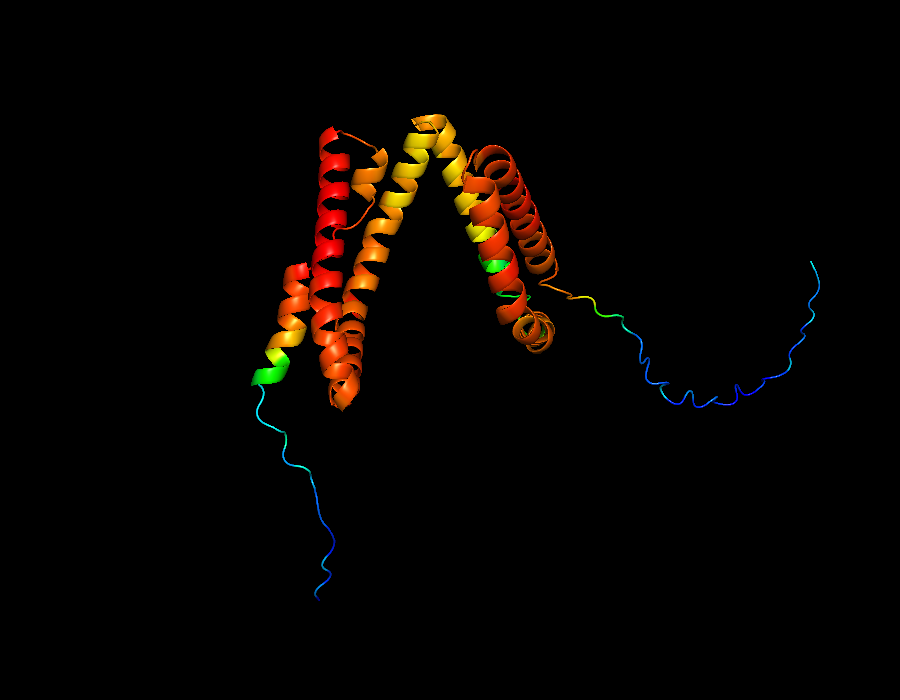

Supplement: Supplementary file 1 [file cimb-46-00701-s001.zip › Supplementary Files/File S1/FH_GWHPALOG019551.png]

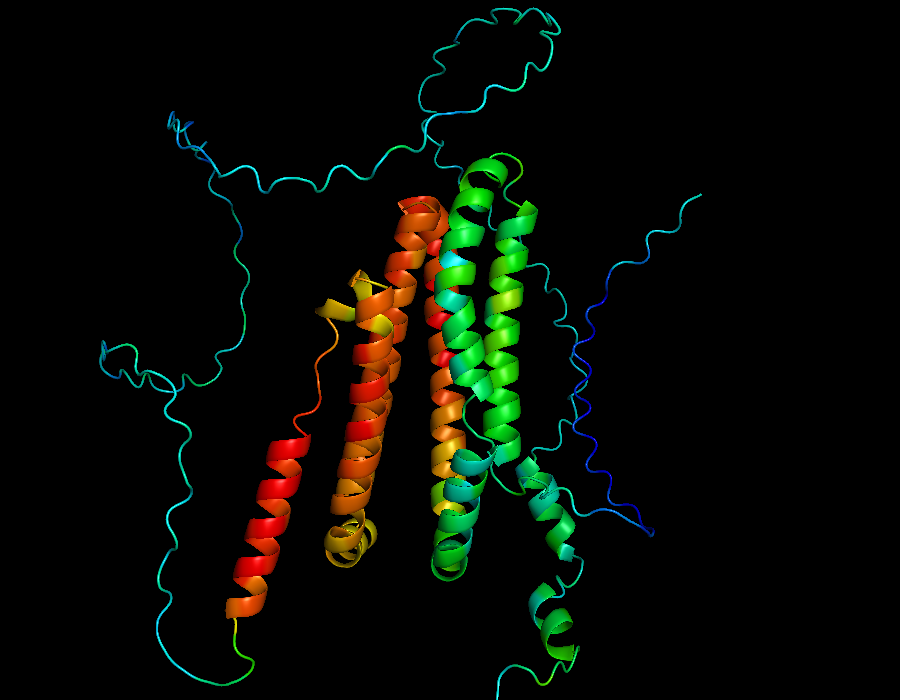

Supplement: Supplementary file 1 [file cimb-46-00701-s001.zip › Supplementary Files/File S1/FH_GWHPALOG020867.png]

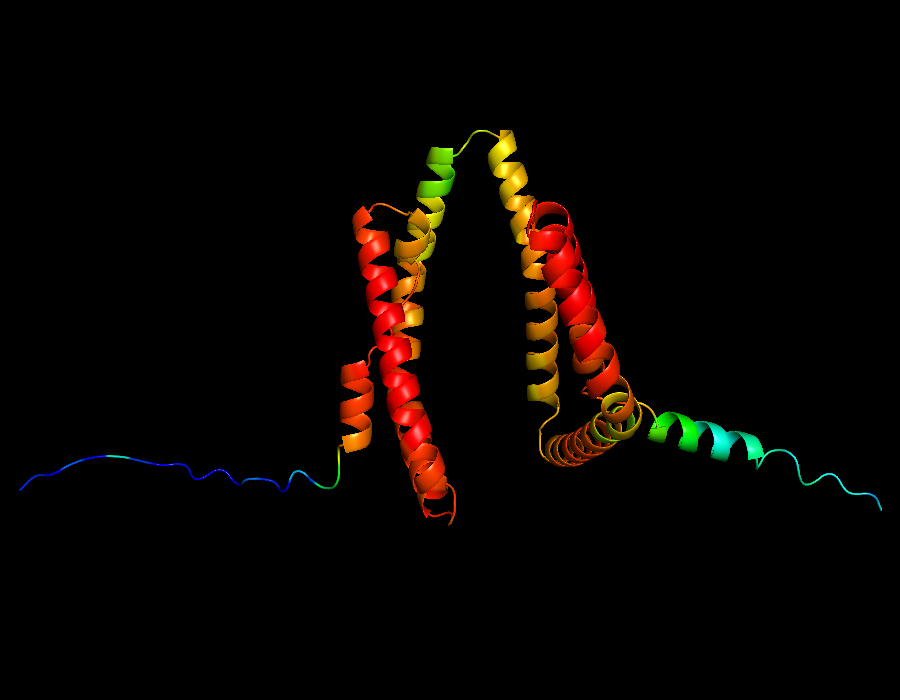

Supplement: Supplementary file 1 [file cimb-46-00701-s001.zip › Supplementary Files/File S1/FM_GWHPABKV0043181.png]

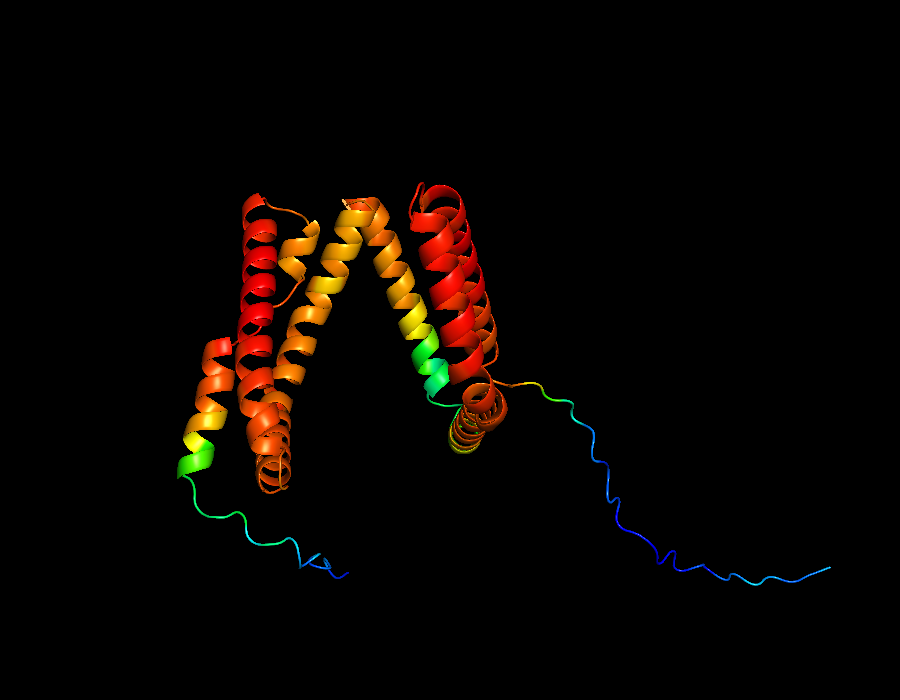

Supplement: Supplementary file 1 [file cimb-46-00701-s001.zip › Supplementary Files/File S1/FM_GWHPABKV0214051.png]

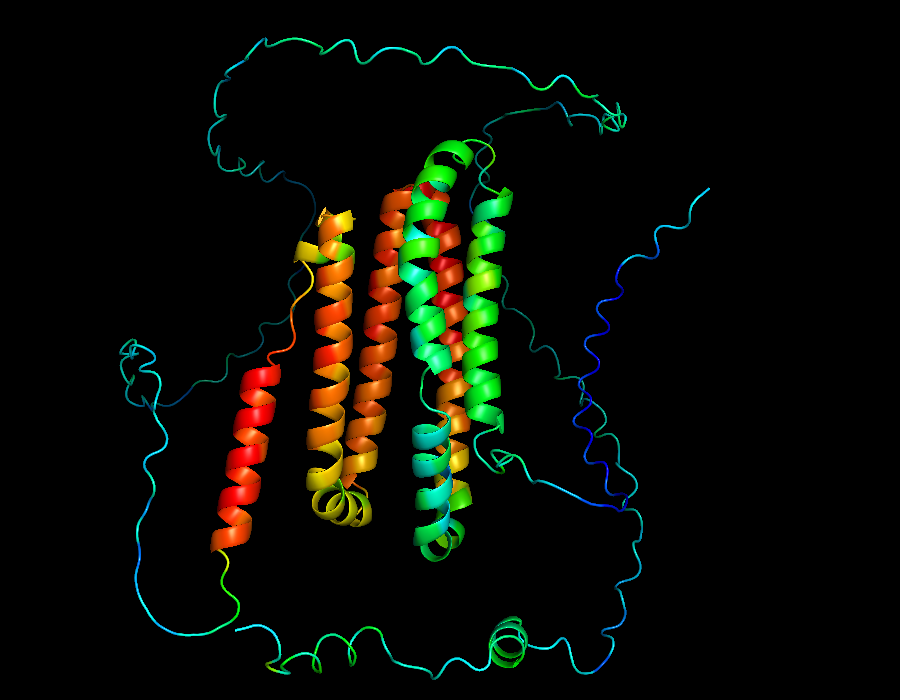

Supplement: Supplementary file 1 [file cimb-46-00701-s001.zip › Supplementary Files/File S1/FM_GWHPABKV0227671.png]

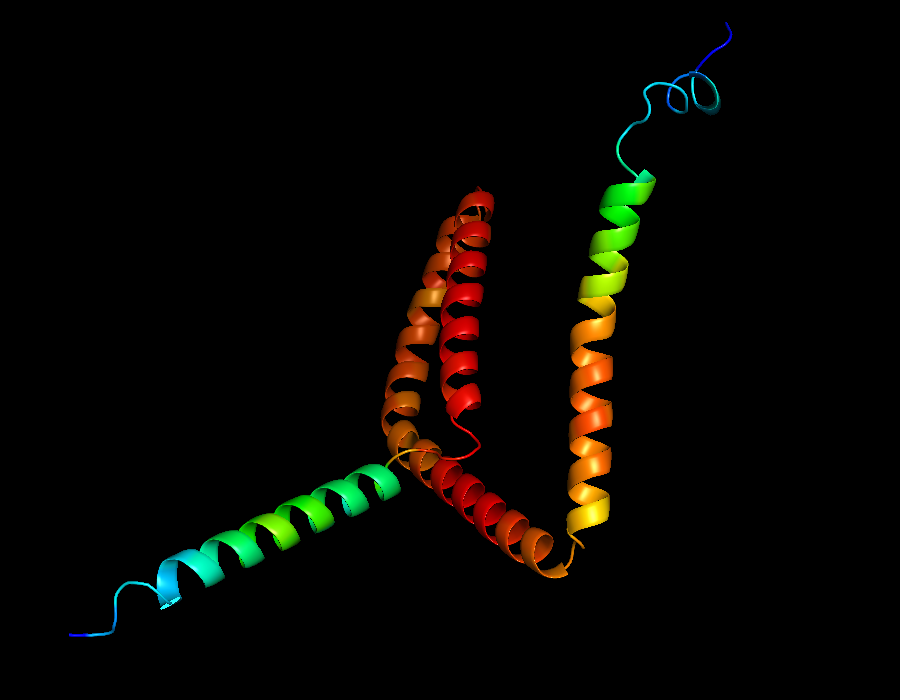

Supplement: Supplementary file 1 [file cimb-46-00701-s001.zip › Supplementary Files/File S1/HB_KAJ91284951.png]

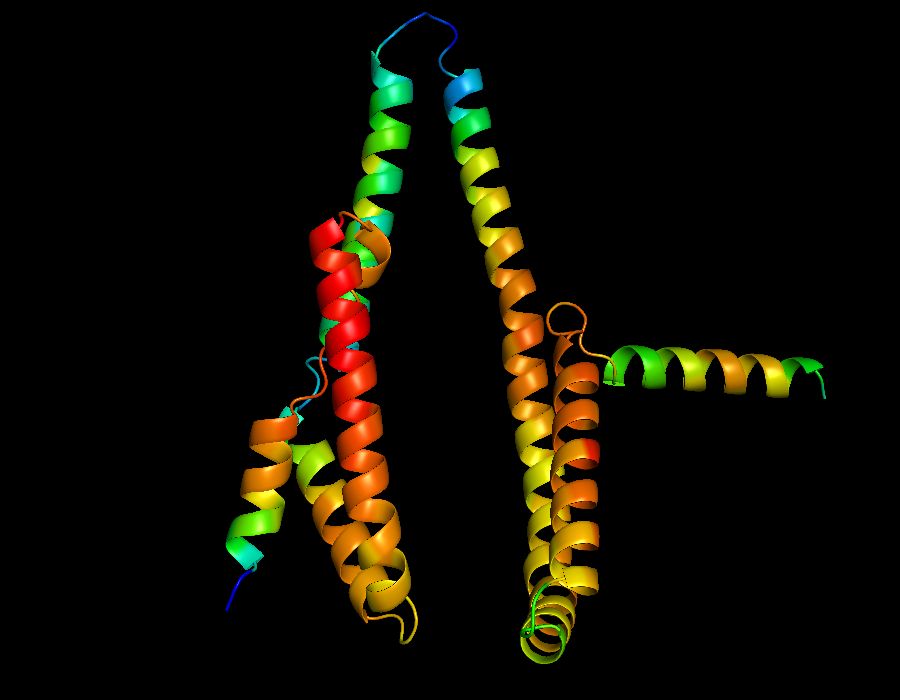

Supplement: Supplementary file 1 [file cimb-46-00701-s001.zip › Supplementary Files/File S1/HB_KAJ91284961.png]

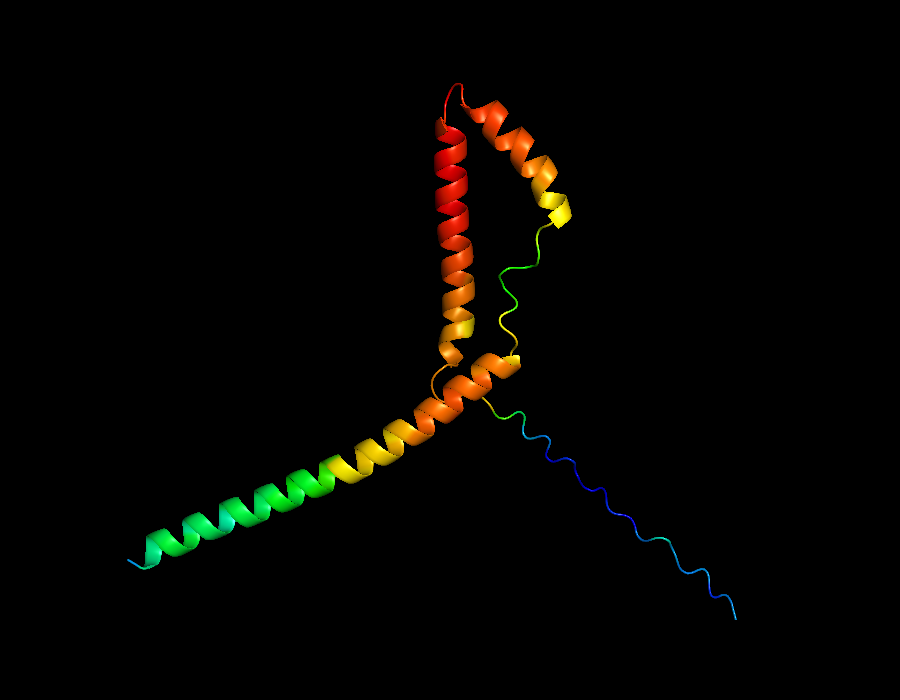

Supplement: Supplementary file 1 [file cimb-46-00701-s001.zip › Supplementary Files/File S1/HB_KAJ91285921.png]

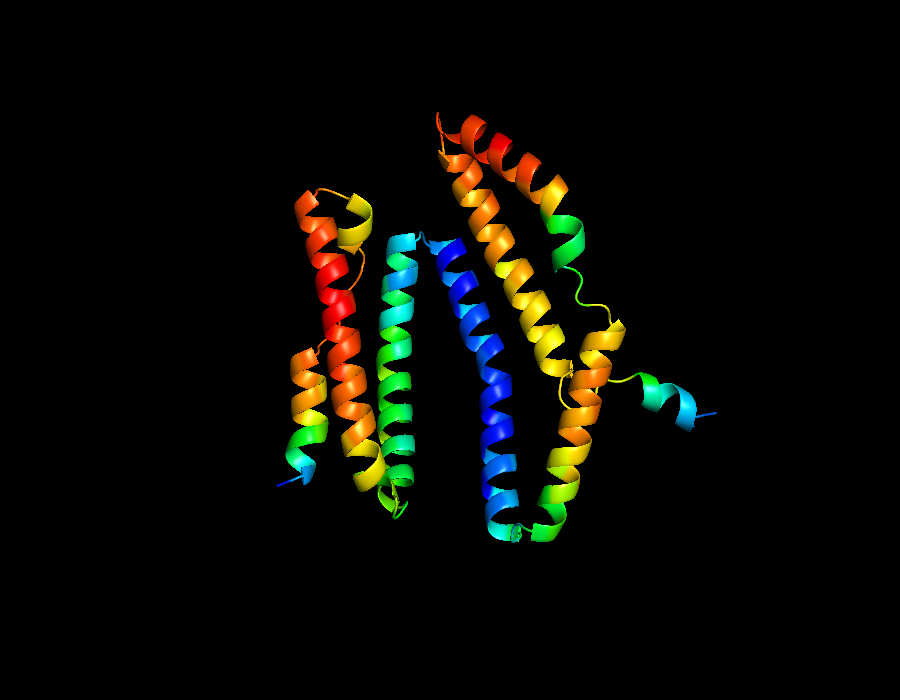

Supplement: Supplementary file 1 [file cimb-46-00701-s001.zip › Supplementary Files/File S1/HB_KAJ91285931.png]

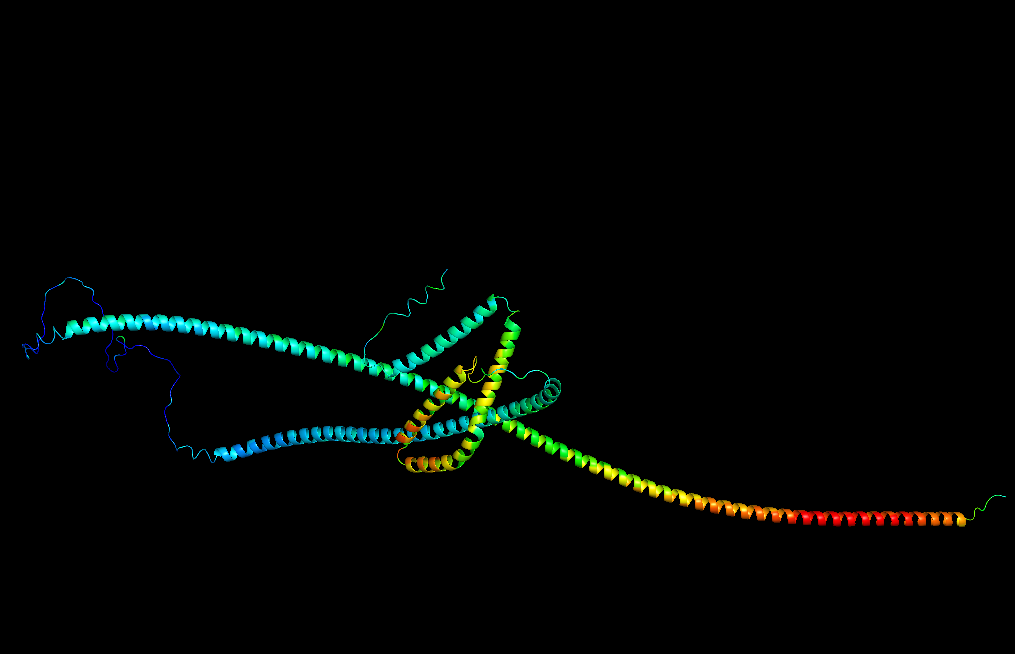

Supplement: Supplementary file 1 [file cimb-46-00701-s001.zip › Supplementary Files/File S1/HB_KAJ91285941.png]

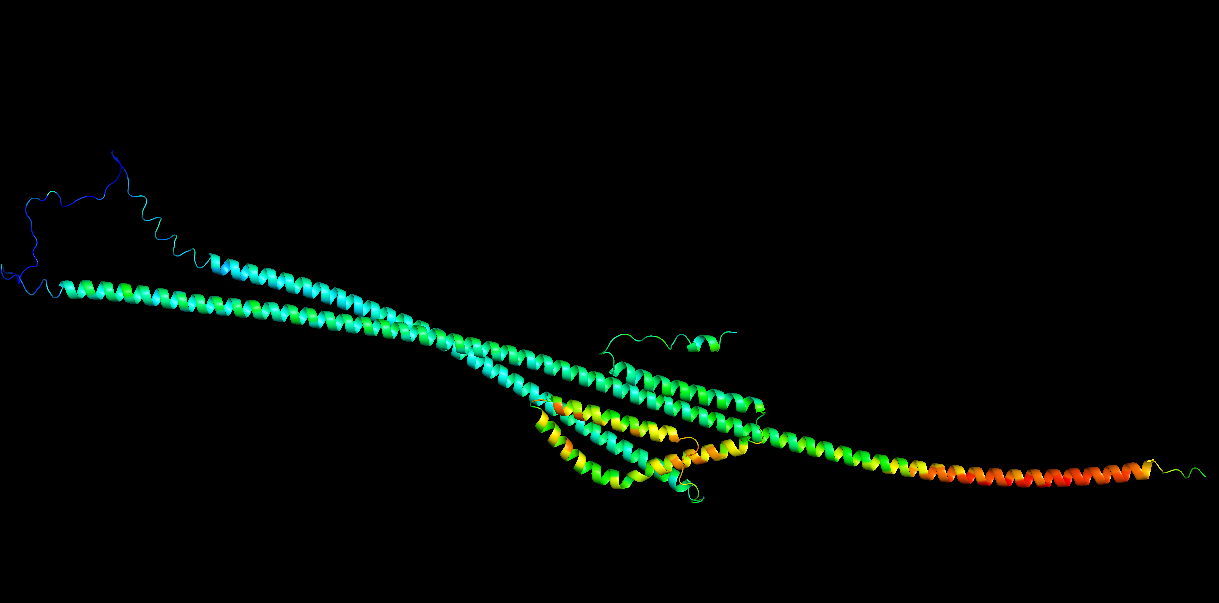

Supplement: Supplementary file 1 [file cimb-46-00701-s001.zip › Supplementary Files/File S1/HB_KAJ91285951.png]

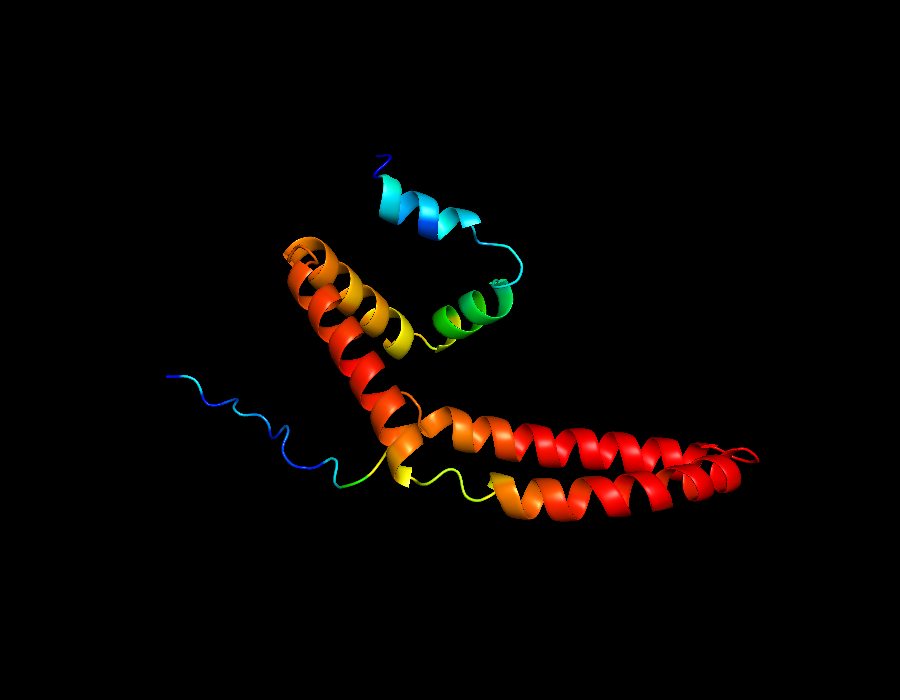

Supplement: Supplementary file 1 [file cimb-46-00701-s001.zip › Supplementary Files/File S1/HB_KAJ91314101.png]

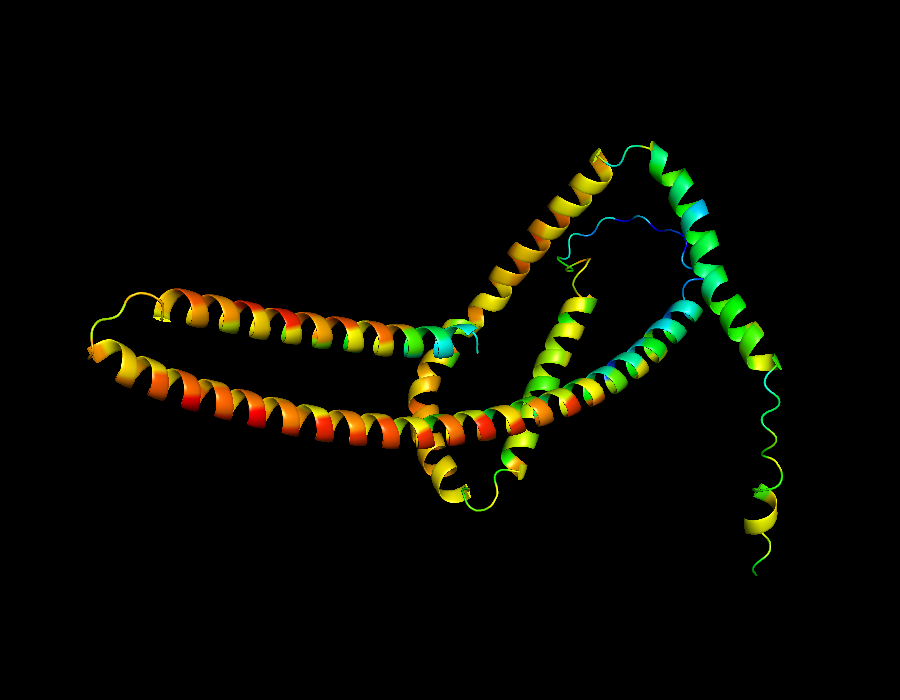

Supplement: Supplementary file 1 [file cimb-46-00701-s001.zip › Supplementary Files/File S1/HB_KAJ91314111.png]

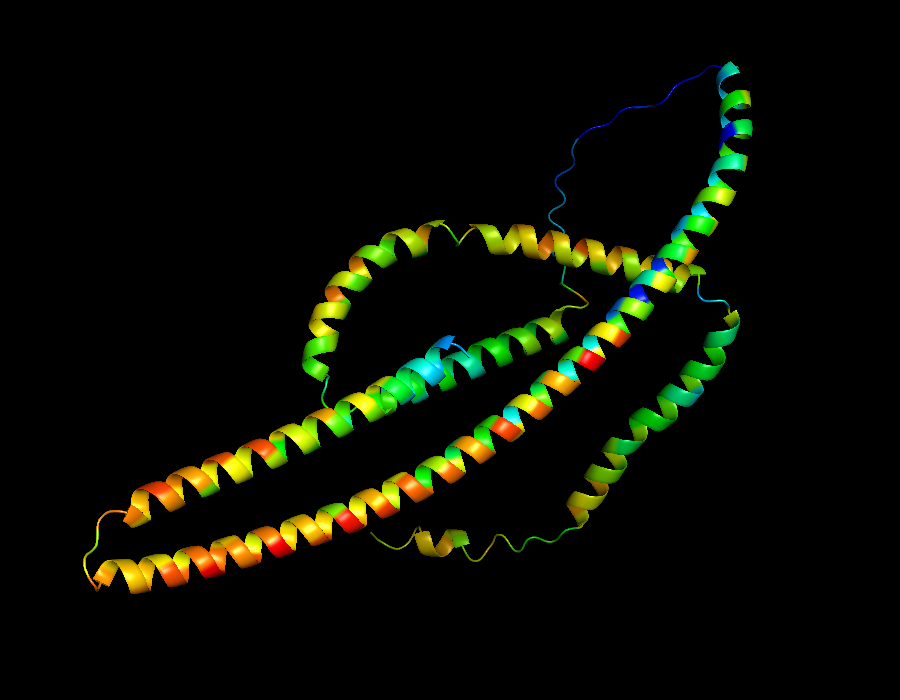

Supplement: Supplementary file 1 [file cimb-46-00701-s001.zip › Supplementary Files/File S1/HB_KAJ91314121.png]

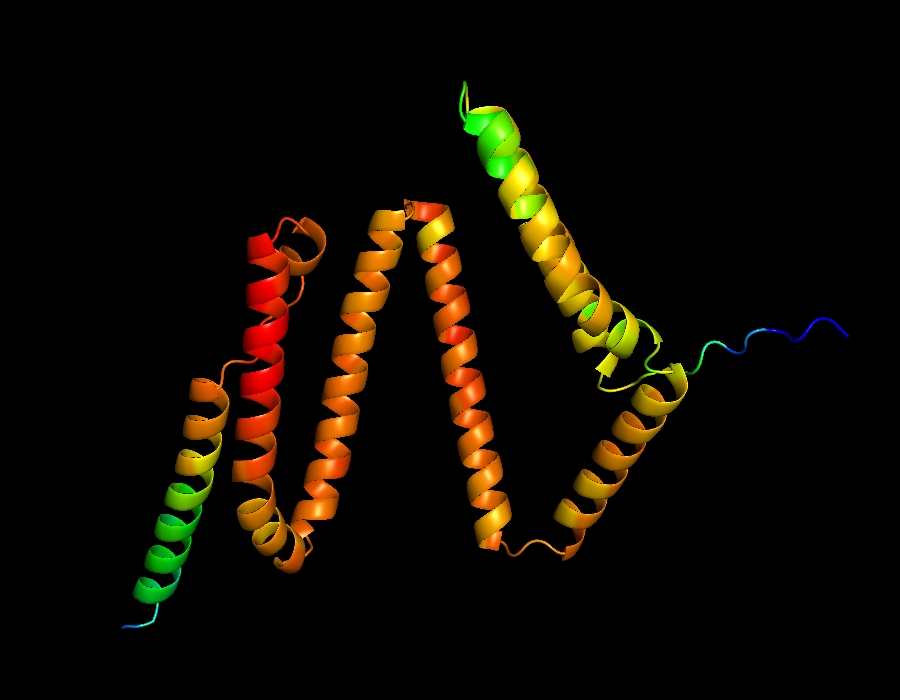

Supplement: Supplementary file 1 [file cimb-46-00701-s001.zip › Supplementary Files/File S1/HB_KAJ91693351.png]

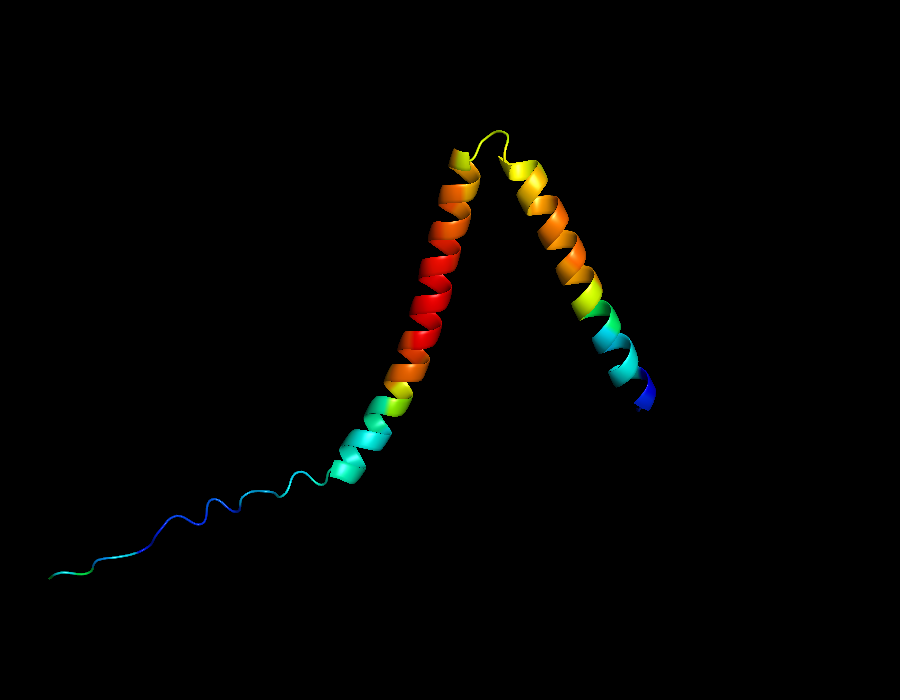

Supplement: Supplementary file 1 [file cimb-46-00701-s001.zip › Supplementary Files/File S1/HB_KAJ91703691.png]

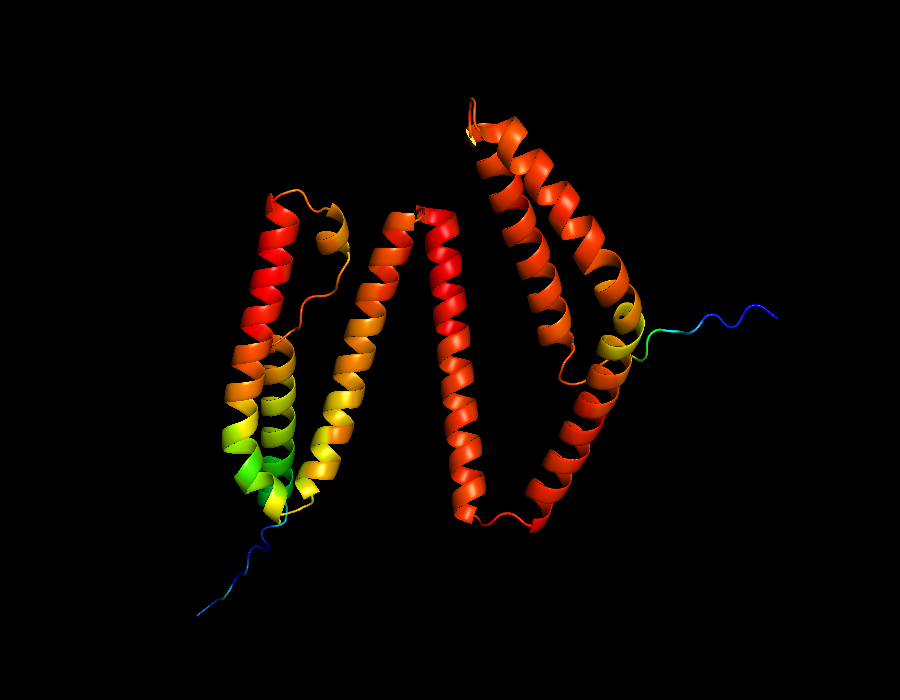

Supplement: Supplementary file 1 [file cimb-46-00701-s001.zip › Supplementary Files/File S1/HB_KAJ91761871.png]

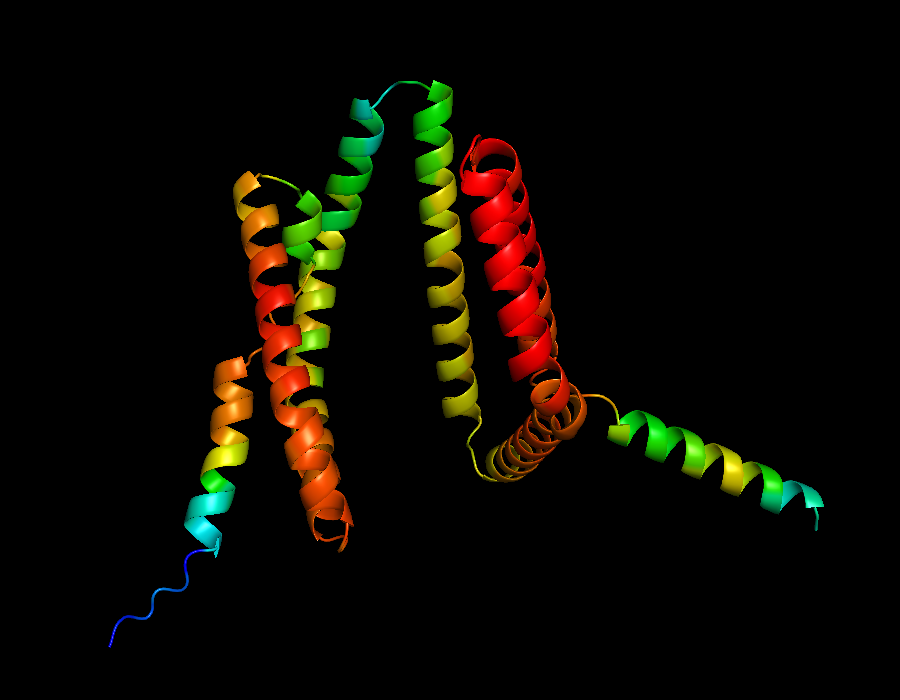

Supplement: Supplementary file 1 [file cimb-46-00701-s001.zip › Supplementary Files/File S1/HB_KAJ91802621.png]

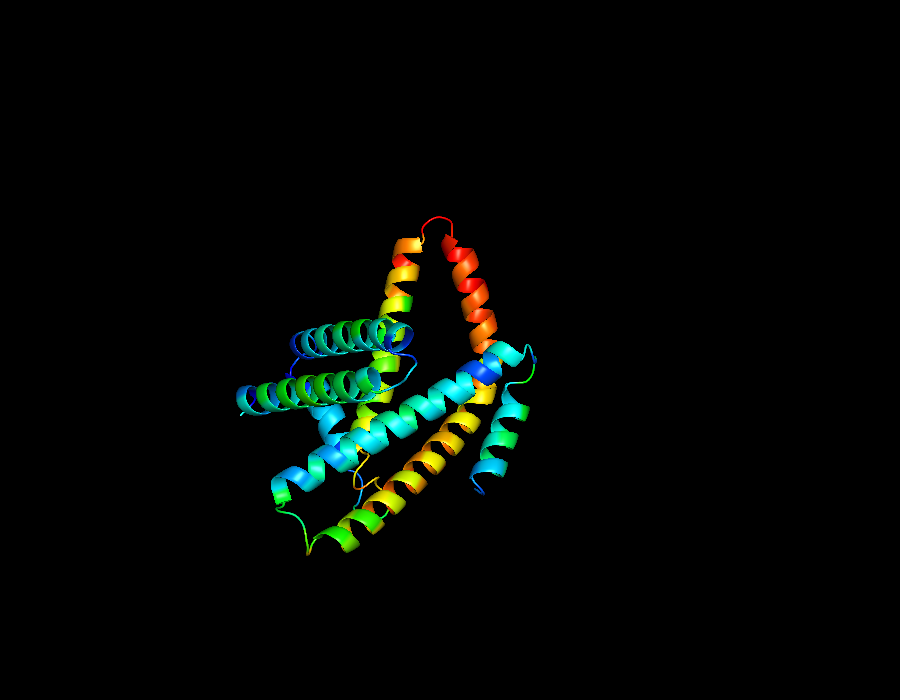

Supplement: Supplementary file 1 [file cimb-46-00701-s001.zip › Supplementary Files/File S1/HB_KAJ91859291.png]

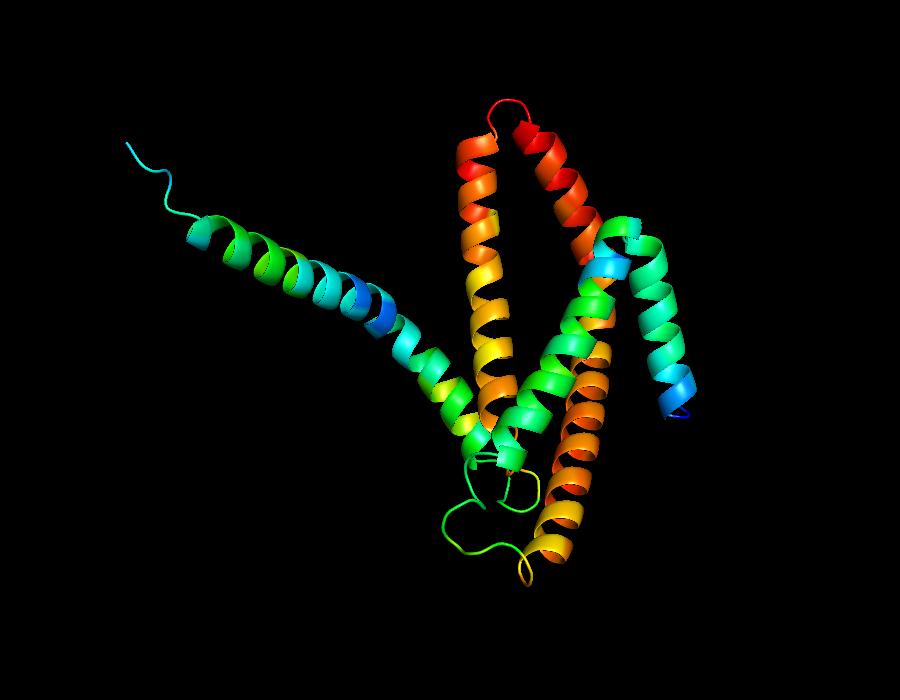

Supplement: Supplementary file 1 [file cimb-46-00701-s001.zip › Supplementary Files/File S1/HB_KAJ91859331.png]

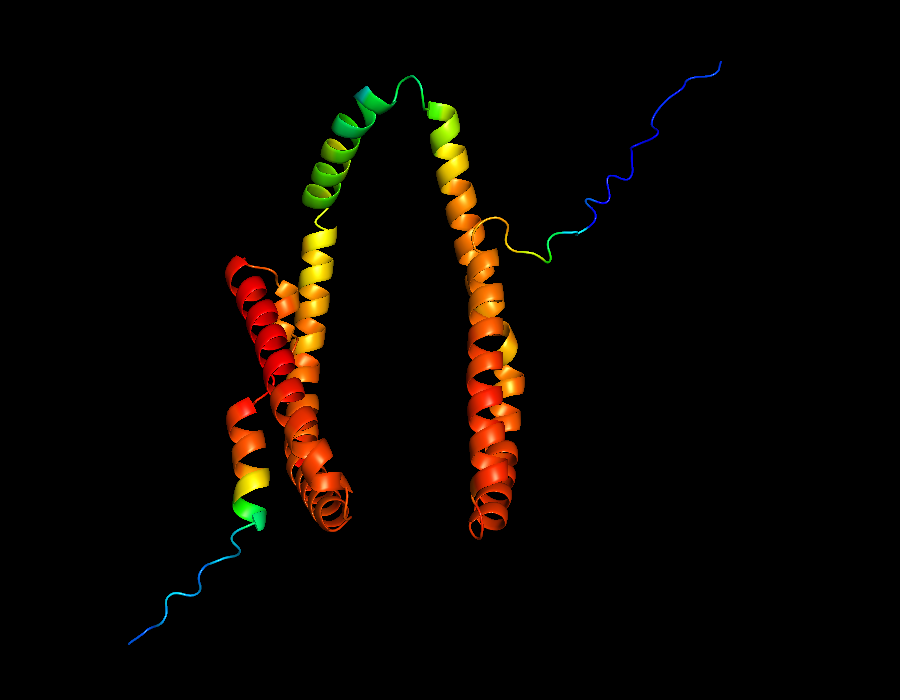

Supplement: Supplementary file 1 [file cimb-46-00701-s001.zip › Supplementary Files/File S1/HB_KAJ91877631.png]

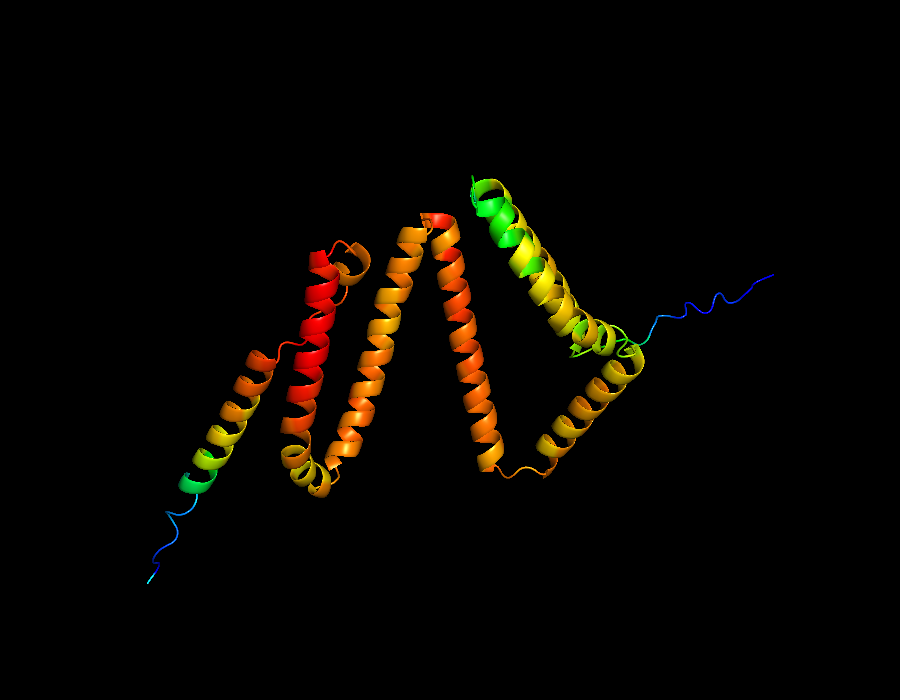

Supplement: Supplementary file 1 [file cimb-46-00701-s001.zip › Supplementary Files/File S1/JC_XP_0120856771.png]

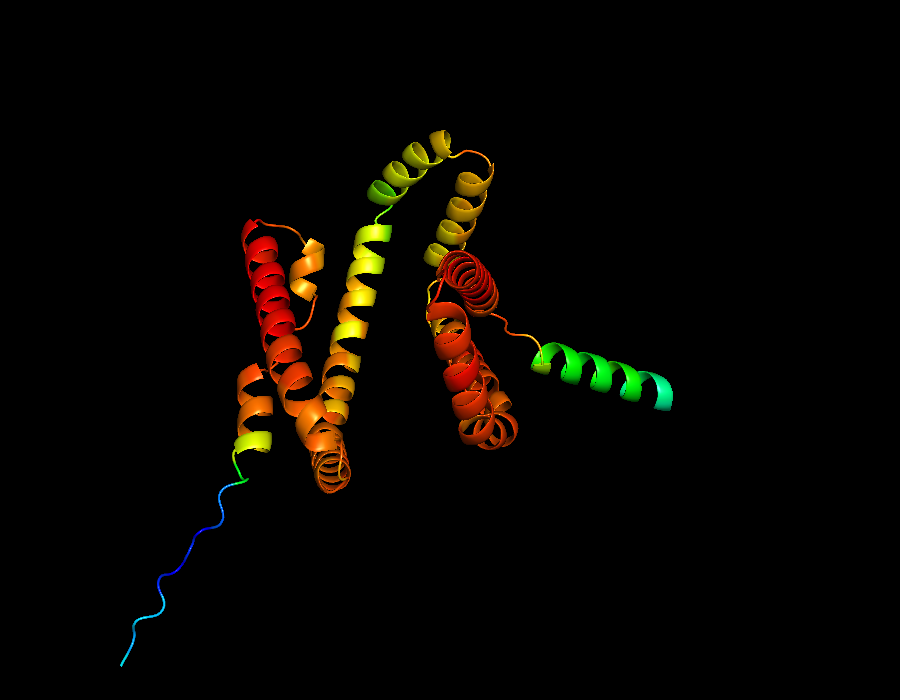

Supplement: Supplementary file 1 [file cimb-46-00701-s001.zip › Supplementary Files/File S1/JC_XP_0120890111.png]

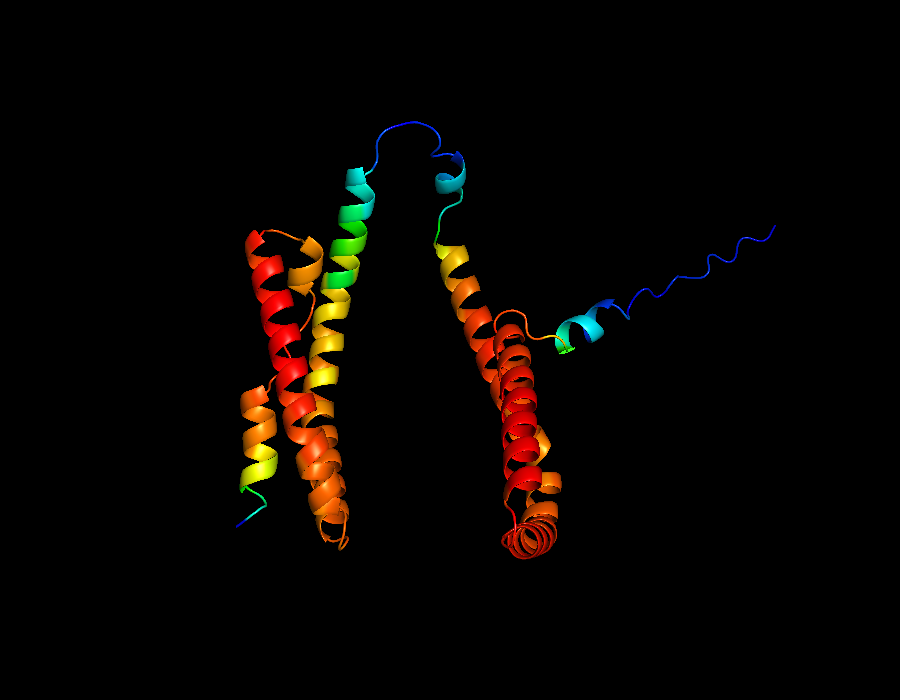

Supplement: Supplementary file 1 [file cimb-46-00701-s001.zip › Supplementary Files/File S1/LS_XP_0237366021.png]

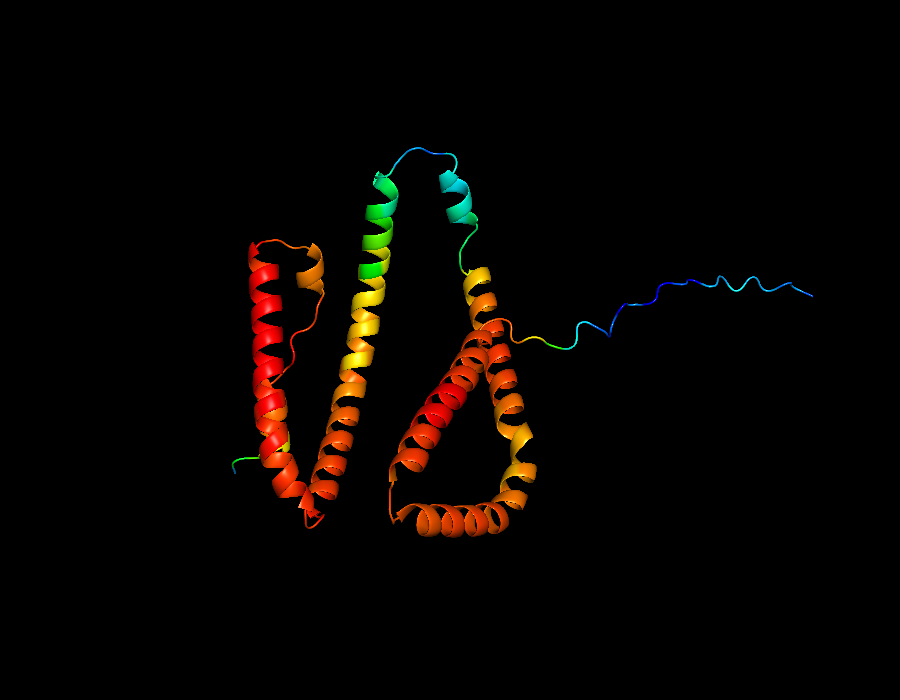

Supplement: Supplementary file 1 [file cimb-46-00701-s001.zip › Supplementary Files/File S1/LS_XP_0237366221.png]

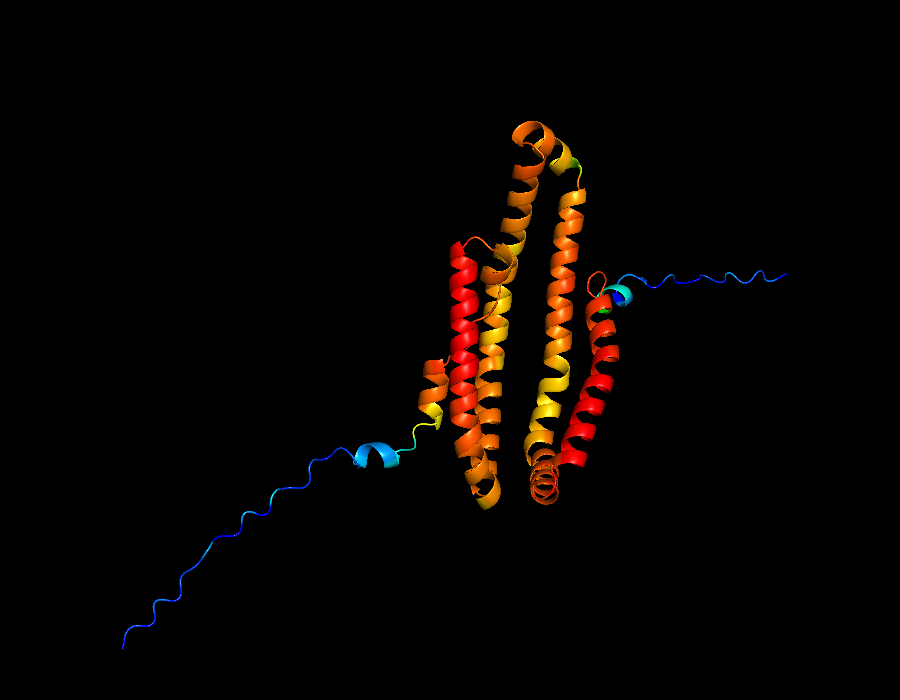

Supplement: Supplementary file 1 [file cimb-46-00701-s001.zip › Supplementary Files/File S1/LS_XP_0237366261.png]

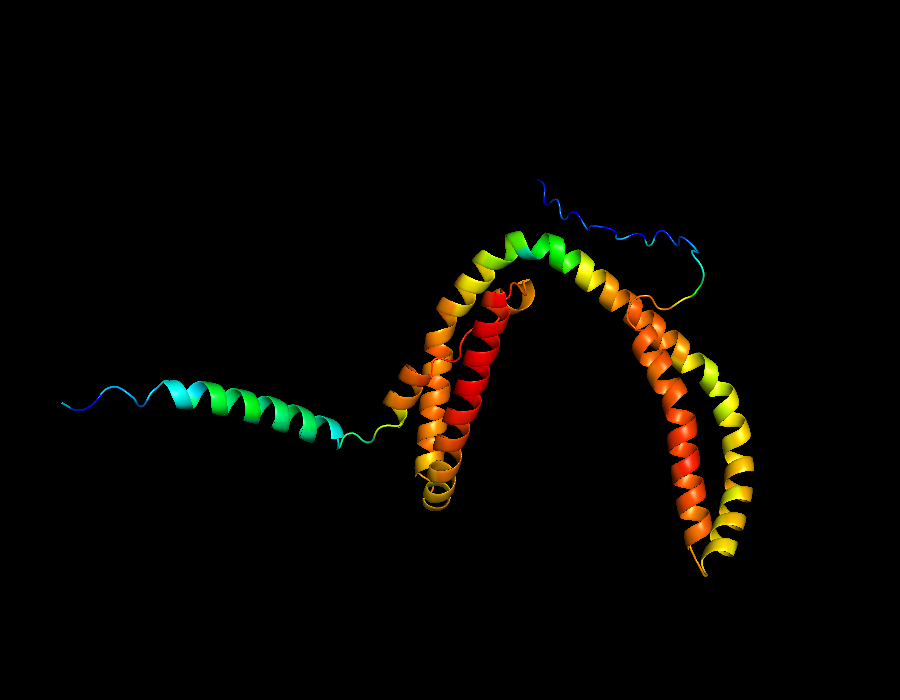

Supplement: Supplementary file 1 [file cimb-46-00701-s001.zip › Supplementary Files/File S1/LS_XP_0237366311.png]

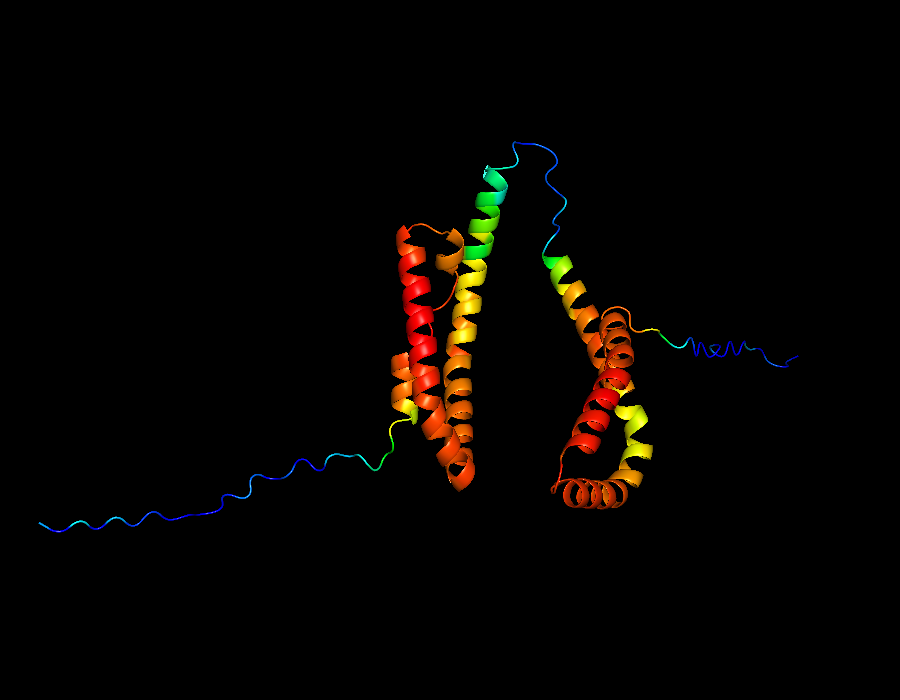

Supplement: Supplementary file 1 [file cimb-46-00701-s001.zip › Supplementary Files/File S1/LS_XP_0237366331.png]

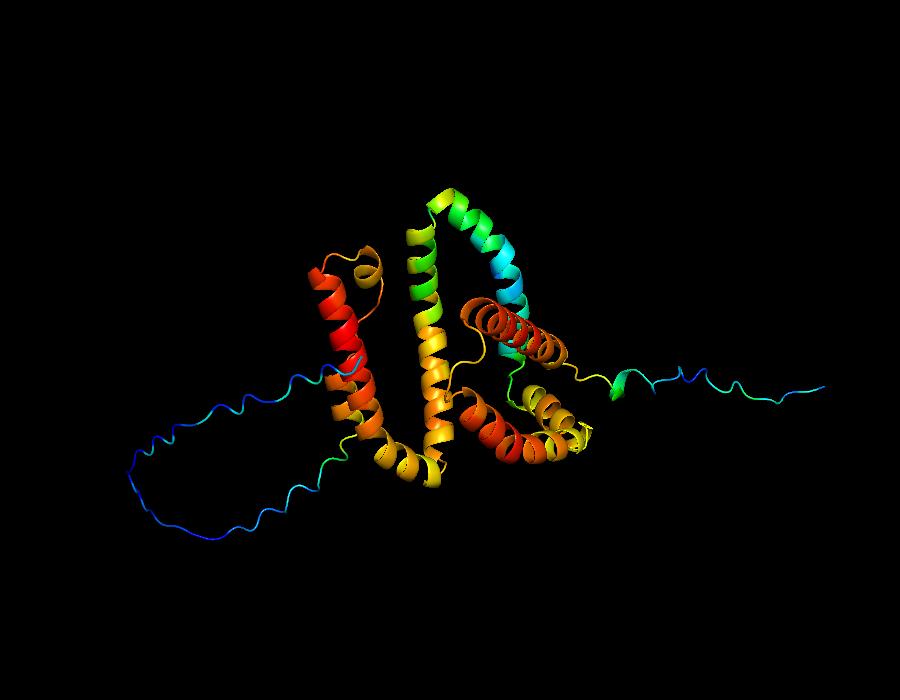

Supplement: Supplementary file 1 [file cimb-46-00701-s001.zip › Supplementary Files/File S1/LS_XP_0237474051.png]

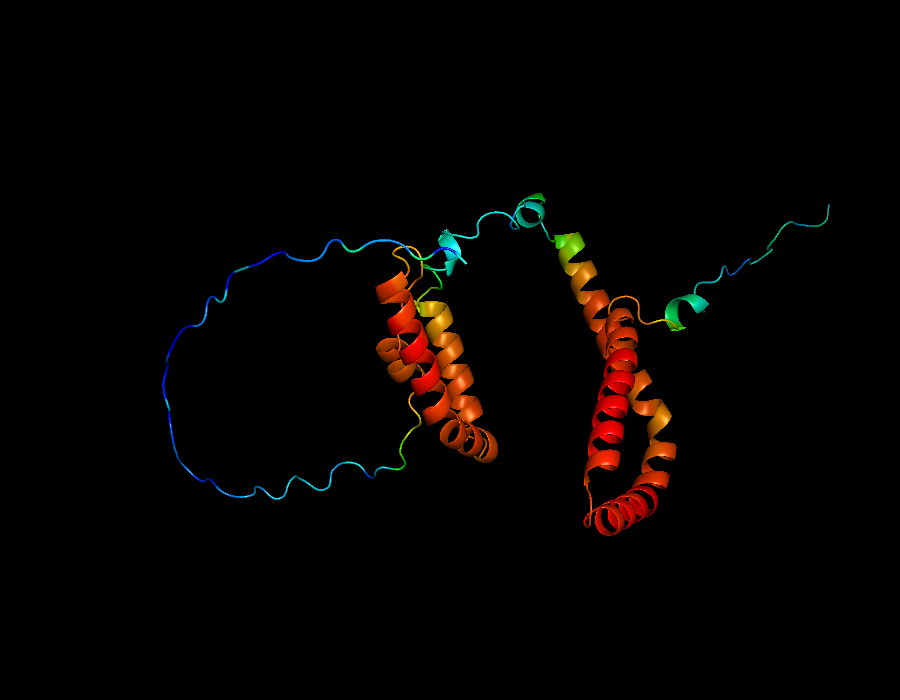

Supplement: Supplementary file 1 [file cimb-46-00701-s001.zip › Supplementary Files/File S1/LS_XP_0237474071.png]

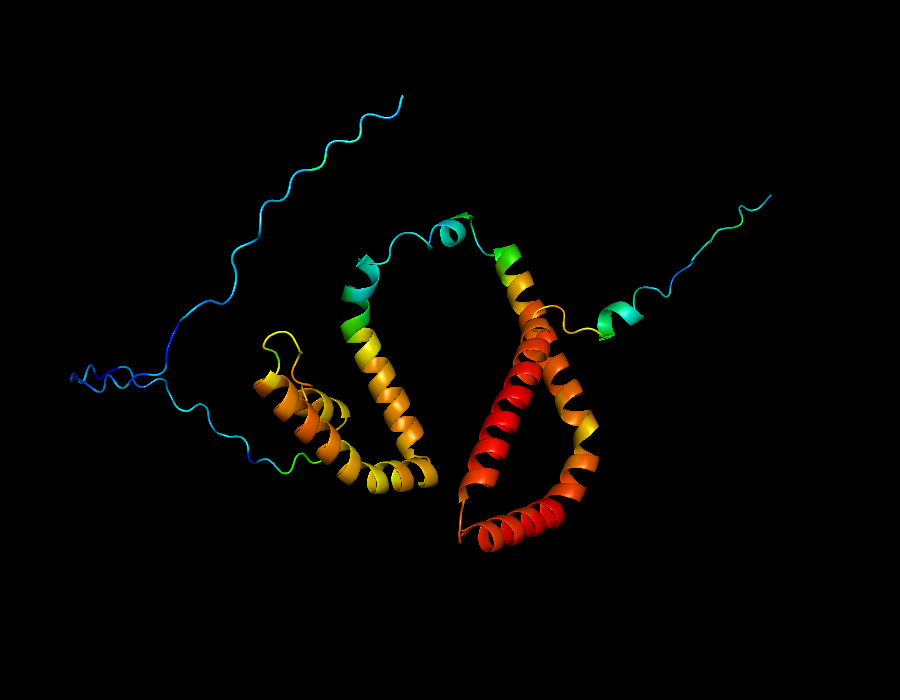

Supplement: Supplementary file 1 [file cimb-46-00701-s001.zip › Supplementary Files/File S1/LS_XP_0237474081.png]

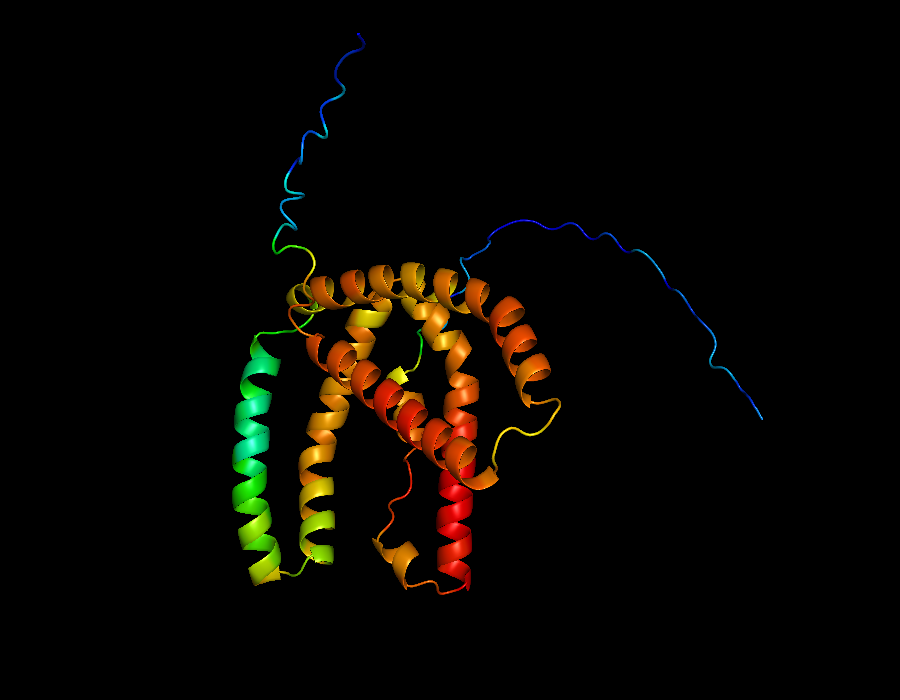

Supplement: Supplementary file 1 [file cimb-46-00701-s001.zip › Supplementary Files/File S1/LS_XP_0237474091.png]

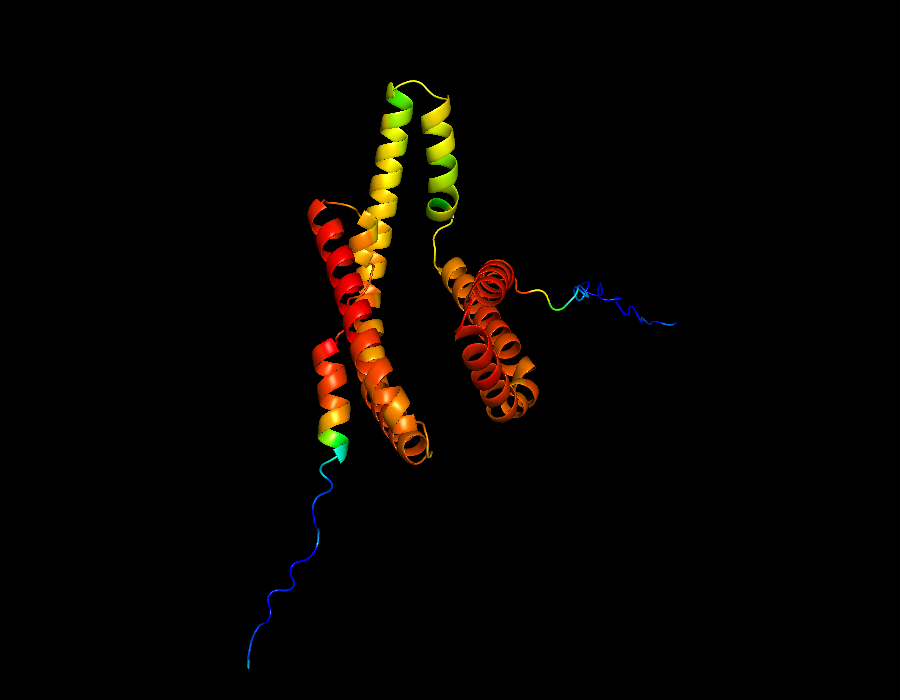

Supplement: Supplementary file 1 [file cimb-46-00701-s001.zip › Supplementary Files/File S1/LS_XP_0237718811.png]

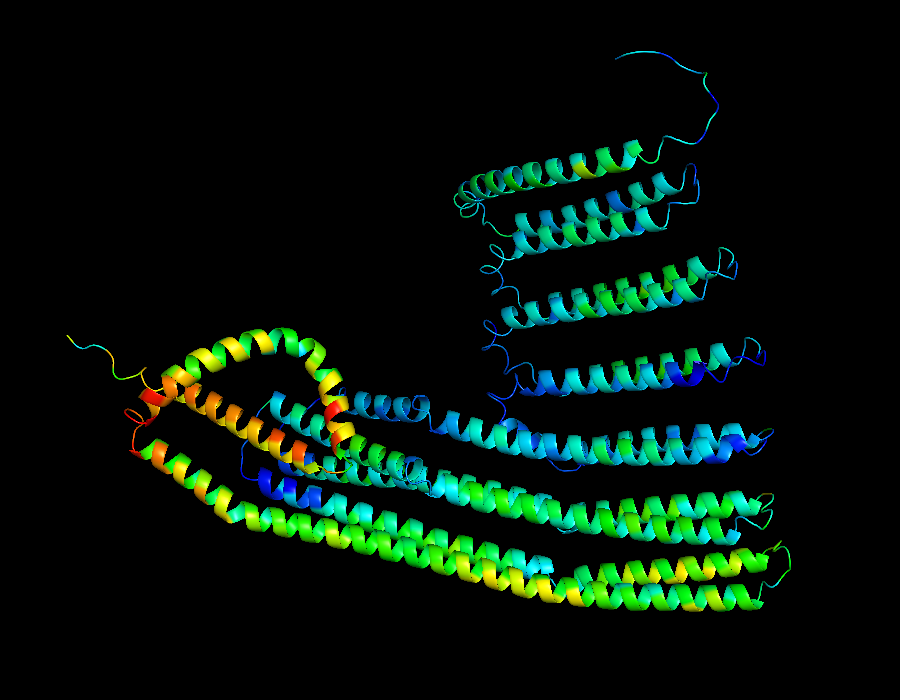

Supplement: Supplementary file 1 [file cimb-46-00701-s001.zip › Supplementary Files/File S1/LS_XP_0427537971.png]

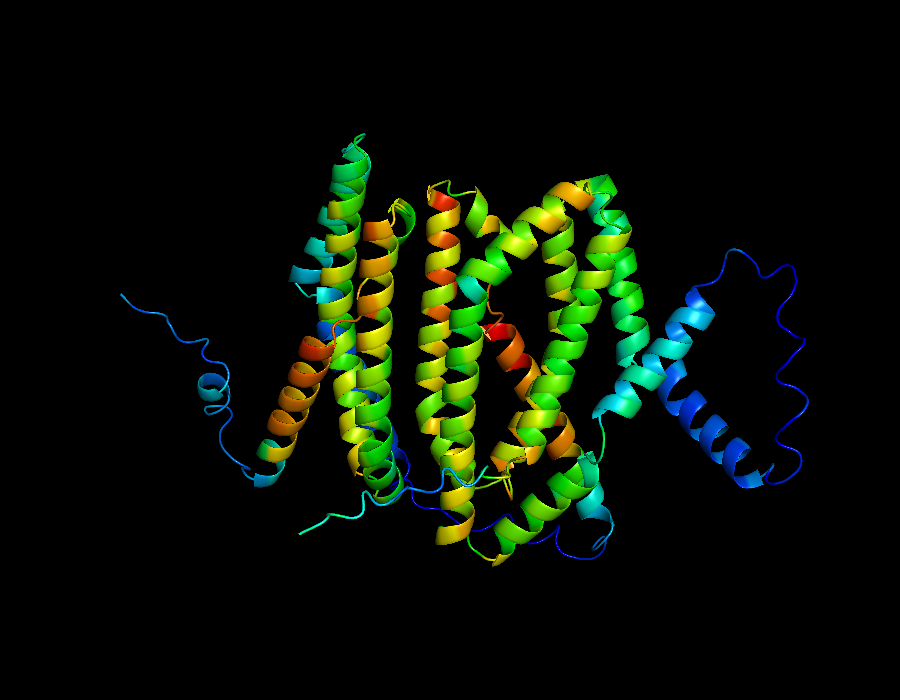

Supplement: Supplementary file 1 [file cimb-46-00701-s001.zip › Supplementary Files/File S1/LS_XP_0526221391.png]

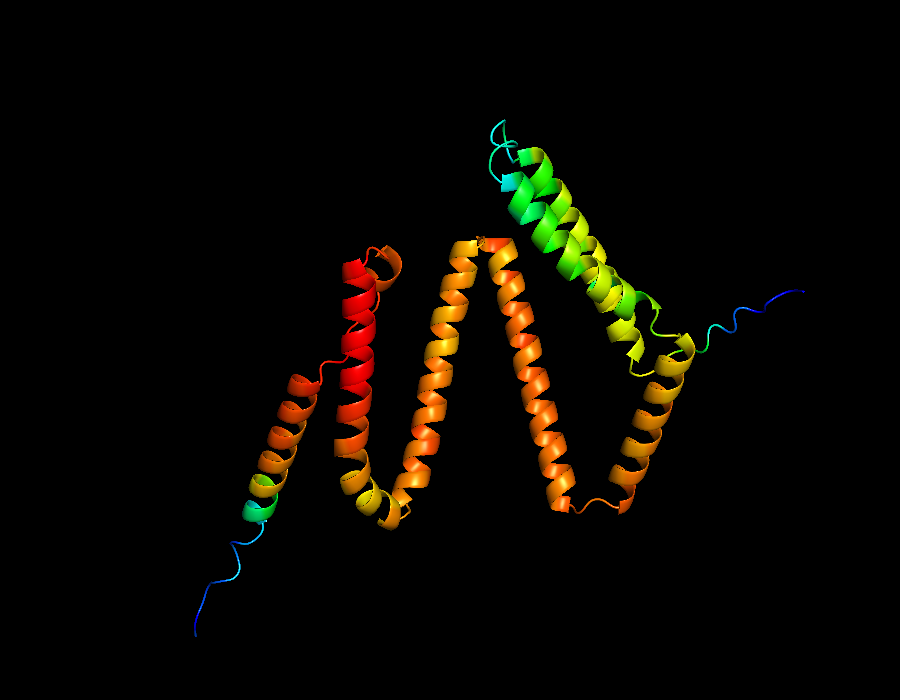

Supplement: Supplementary file 1 [file cimb-46-00701-s001.zip › Supplementary Files/File S1/ME_KAG86416151.png]

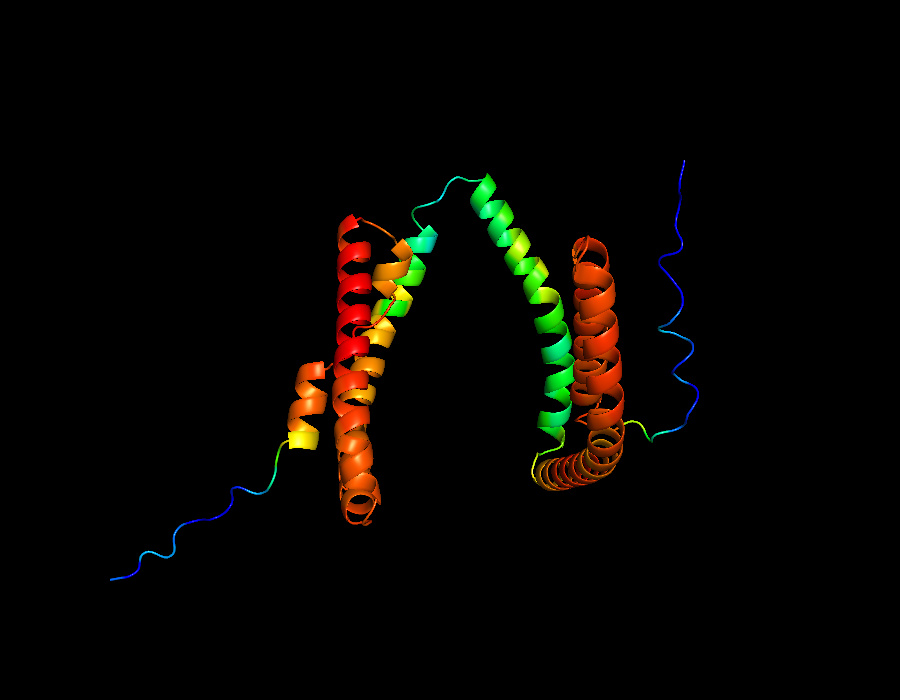

Supplement: Supplementary file 1 [file cimb-46-00701-s001.zip › Supplementary Files/File S1/ME_KAG86482811.png]

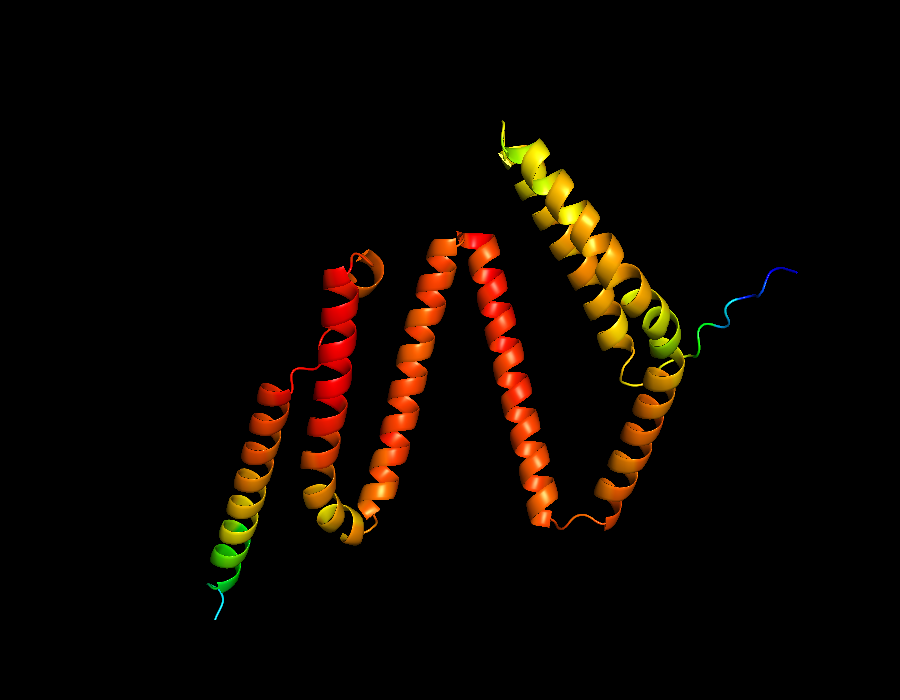

Supplement: Supplementary file 1 [file cimb-46-00701-s001.zip › Supplementary Files/File S1/ME_OAY323611.png]

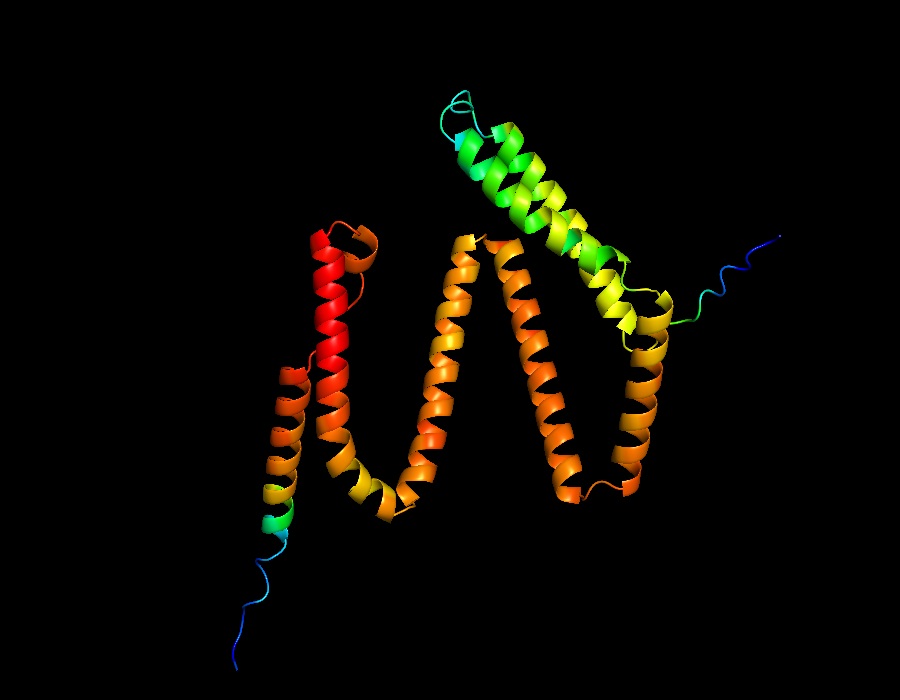

Supplement: Supplementary file 1 [file cimb-46-00701-s001.zip › Supplementary Files/File S1/ME_OAY343171.png]

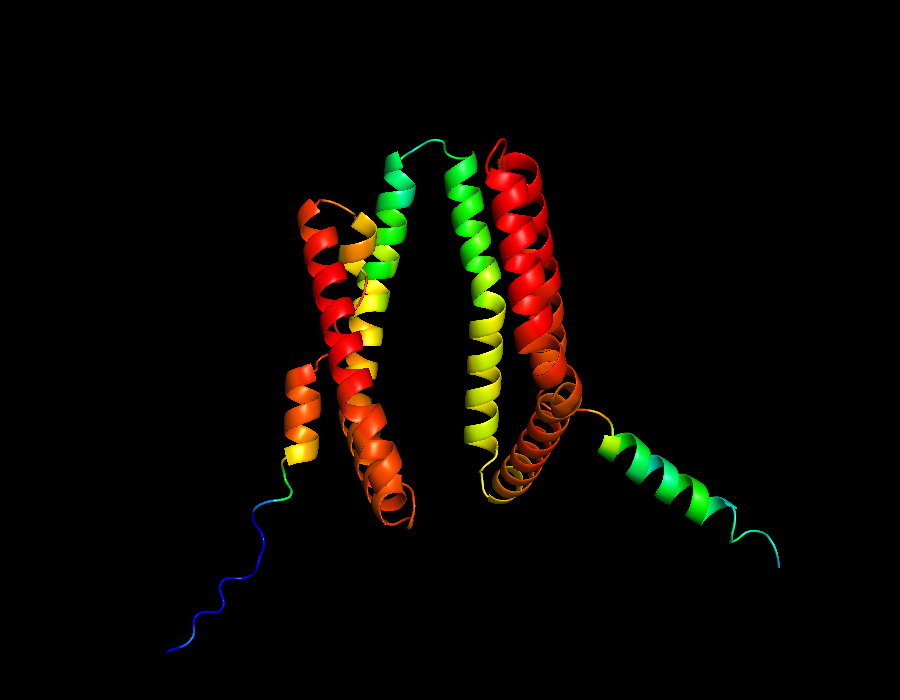

Supplement: Supplementary file 1 [file cimb-46-00701-s001.zip › Supplementary Files/File S1/ME_OAY440431.png]

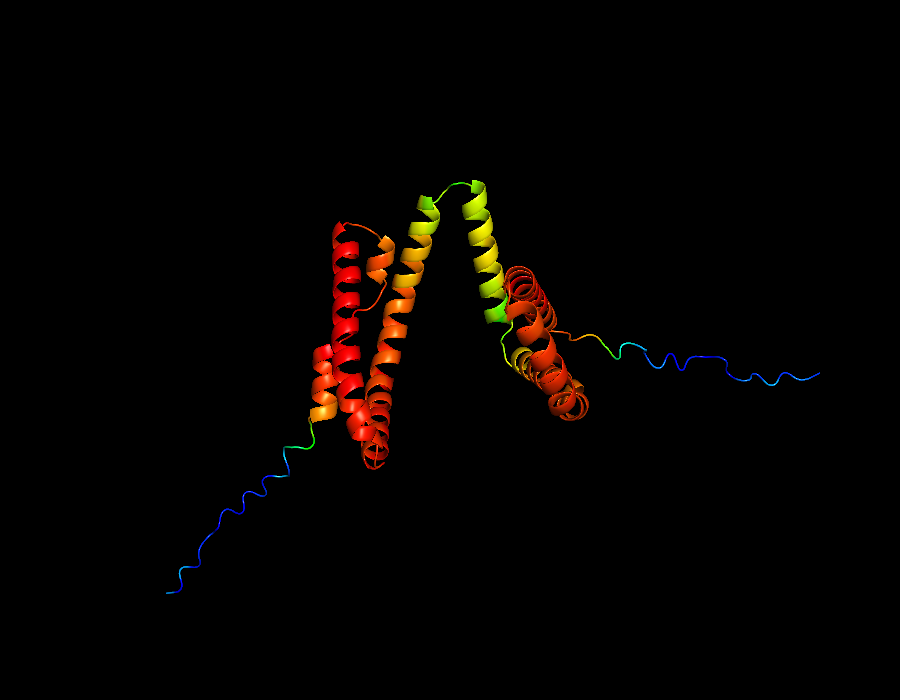

Supplement: Supplementary file 1 [file cimb-46-00701-s001.zip › Supplementary Files/File S1/ME_OAY495331.png]

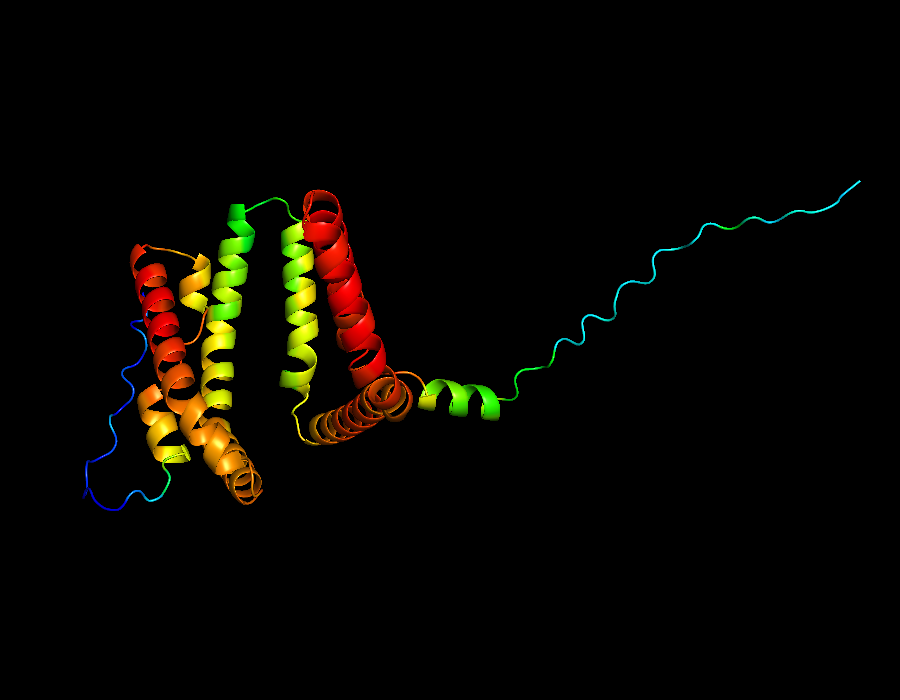

Supplement: Supplementary file 1 [file cimb-46-00701-s001.zip › Supplementary Files/File S1/OS_XP_0156392191.png]

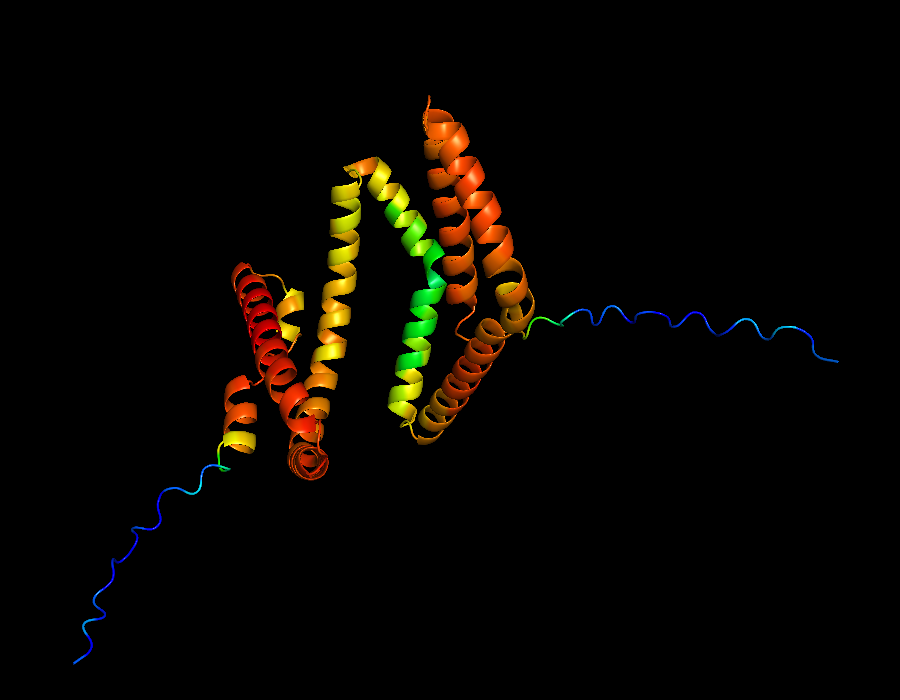

Supplement: Supplementary file 1 [file cimb-46-00701-s001.zip › Supplementary Files/File S1/OS_XP_0156472111.png]

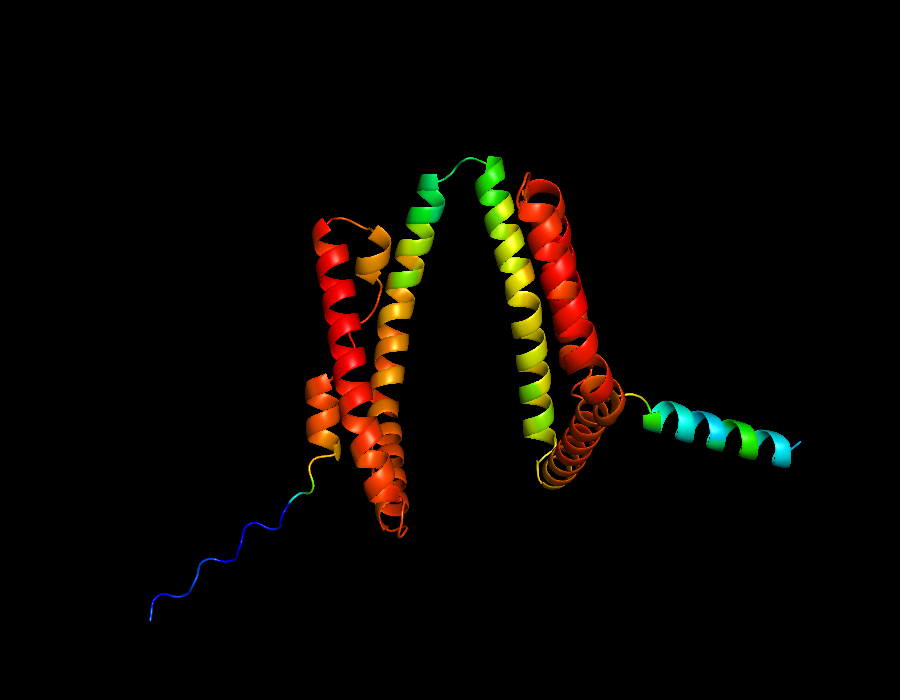

Supplement: Supplementary file 1 [file cimb-46-00701-s001.zip › Supplementary Files/File S1/RC_XP_0025124271.png]

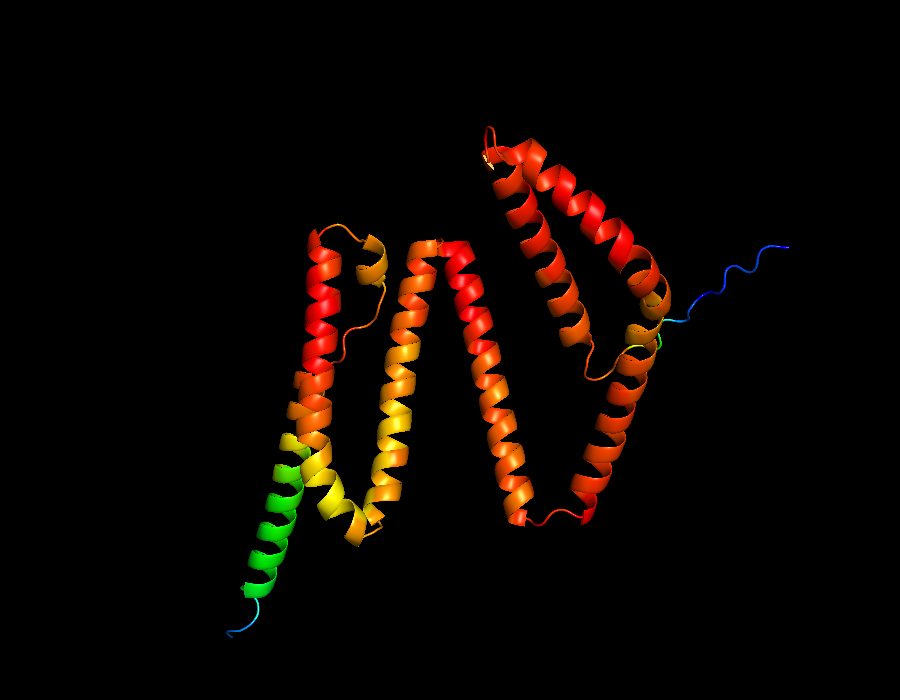

Supplement: Supplementary file 1 [file cimb-46-00701-s001.zip › Supplementary Files/File S1/RC_XP_0025149171.png]

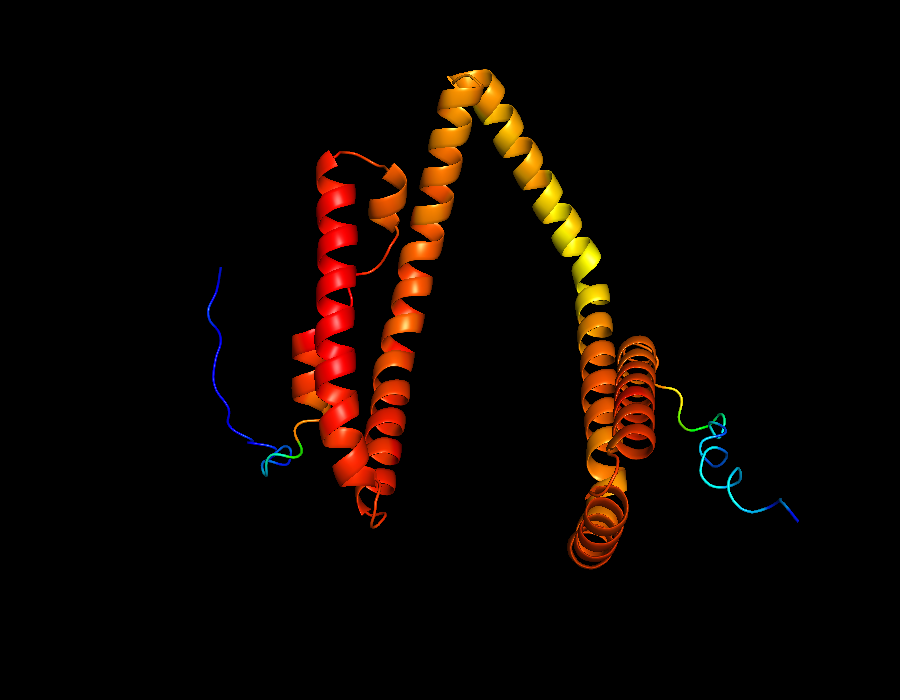

Supplement: Supplementary file 1 [file cimb-46-00701-s001.zip › Supplementary Files/File S1/RC_XP_0025318841.png]

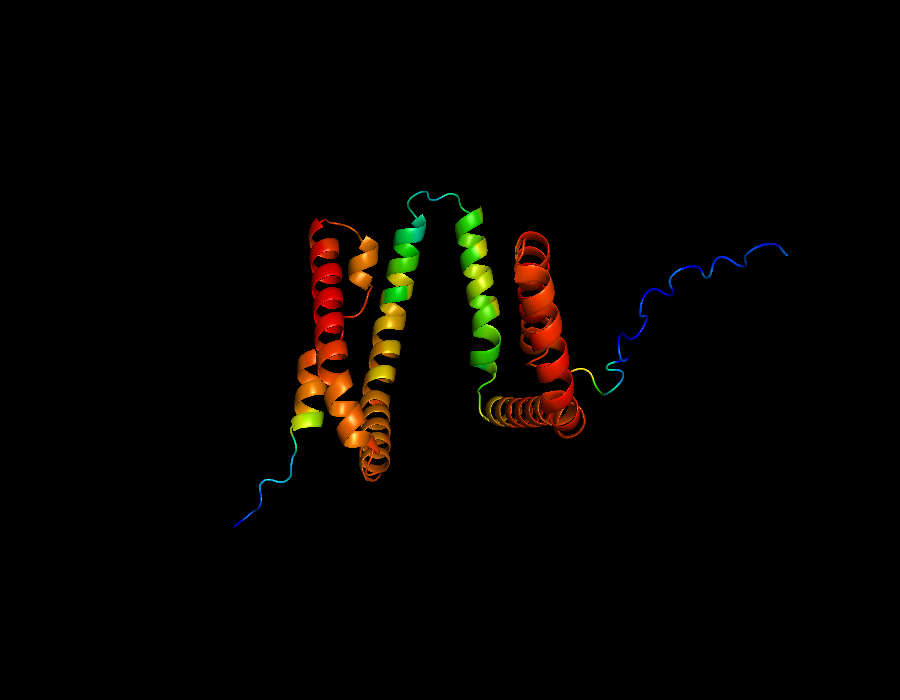

Supplement: Supplementary file 1 [file cimb-46-00701-s001.zip › Supplementary Files/File S1/SL_XP_0042302351.png]

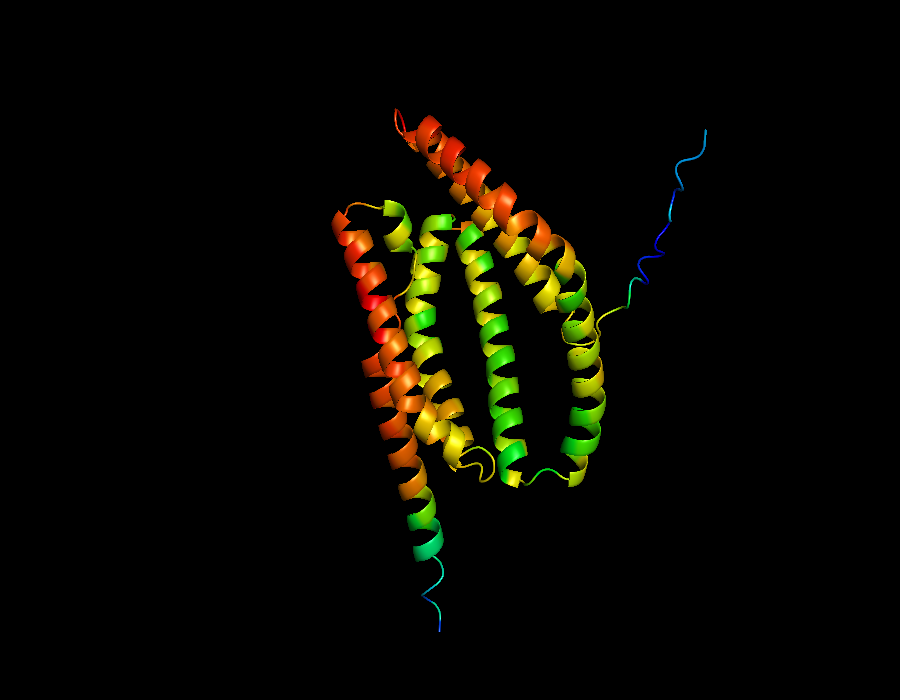

Supplement: Supplementary file 1 [file cimb-46-00701-s001.zip › Supplementary Files/File S1/SL_XP_0042392101.png]

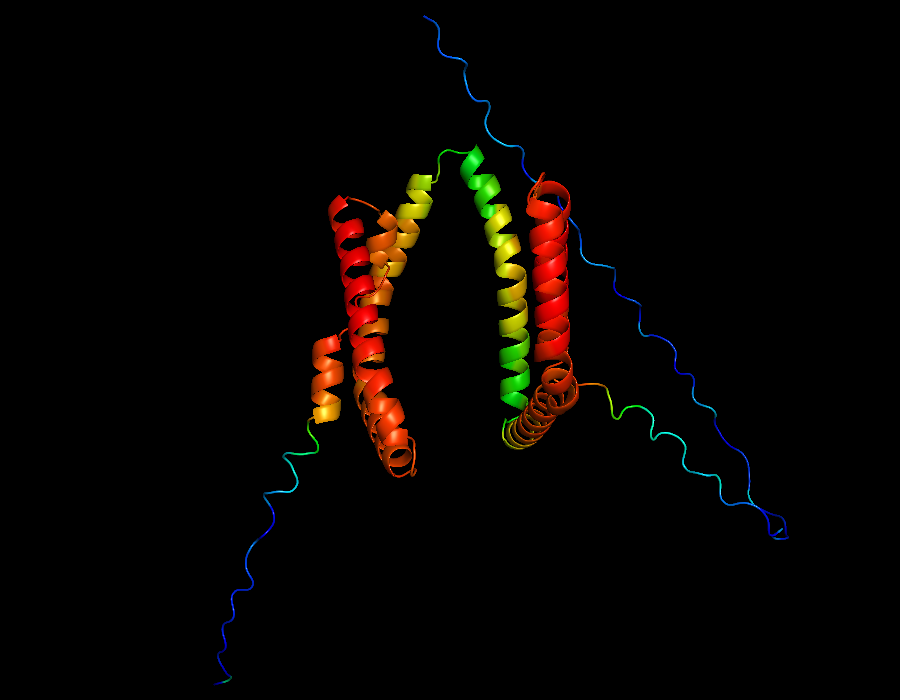

Supplement: Supplementary file 1 [file cimb-46-00701-s001.zip › Supplementary Files/File S1/SL_XP_0042474321.png]

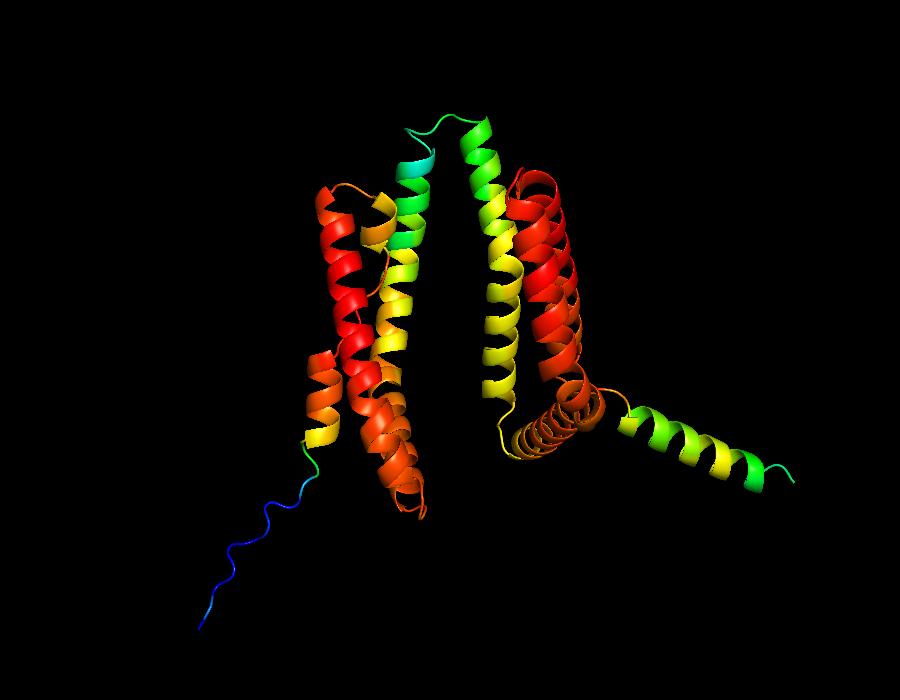

Supplement: Supplementary file 1 [file cimb-46-00701-s001.zip › Supplementary Files/File S1/SY_evmmodelctg000980_np1212723.png]

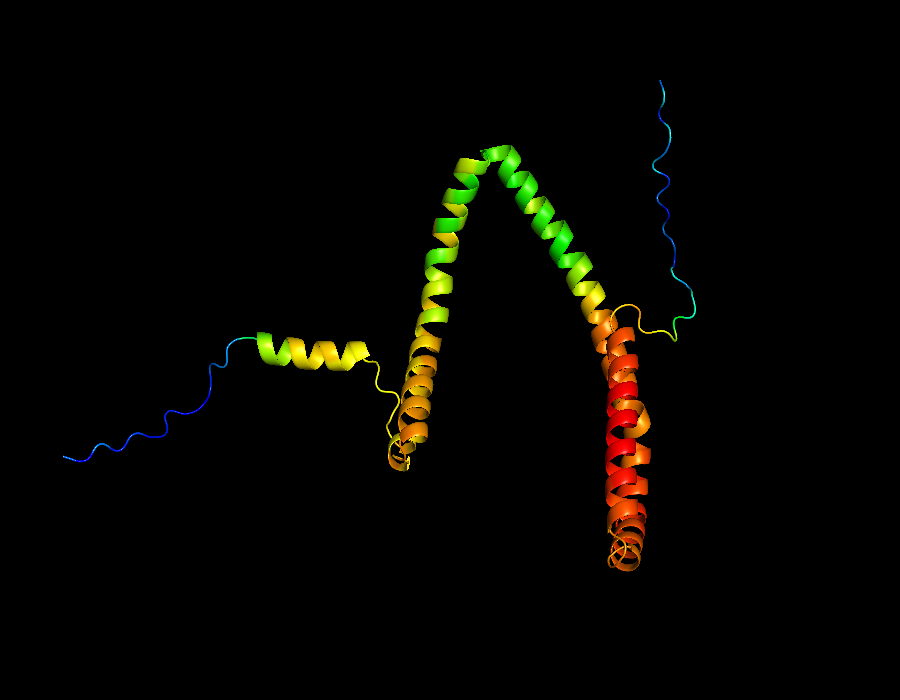

Supplement: Supplementary file 1 [file cimb-46-00701-s001.zip › Supplementary Files/File S1/SY_evmmodelctg001420_np12121548.png]

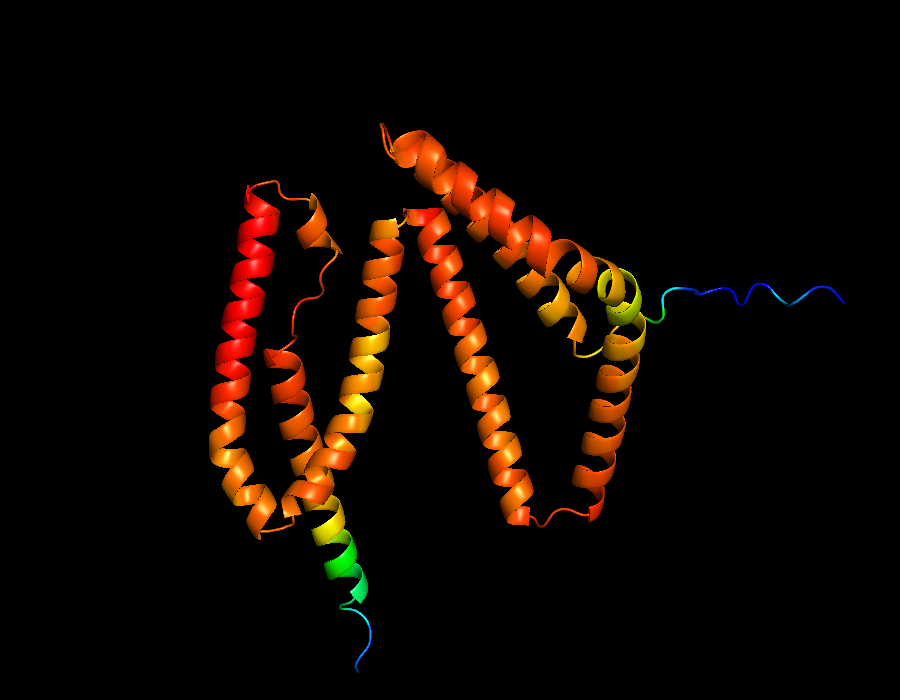

Supplement: Supplementary file 1 [file cimb-46-00701-s001.zip › Supplementary Files/File S1/SY_evmmodelctg001700_np1212147.png]

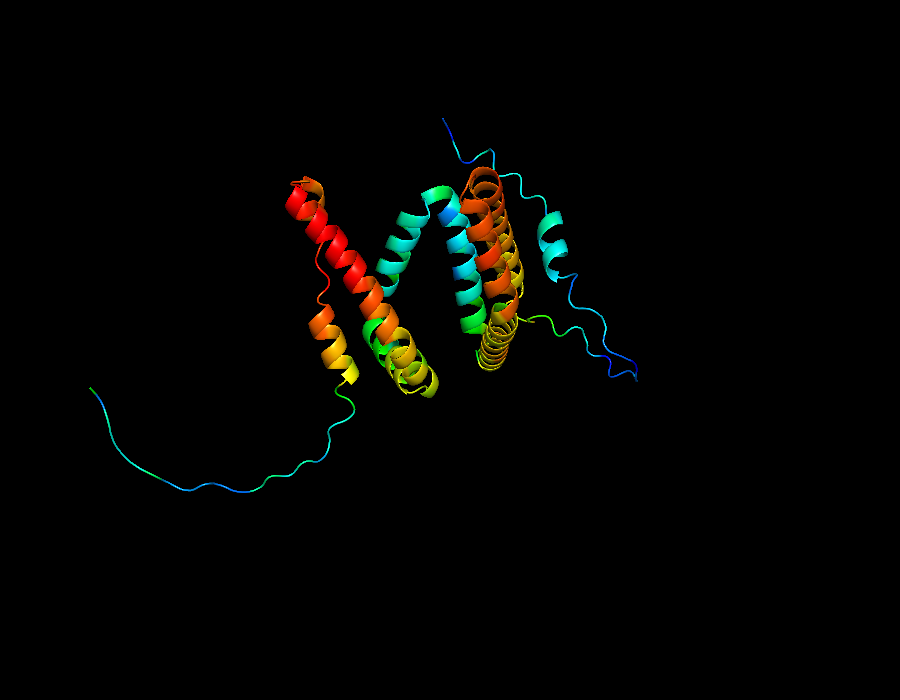

Supplement: Supplementary file 1 [file cimb-46-00701-s001.zip › Supplementary Files/File S1/TK_GWHPBCHF033097.png]

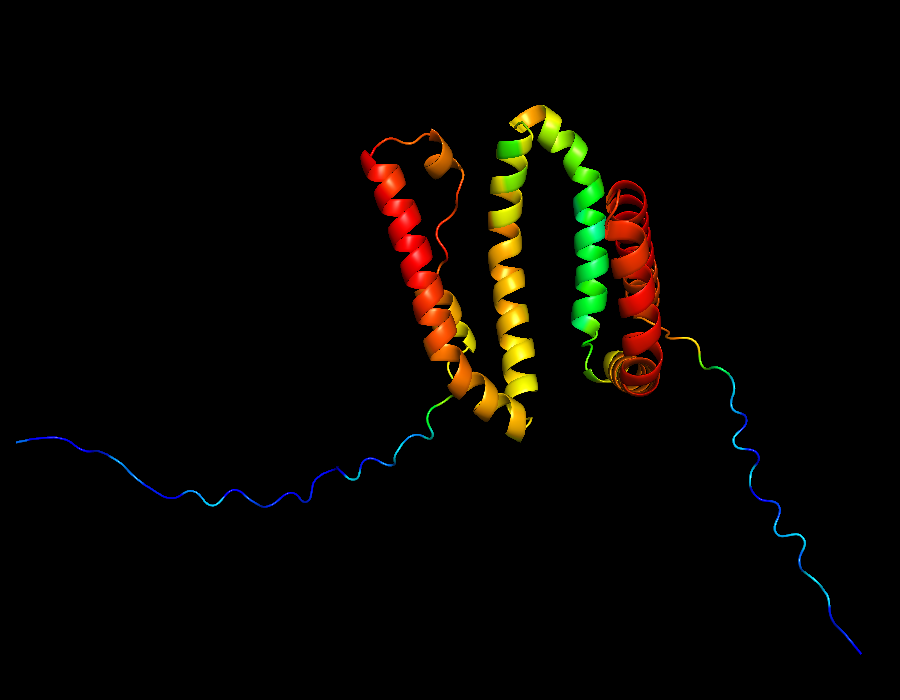

Supplement: Supplementary file 1 [file cimb-46-00701-s001.zip › Supplementary Files/File S1/TK_GWHPBCHF033098.png]

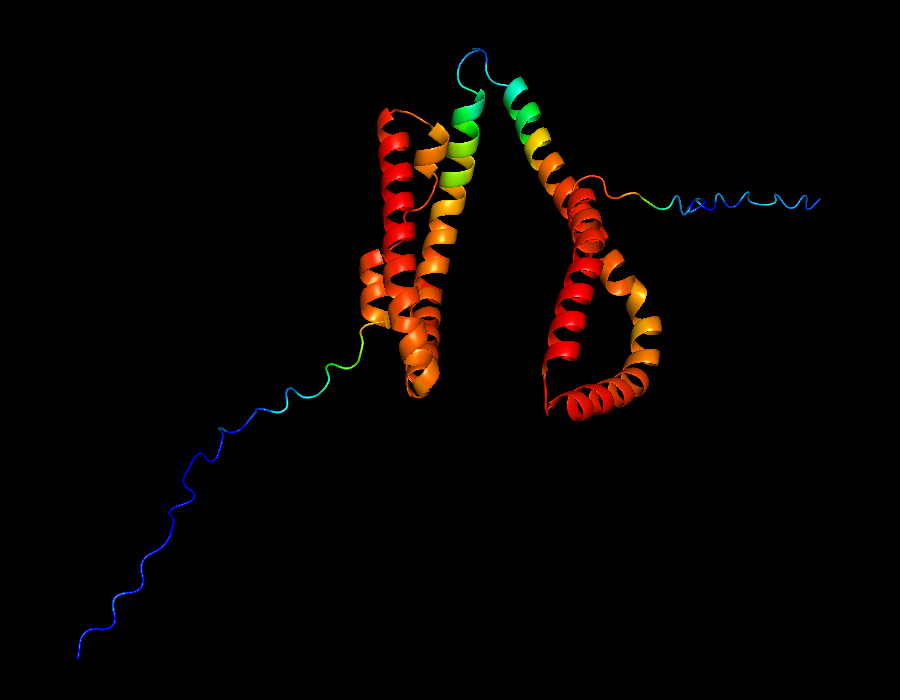

Supplement: Supplementary file 1 [file cimb-46-00701-s001.zip › Supplementary Files/File S1/TK_GWHPBCHF033101.png]

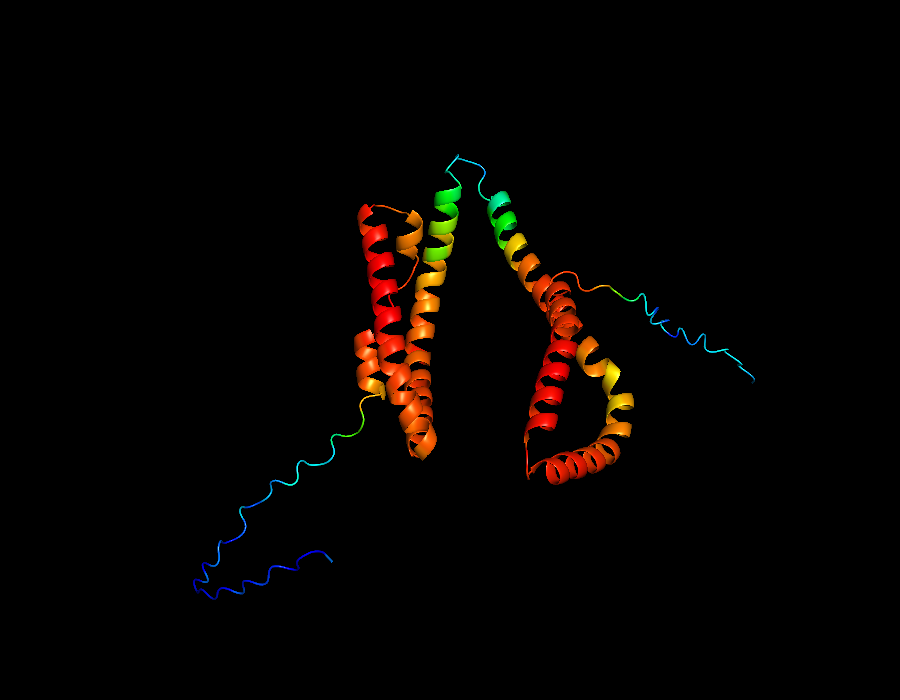

Supplement: Supplementary file 1 [file cimb-46-00701-s001.zip › Supplementary Files/File S1/TK_GWHPBCHF033102.png]

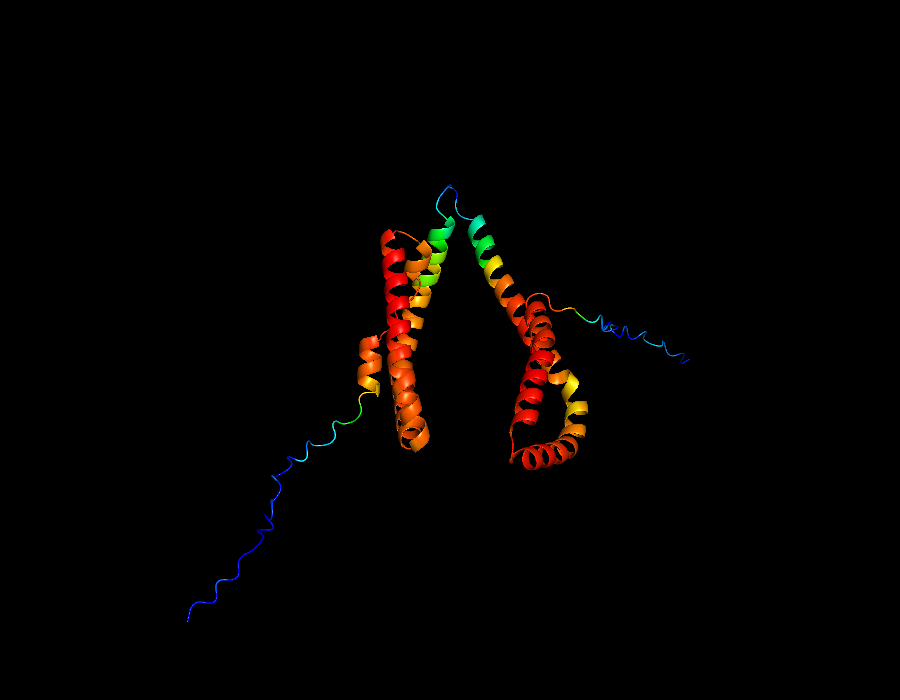

Supplement: Supplementary file 1 [file cimb-46-00701-s001.zip › Supplementary Files/File S1/TK_GWHPBCHF033105.png]

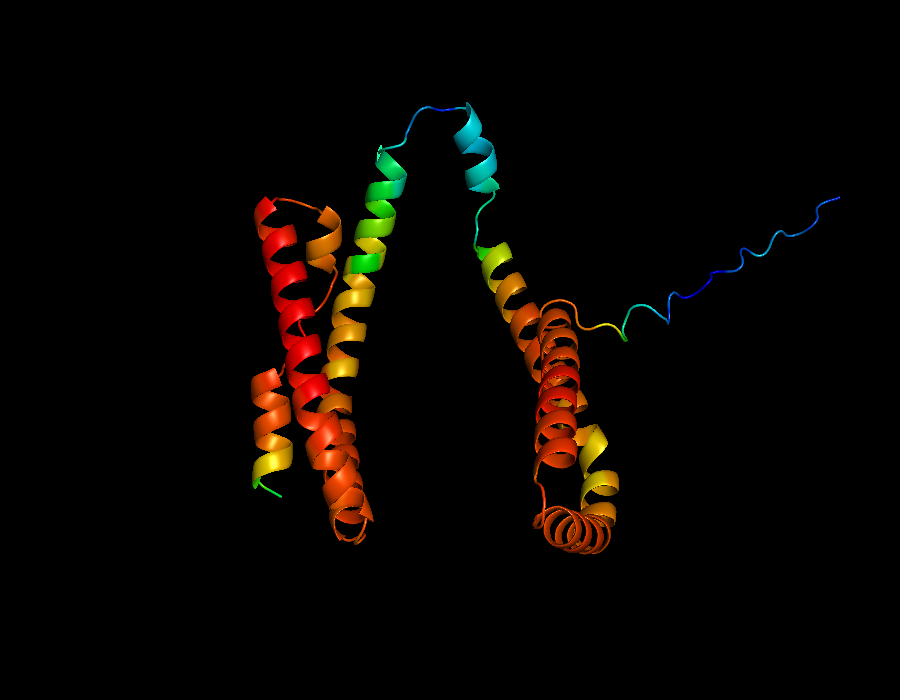

Supplement: Supplementary file 1 [file cimb-46-00701-s001.zip › Supplementary Files/File S1/TK_GWHPBCHF033106.png]

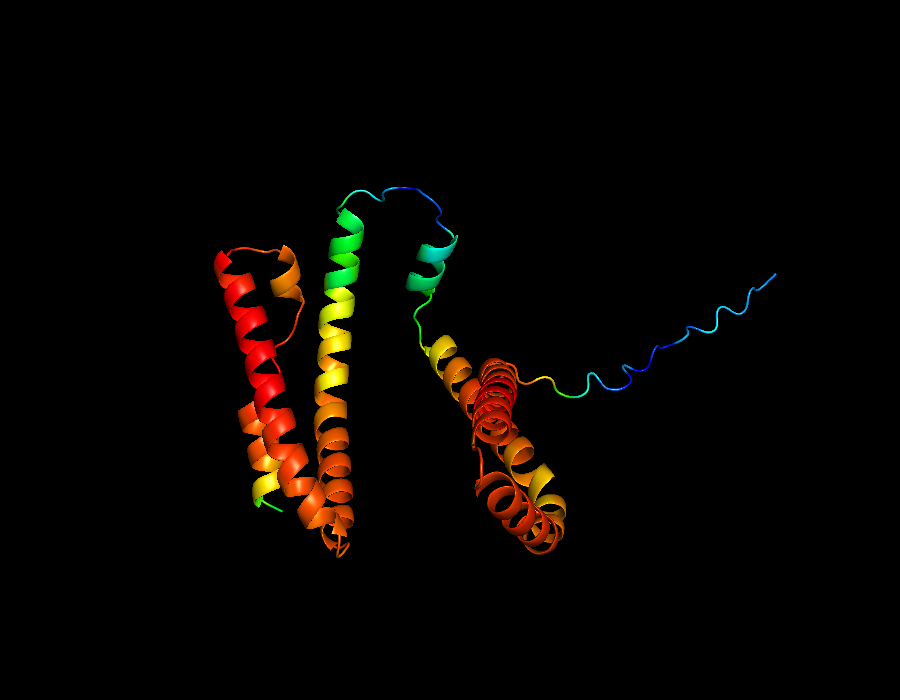

Supplement: Supplementary file 1 [file cimb-46-00701-s001.zip › Supplementary Files/File S1/TK_GWHPBCHF033213.png]

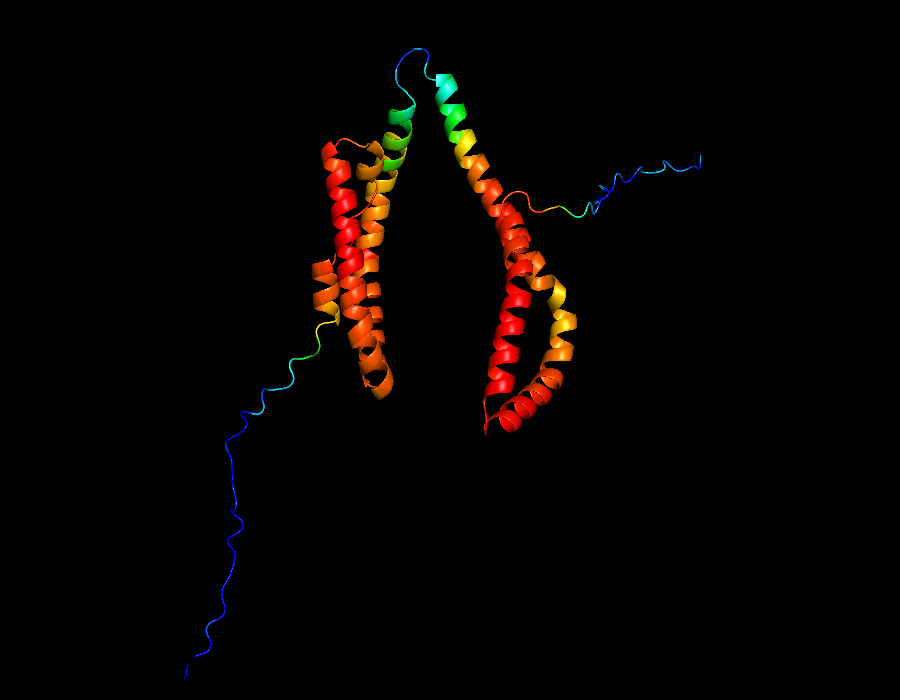

Supplement: Supplementary file 1 [file cimb-46-00701-s001.zip › Supplementary Files/File S1/TK_GWHPBCHF033215.png]

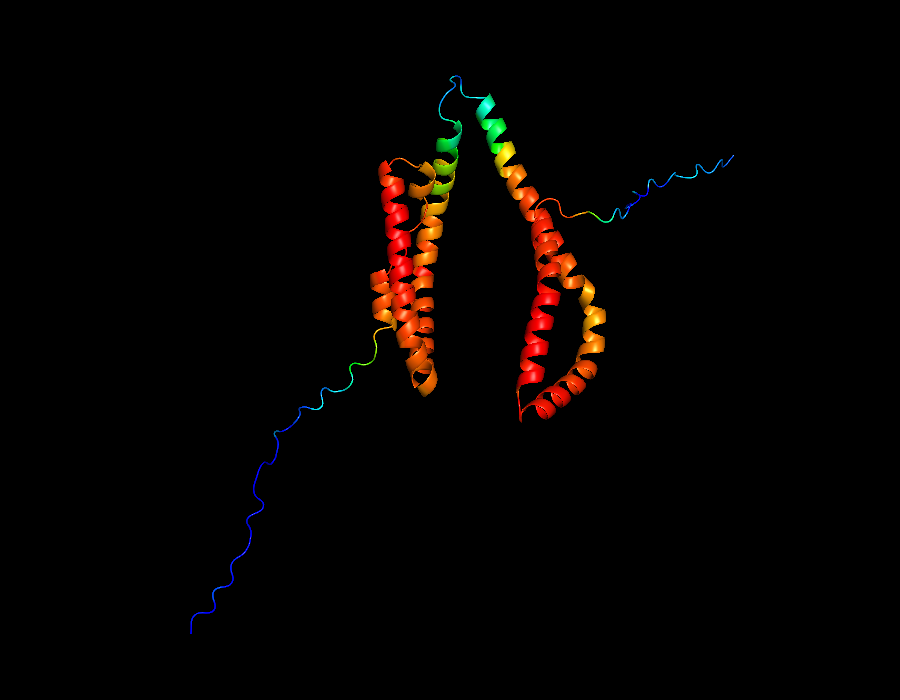

Supplement: Supplementary file 1 [file cimb-46-00701-s001.zip › Supplementary Files/File S1/TK_GWHPBCHF033216.png]

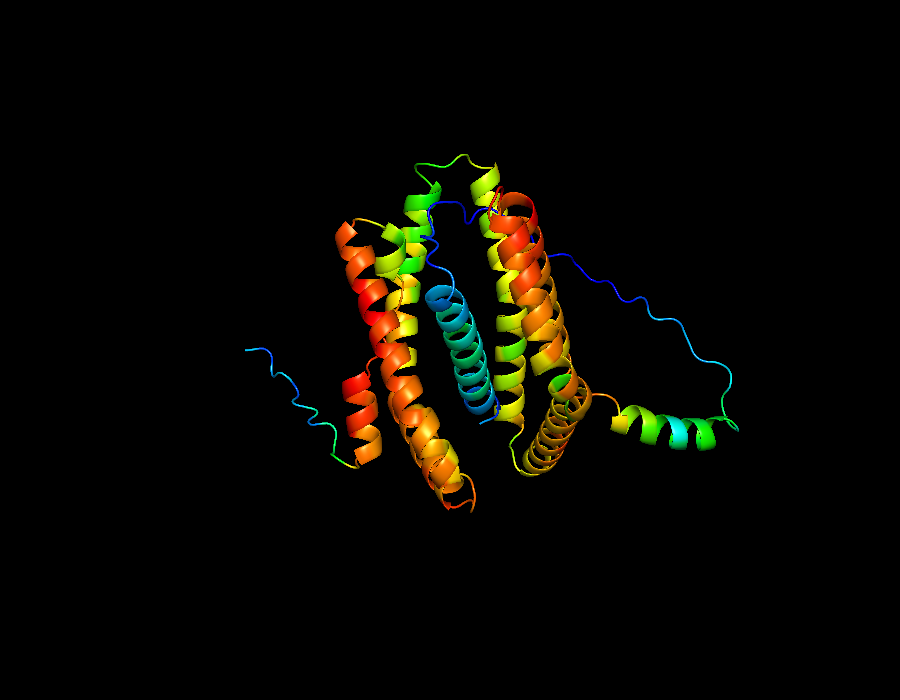

Supplement: Supplementary file 1 [file cimb-46-00701-s001.zip › Supplementary Files/File S1/VF_GWHPAAEU028184.png]

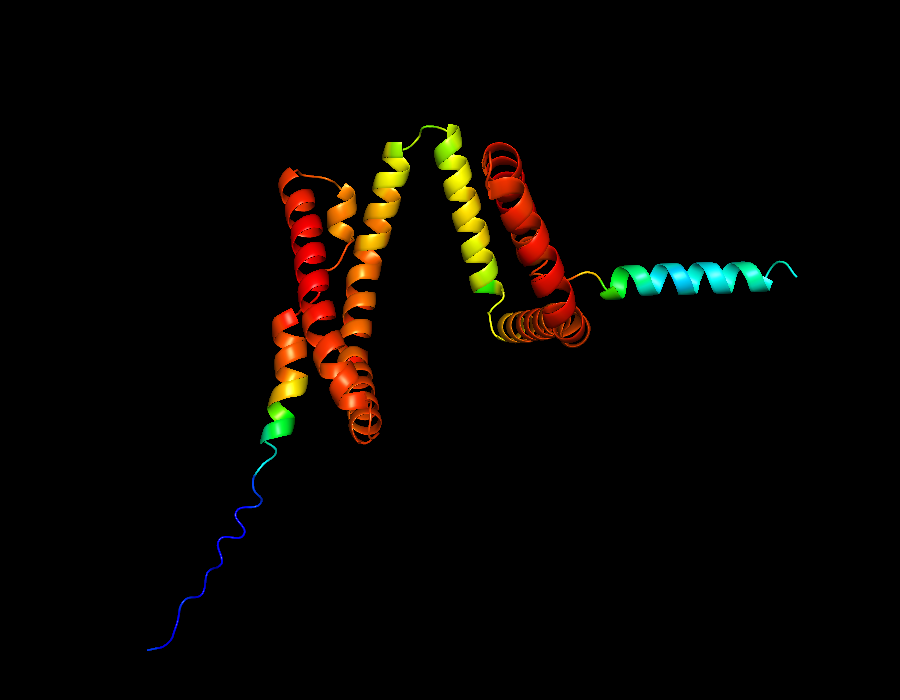

Supplement: Supplementary file 1 [file cimb-46-00701-s001.zip › Supplementary Files/File S1/VF_GWHPAAEU046534.png]

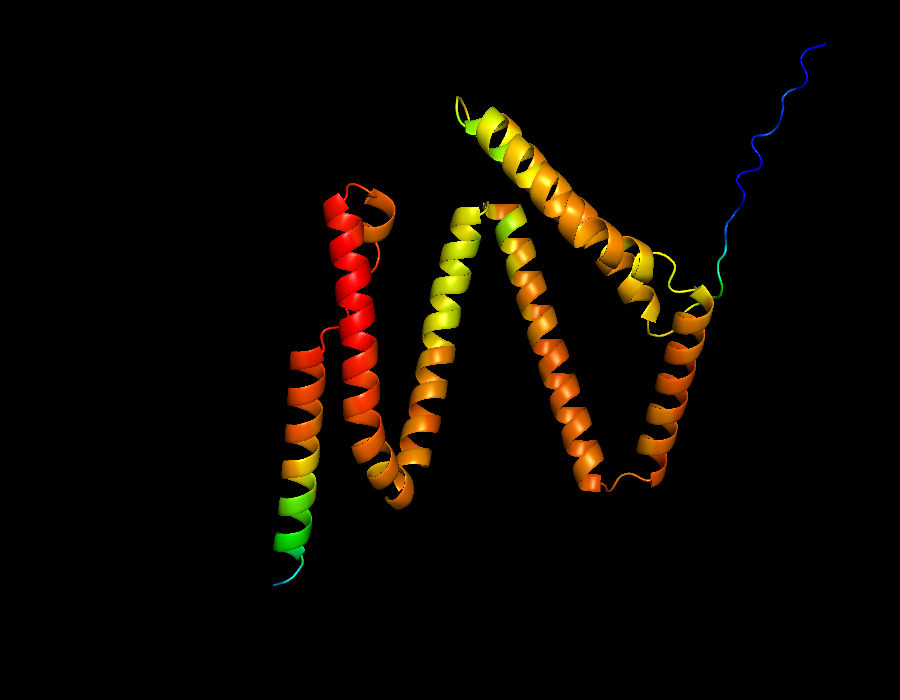

Supplement: Supplementary file 1 [file cimb-46-00701-s001.zip › Supplementary Files/File S1/VF_GWHPAAEU055987.png]

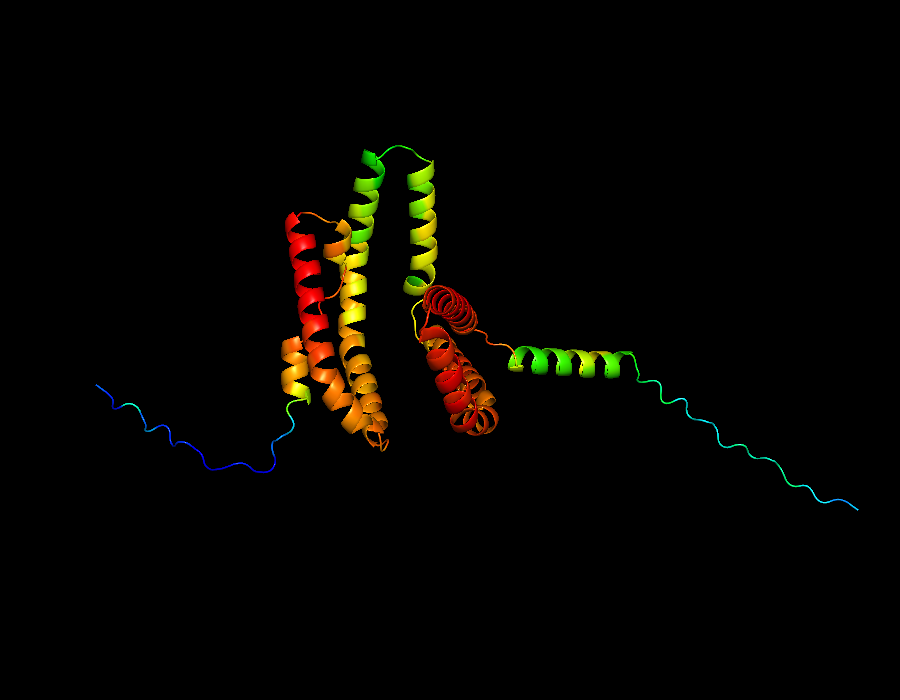

Supplement: Supplementary file 1 [file cimb-46-00701-s001.zip › Supplementary Files/File S1/ZM_NP_0011498341.png]

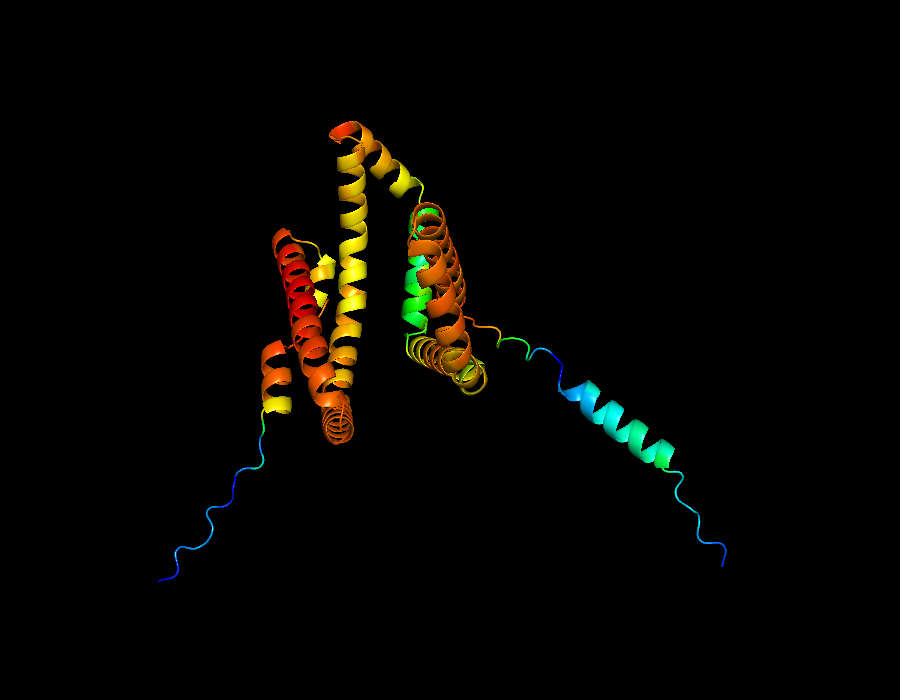

Supplement: Supplementary file 1 [file cimb-46-00701-s001.zip › Supplementary Files/File S1/ZM_NP_0013074041.png]

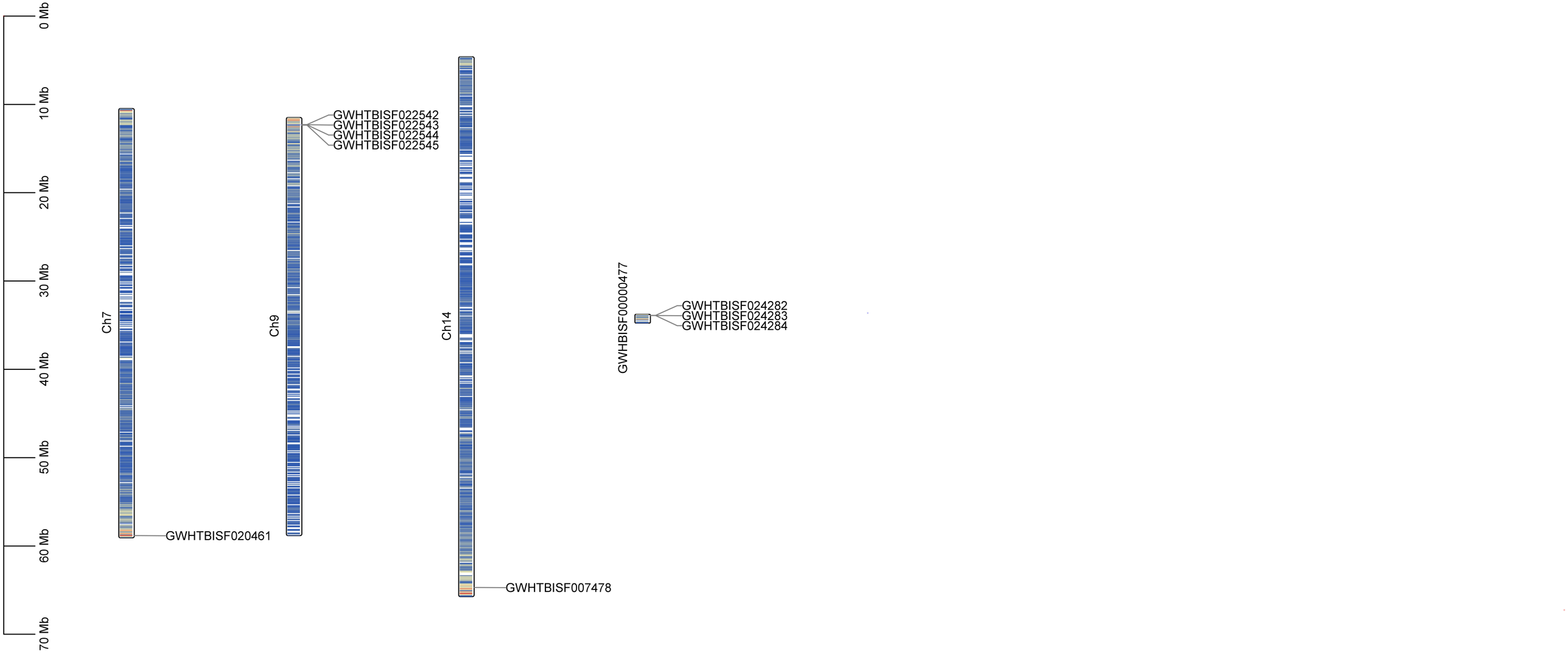

Supplement: Supplementary file 1 [file cimb-46-00701-s001.zip › Supplementary Files/File S2/Eucommia Ulmoides.jpg]

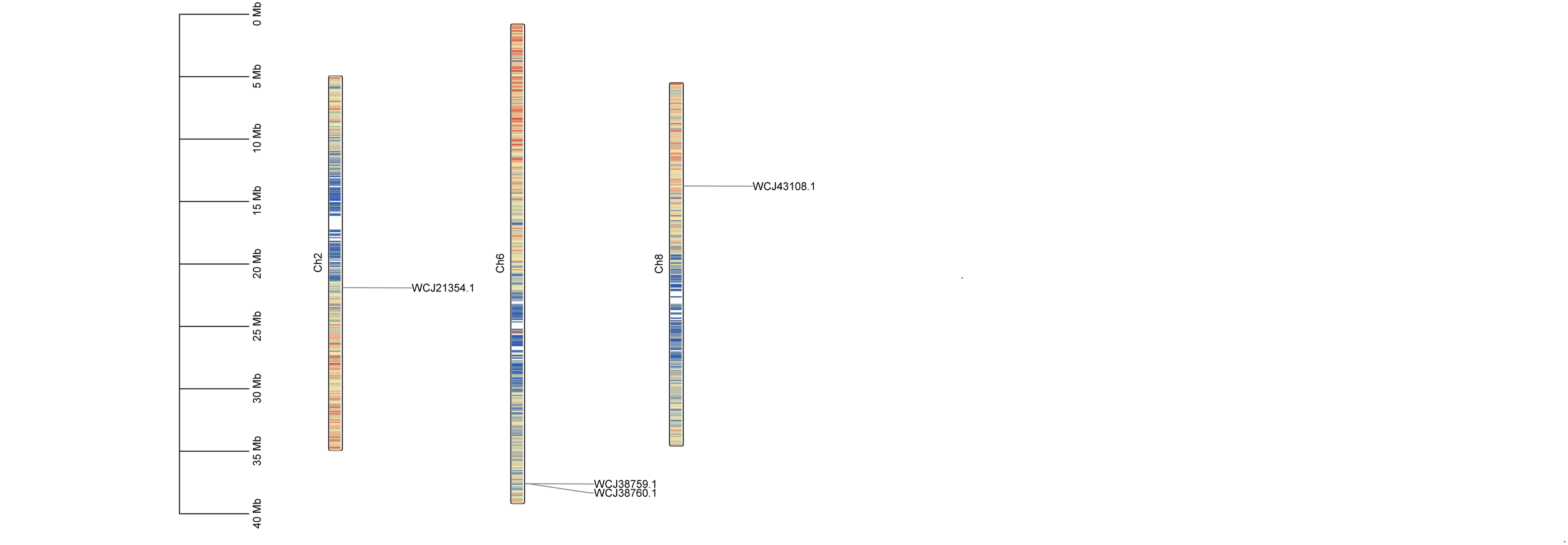

Supplement: Supplementary file 1 [file cimb-46-00701-s001.zip › Supplementary Files/File S2/Euphorbia peplus.jpg]

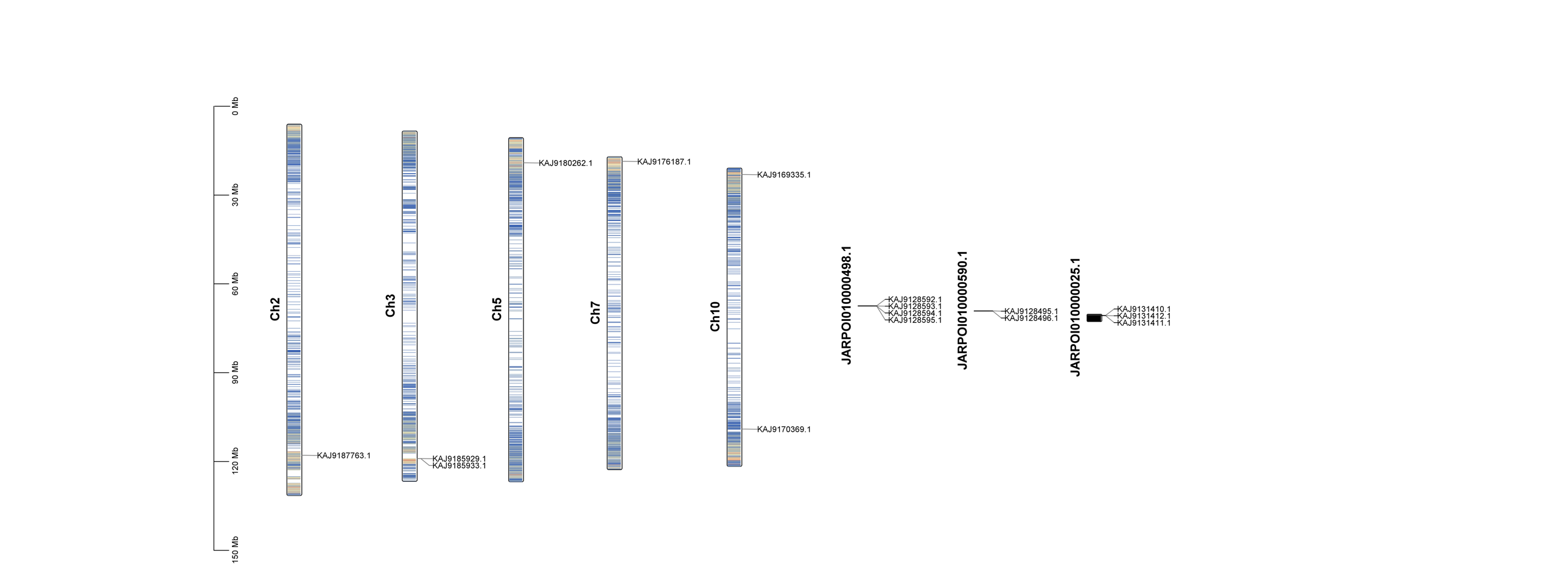

Supplement: Supplementary file 1 [file cimb-46-00701-s001.zip › Supplementary Files/File S2/Hevea brasiliensis.jpg]

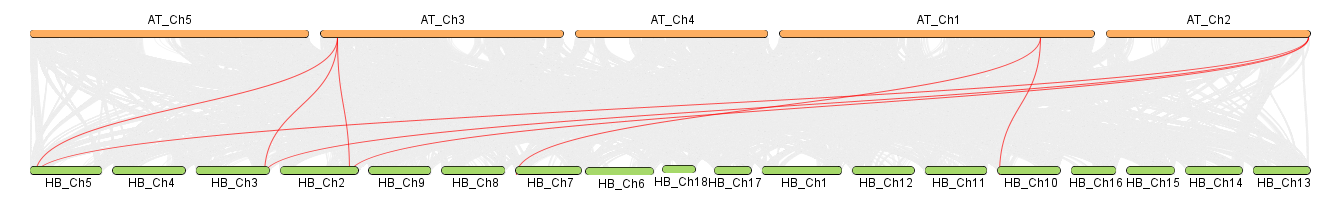

Supplement: Supplementary file 1 [file cimb-46-00701-s001.zip › Supplementary Files/File S3/Arabidopsis thaliana and Hevea brasiliensis.tif]

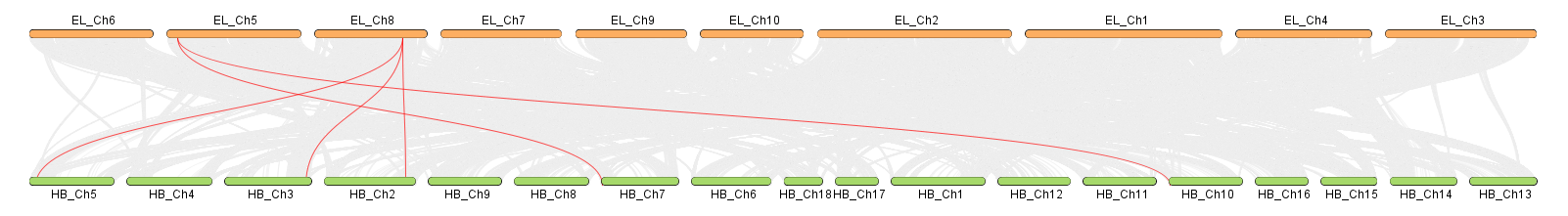

Supplement: Supplementary file 1 [file cimb-46-00701-s001.zip › Supplementary Files/File S3/Euphorbia lathyris and Hevea brasiliensis.png]

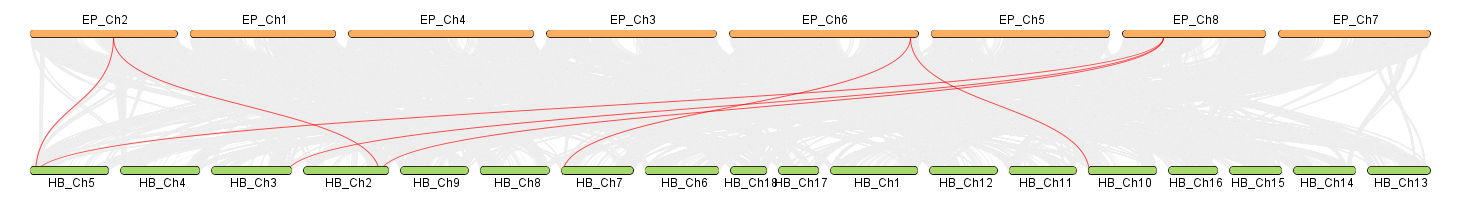

Supplement: Supplementary file 1 [file cimb-46-00701-s001.zip › Supplementary Files/File S3/Euphorbia peplus and Hevea brasiliensis.png]

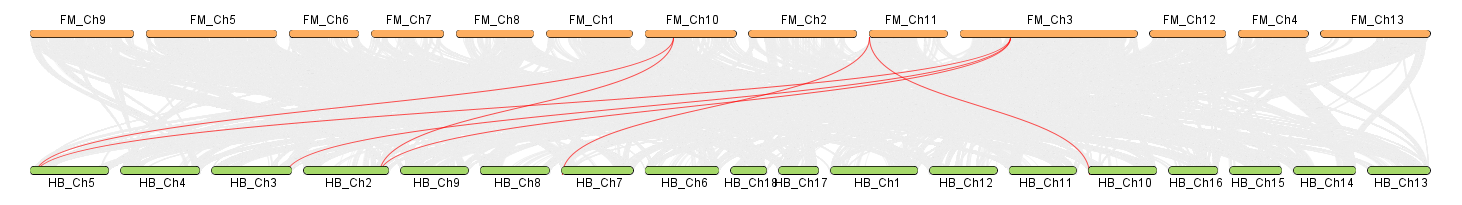

Supplement: Supplementary file 1 [file cimb-46-00701-s001.zip › Supplementary Files/File S3/Ficus microcarpa and Hevea brasiliensis.png]

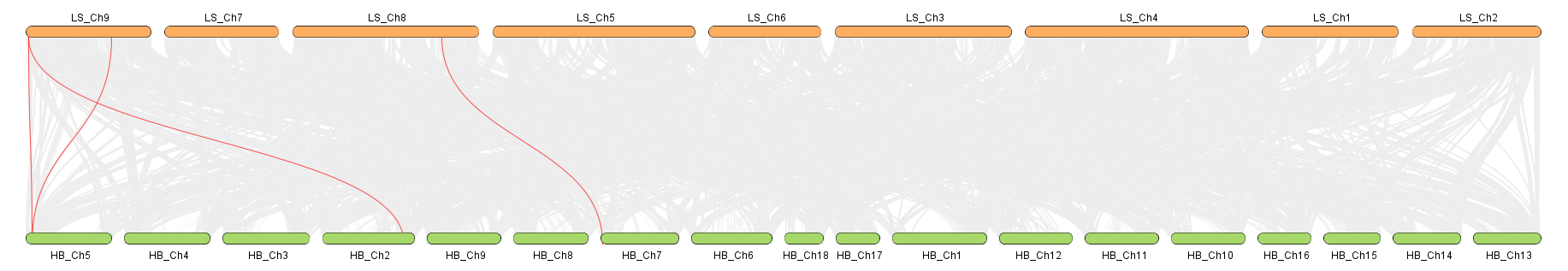

Supplement: Supplementary file 1 [file cimb-46-00701-s001.zip › Supplementary Files/File S3/Hevea brasiliensis and Lactuca sativa.png]

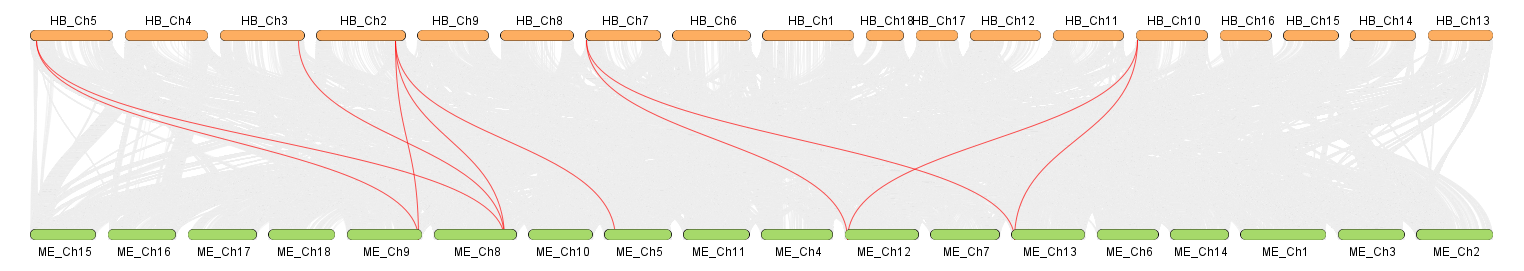

Supplement: Supplementary file 1 [file cimb-46-00701-s001.zip › Supplementary Files/File S3/Manihot esculenta and Hevea brasiliensis.tif]

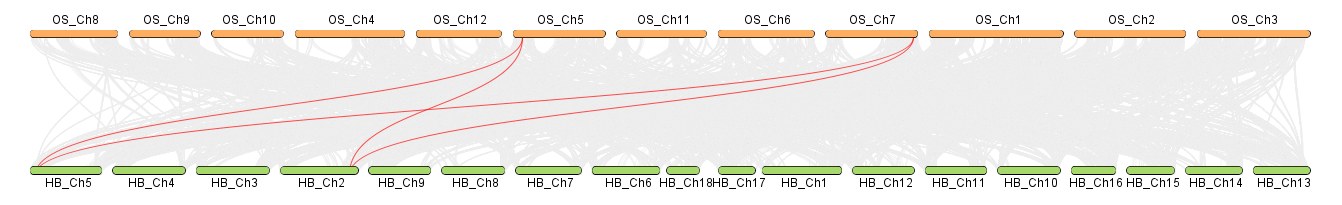

Supplement: Supplementary file 1 [file cimb-46-00701-s001.zip › Supplementary Files/File S3/Oryza sativa and Hevea brasiliensis.tif]

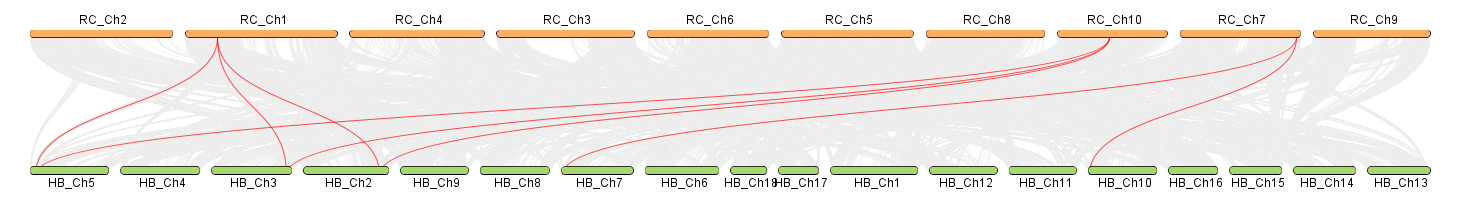

Supplement: Supplementary file 1 [file cimb-46-00701-s001.zip › Supplementary Files/File S3/Ricinus communis and Hevea brasiliensis.png]
